# Discovery of a Potent Highly Biased MOR Partial Agonist among Diastereomeric C9-Hydroxyalkyl-5-phenylmorphans

**DOI:** 10.3390/molecules28124795

**Published:** 2023-06-15

**Authors:** Joshua A. Lutz, Agnieszka Sulima, Eugene S. Gutman, Eric W. Bow, Dan Luo, Sophia Kaska, Thomas E. Prisinzano, Carol A. Paronis, Jack Bergman, Gregory H. Imler, Andrew T. Kerr, Arthur E. Jacobson, Kenner C. Rice

**Affiliations:** 1 Drug Design and Synthesis Section, Molecular Targets and Medications Discovery Branch, Intramural Research Program, National Institute on Drug Abuse and the National Institute on Alcohol Abuse and Alcoholism, National Institutes of Health, Department of Health and Human Services, 9800 Medical Center Drive, Bethesda, MD 20892-3373, USA; joshua.lutz@nih.gov (J.A.L.); agnieszka.sulima@nih.gov (A.S.); esg5026@gmail.com (E.S.G.); eric.bow@fda.hhs.gov (E.W.B.); 2 Department of Pharmaceutical Sciences, College of Pharmacy, University of Kentucky, 789 S. Limestone Street, Lexington, KY 40536, USA; dan.luo@uky.edu (D.L.); sophia.kaska.phd@gmail.com (S.K.); prisinzano@uky.edu (T.E.P.); 3 McLean Hospital/Harvard Medical School, 115 Mill Street, Belmont, MA 02478, USA; cparonis@mclean.harvard.edu (C.A.P.); jack_bergman@hms.harvard.edu (J.B.); 4 Center for Biomolecular Science and Engineering, Naval Research Laboratory, Washington, DC 20375-0001, USA; greg.imler@gmail.com (G.H.I.); andrew.kerr@nrl.navy.mil (A.T.K.)

**Keywords:** diastereomers, C9-hydroxyalkyl 5-phenylmorphans, *m*-hydroxy-*N*-phenethyl-5-phenylmorphans, *N*-phenethyl-2-azabicyclo[3.3.1]nonan-5-yl)phenols, mu-opioid receptor (MOR), delta-opioid receptor (DOR), kappa-opioid receptor (KOR) ligands, partial MOR agonist, respiratory depression, antinociceptive activity, forskolin-induced cAMP accumulation assay, G-protein bias, beta-arrestin2

## Abstract

All possible diastereomeric C9-hydroxymethyl-, hydroxyethyl-, and hydroxypropyl-substituted 5-phenylmorphans were synthesized to explore the three-dimensional space around the C9 substituent in our search for potent MOR partial agonists. These compounds were designed to lessen the lipophilicity observed with their C9-alkenyl substituted relatives. Many of the 12 diastereomers that were obtained were found to have nanomolar or subnanomolar potency in the forskolin-induced cAMP accumulation assay. Almost all these potent compounds were fully efficacious, and three of those chosen for in vivo evaluation, **15**, **21**, and **36**, were all extremely G-protein biased; none of the three compounds recruited beta-arrestin2. Only one of the 12 diastereomers, **21** (3-((1*S*,5*R*,9*R*)-9-(2-hydroxyethyl)-2-phenethyl-2-azabicyclo[3.3.1]nonan-5-yl)phenol), was a MOR partial agonist with good, but not full, efficacy (E_max_ = 85%) and subnanomolar potency (EC_50_ = 0.91 nM) in the cAMP assay. It did not have any KOR agonist activity. This compound was unlike morphine in that it had a limited ventilatory effect in vivo. The activity of **21** could be related to one or more of three well-known theories that attempt to predict a dissociation of the desired analgesia from the undesirable opioid-like side-effects associated with clinically used opioids. In accordance with the theories, **21** was a potent MOR partial agonist, it was highly G-protein biased and did not attract beta-arrestin2, and it was found to have both MOR and DOR agonist activity. All the other diastereomers that were synthesized were either much less potent than **21** or had either too little or too much efficacy for our purposes. It was also noted that a C9-methoxymethyl compound with 1*R*,5*S*,9*R* stereochemistry (**41**) was more potent than the comparable C9-hydroxymethyl compound **11** (EC_50_ = 0.65 nM for **41** vs. 2.05 nM for **11**). Both **41** and **11** were fully efficacious.

## 1. Introduction

For the past century chemists have sought compounds capable of acting as potent analgesics while limiting or eliminating the unwanted side-effects that make the current clinically used opioids problematic. Advances in our understanding of G-protein-coupled receptors (GPCRs) [1], especially the µ-opioid receptor (MOR), have enabled a number of hypotheses that aid the attempt to dissociate analgesia from unwanted side-effects and that provide insight into the cause of the dissociation. When activated using traditional opioids (e.g., morphine, oxymorphone), the MOR begins a cascade of events culminating in analgesia and that in part also recruits β-arrestin2 proteins to the receptor [2]. It has been theorized that this recruitment and the subsequent signaling may be largely responsible for the unwanted side-effects of opioids [3]. The bias of G-protein-activated signaling cascades versus cascades resulting from β-arrestin2 recruitment has been used as a pharmacological marker in the pursuit of safer analgesics [4,5], and some G-protein-biased agonists have been found to be capable of activating the MOR with reduced β-arrestin2 recruitment in vitro [6]. One such compound, oliceridine IV, has completed phase 3 human trials [7,8]. It was approved for human use in 2020, showing reduced side-effects, in comparison with the side-effects produced by opioids such as oxymorphone or oxycodone.

An alternate theory from Gillis et al. suggested that a compound’s intrinsic efficacy at the MOR may be responsible for the presence or lack of deleterious side-effects [9]. These authors indicated that the β-arrestin2 recruitment bias factor was not responsible for an analgesic’s side-effects. However, Bohn et al. later showed that activation biased against β-arrestin2 recruitment could still play a positive role in limiting opioid-like side-effects [10]. These are not the only two theories that have been promulgated. Experimental evidence has also been presented for the hypothesis that compounds acting as agonists or antagonists at the DOR can modulate the actions of MOR agonists and repress or eliminate an analgesic’s side-effects [11,12,13]. It is also interesting to note that a (mostly) DOR–KOR agonist has been shown to have fewer side-effects, including less respiratory depression, than current clinically used analgesics [14]. The role that biased signaling, intrinsic efficacy, and DOR and KOR interactions play in the analgesic activity and the side-effects of MOR agonists remains unclear. What is apparent is that to continue to assess these hypotheses we will need additional MOR partial agonists displaying a range of efficacies, G-protein-biased MOR agonists, and bifunctional compounds that interact with the MOR and DOR. These will be needed to support one, or a combination, of these theories. This report features a C9-hydroxyethyl compound (**21**) that may be helpful for these theories in that the compound has properties that can fit three of them. It is a MOR potent partial agonist with good, but not full, efficacy, it is highly biased towards G-protein signaling, and it interacts at the MOR and DOR as a bifunctional agonist. This unusual compound has been experimentally found to have less effect on respiration in vivo than morphine, a very important side-effect that is responsible for many deaths from narcotic overdose.

## 2. Results and Discussion

### 2.1. Molecular Design

**Figure 1:**
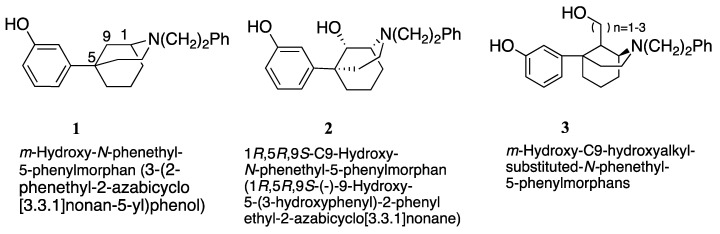
m-Hydroxy-N-phenethyl-5-phenylmorphans.

We have recently reported on G-protein-biased MOR agonists that do not recruit β-arrestin2 in a structural class known as 5-(3-hydroxy-phenyl)morphans (**1**) substituted with moieties at C9 (Figure 1) [15,16].

Our initial investigation of *N*-phenethyl-substituted 5-phenylmorphans (**1**, Figure 1) led to the discovery of C9-hydroxyphenylmorphan **2** (Figure 1) that was found to be ca 500 times more potent than morphine in rodent antinociceptive assays [17]. All four diastereomers of C9-hydroxyl-5-phenylmorphan were reported, and the 1*R*,5*R*,9*S*-stereoisomer **2** was the only one of the four that had high affinity and potency at the MOR [17], an indication of the remarkable effect that the stereochemistry about the C9 bond could have on MOR activity. These earlier findings led us to an exploration of enantiomers with a three-carbon chain at C9, which resulted in the discovery of a C9-hydroxypropyl-substituted compound that was a selective, low efficacy, high G-protein biased, partial MOR agonist that did not recruit β-arrestin2 [16]. This finding prompted our desire to explore a complete set of diastereomeric C9-hydroxyalkyl phenylmorphans, especially since these hydroxyalkyl compounds should be less lipophilic than the C9-alkenyl phenylmorphans, which is desirable. For example, the theoretical cLogP of compound **11** = 4.06, while the cLogP of the comparable C9-ethyl compound = 5.97 (via ChemDraw, version 22.0.0.22). The change from methyl to hydroxyl resulted in a considerable modification of lipophilicity. All 12 diastereomers of C9-hydroxylmethyl-, hydroxyethyl-, and hydroxypropyl-5-phenylmorphans have been synthesized to assess the effect of stereochemistry and chain length on their opioid receptor activity in vitro and the effect of three of the twelve diastereomers on antinociceptive activity and respiration in vivo.

### 2.2. Synthesis of C9-Hydroxyalkyl-5-Phenylmorphans and a C9-Methoxymethyl-5-Phenylmorphan

**Figure 2:**
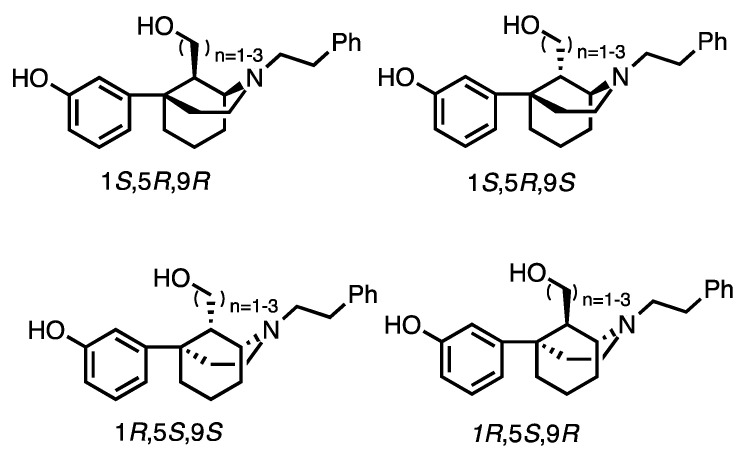
The four diastereomers of C9-hydroxyalkyl-5-phenylmorphan.

C9-substituted 5-phenylmorphans contain three chiral centers, one of which is fixed, resulting in four possible diastereomers for each unique C9 derivative. Exploration of the spatial area described by these compounds to determine their interaction with opioid receptors required the synthesis of all four diastereomers per unique C9-functionalized derivative (Figure 2).

**Scheme 1:**
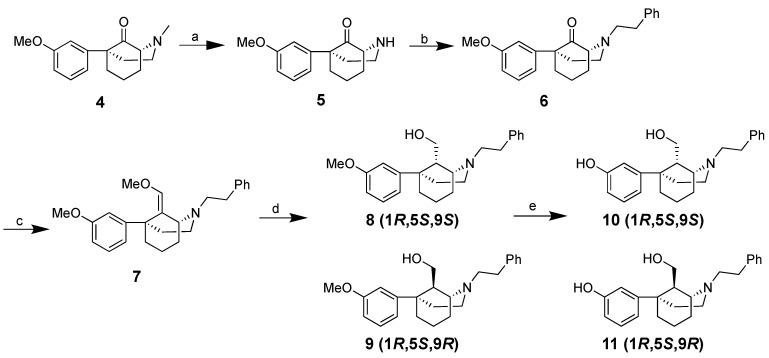
Synthesis of the 1R,5S,9S- and 1R,5S,9R-C9-hydroxymethyl-5-phenylmorphan diastereomers **10** and **11**. Reagents and conditions: (**a**) i. CNBr, K_2_CO_3_, MeCN, reflux 4 h, ii. 3 N aq. HCl, MeOH, reflux 16 h, 80%; (**b**) Ph(CH_2_)_2_Br, K_2_CO_3_, MeCN, reflux 16 h, 77%; (**c**) LiHMDS (methoxymethyl) triphenylphosphonium chloride, THF, 0 °C, 65%; (**d**) i. 4 N aq. HCl, THF, 16 h, ii. NaCNBH_3_, THF, 1 h, 60%, 3.6:1 **9** (9*R*):**8** (9*S*); (**e**) BBr_3_, DCM, −78 °C→rt, 16 h, 70%.

Synthesis of the C9-hydroxymethyl diastereomers began with enantiomerically pure 9-keto-(1*R*,5*R*)-5-phenylmorphan **4** (Scheme 1). Its synthesis [18] was previously optimized [19] and optically resolved. *N*-Demethylation to **5** was accomplished in 60% yield over two steps using a von Braun reaction followed by hydrolysis of the resulting cyanamide [20].

**Scheme 2:**
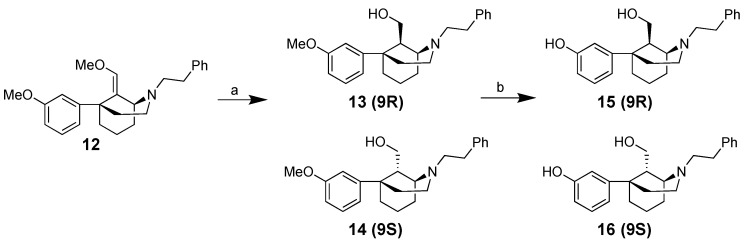
Synthesis of the 1S,5R,9R- and 1S,5R,9S-C9-hydroxymethyl-5-phenylmorphan diastereomers **15** and **16**. Reagents and conditions: (**a**) i. 3 N aq. HCl, THF, 16 h, ii. NaBH_4_, MeOH, 16 h, 69%, 1:1.5 **13** (**9R**):**14**(**9S**); (**b**) BBr_3_, DCM, −78 °C→rt, 18 h, **13** to **15**:81%, **14** to **16**: 70%.

The secondary amine **5** was alkylated using phenethyl bromide to give **6** and this was followed by a Wittig olefination to provide enol ether **7** in good yield. Hydrolysis of the enol ether and reduction of the resulting aldehyde were carried out and the diastereomers **8** and **9** could be isolated chromatographically. The stereochemistry of these compounds was based on the previously determined structures of the C9-propyl [16] and C9-alkenyl [21] compounds that had identical starting materials and intermediates **4–7**. *O*-Demethylation of ethers **8** and **9** gave the desired alcohols 1*R*,5*S*,9*S*-**10** and 1*R*,5*S*,9*R*-**11**. Pure material was obtained as a colorless oil and crystallized as the hydrobromide salt. The opposite enantiomer (9-keto-(1*S*,5*S*)-5-phenylmorphan) was used as a starting material to generate enol ether **12** and similar conditions were applied to give alcohols **15** and **16** (Scheme 2).

**Figure 3:**
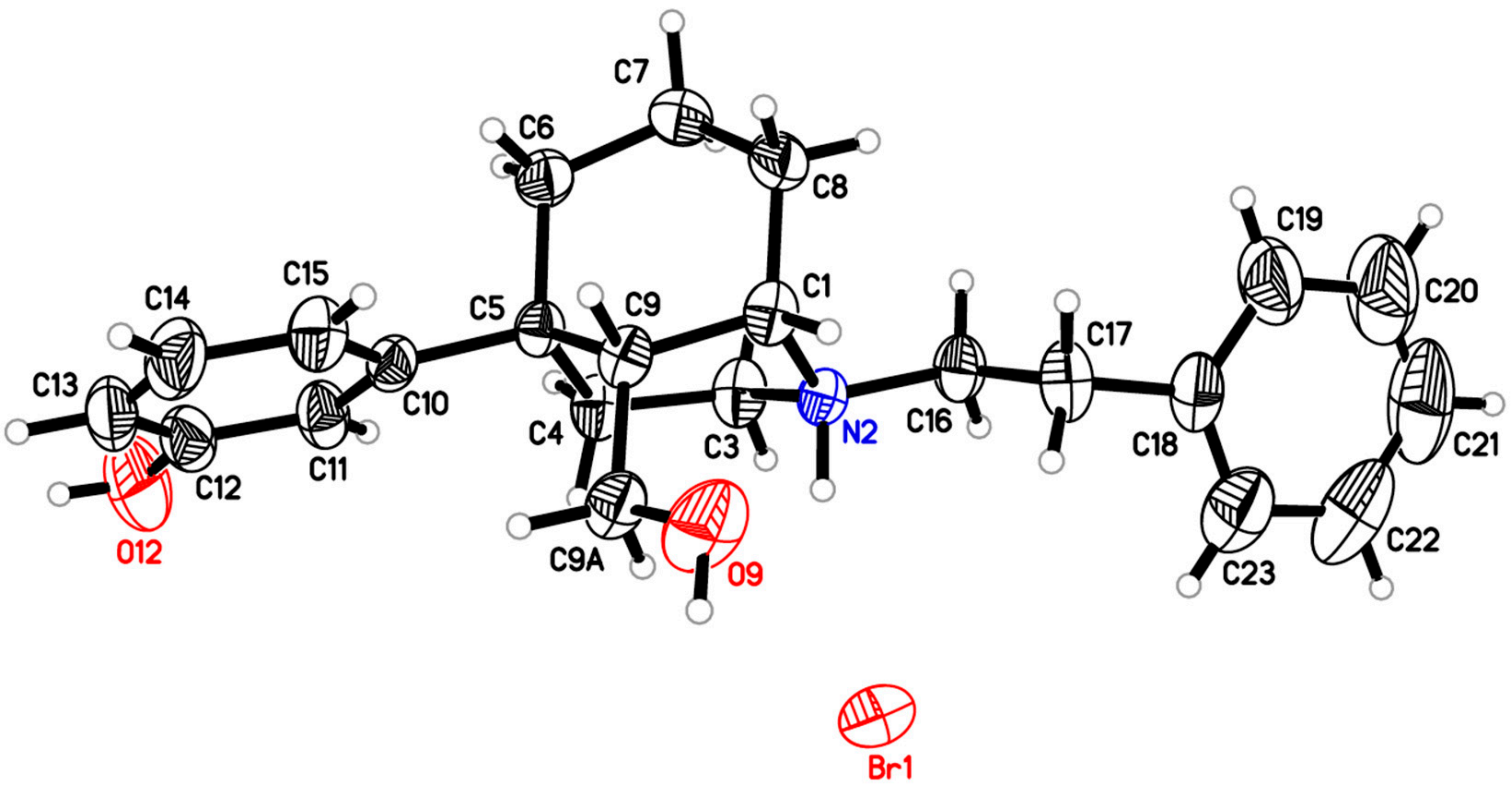
X-ray crystallographic structure of 3-((1*S*,5*R*,9*R*)-9-(hydroxymethyl)-2-phenethyl-2-azabicyclo[3.3.1]nonan-5-yl)phenol (**15**). The ellipsoids are shown at the 50% level. The crystal data, atomic coordinates, etc, have been cited in the Supplementary Materials.

The relative configuration at the C9 position was determined by an X-ray crystallographic analysis of the hydrobromide salt of **15** (Figure 3).

**Scheme 3:**
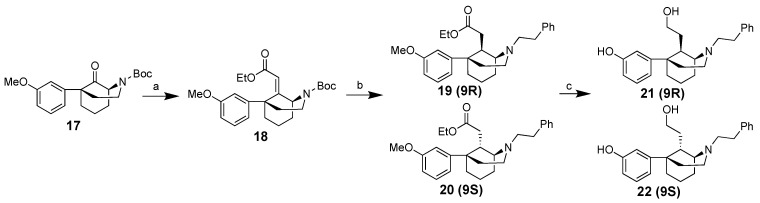
Synthesis of 1*S*,5*R*,9*R* and 1*S*,5*R*,*9S*-C9-hydroxyethyl-5-phenylmorphan diastereomers **21** and **22**. Reagents and conditions: (**a**) NaH, (diethoxyphosphoryl)acetate, THF, reflux, 88% (**b**) i. H-Cube flow reactor, 10% Pd/C, iProAc, 80 °C, H_2_, 45 psi ii. Triflurooacetic acid, DCM, 0 °C→rt, 1 h, iii. Ph(CH_2_)_2_Br, K_2_CO_3_, MeCN, reflux 16 h, 62% over 3 steps, 5:1 **19 (9R):20 (9S)**; (**c**) i. LiAlH_4_, THF, 0 °C→rt, 1 h, ii. BBr_3_, DCM, −78 °C→rt, 1 h, 44% for **21** and 38% for **22** over 2 steps.

With the four C9-hydroxymethyl-5-phenylmorphan diastereomers in hand (Scheme 1 and Scheme 2), we shifted our focus to the C9-hydroxyethyl compounds. The most direct route began with a Horner–Wadsworth–Emmons olefination on ketone **6**, which contains a phenethyl substitution on the piperidine nitrogen. However, we discovered this olefination and subsequent hydrogenation did not produce appreciable amounts of the *9R* ester **19**, instead giving almost exclusively the opposite *9S* diastereomer **20**. To obtain both isomers in useful quantities, we installed a sterically bulky tert-butylcarbonyl (BOC) group on the piperidine nitrogen to give ketone **17** (Scheme 3). Olefination to give α,β-unsaturated ester **18** proceeded in high yield. We found direct reduction of *N*-Boc **18** led to useful amounts of both diastereomers with selectivity for the *9R* ester **19**. We proceeded with this route.

Conversion of olefin **18** to esters **19** and **20** was attempted using a Parr shaker for hydrogenation, however, this compound was found to be too sterically hindered to effectively hydrogenate in this manner. Typical conditions using 1–10% *w*/*w* catalyst loading led to 50–60% recovery of starting material. To effectively reduce **18**, we needed to increase both the temperature of the reaction and the catalyst loading.

**Scheme 4:**
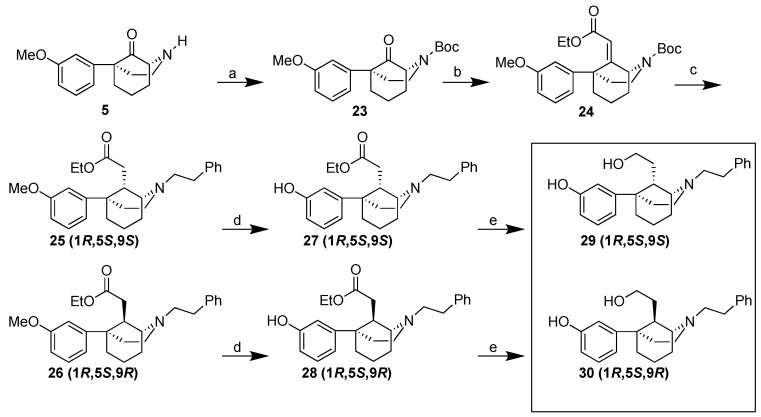
Synthesis of *1R*,5*S*,9S- and *1R*,5*S*,9*R*-C9-hydroxyethyl-5-phenylmorphan diastereomers **29** and **30**. Reagents and conditions: (**a**) Boc_2_O, triethylamine, DMAP, DCM, 78%; (**b**) NaH, triethyl phosphonoacetate, THF, reflux, 16 h, 95%; (**c**) i. Escat 103 (5% Pd/C), 60 °C, 50 psi, 1 h, ii. triflouroacetic acid, DCM, 0 °C, 1 h, iii. Ph(CH_2_)_2_Br, K_2_CO_3_, MeCN, reflux 18 h, **80**% over 3 steps, **1:3 25** (1*R*,5*S*,9*S*):**26** (1*R*,5*S*,9*R*); (**d**) BBr_3_, DCM, −78 °C→rt, 1 h, 98% (**e**) LiAlH_4_, THF, 0 °C, 1 h, 47% **29** (1*R*,5*S*,9*R*)**,** 66% **30** (1*R*,5*S*,9*S*).

Full hydrogenation was accomplished using a Thales-Nano H-Cube Pro flow reactor with isopropyl acetate, heating to 80 °C. With this method, complete conversion of starting material was observed and a favorable 5:1 selectivity achieved, isolating **19** in 51% and **20** in 11% yield over the three steps from compound **18** (Scheme 4). Esters **19** and **20** were reduced with lithium aluminum hydride and converted to phenols **21** and **22** using boron tribromide in 44% and 38%, respectively, over two steps to give the desired C9-hydroxyethyl diastereomers. Both diastereomers were crystallized as hydrobromide salts. The synthesis of the remaining 1*R*,*5S*,9*S* and 1*R*,*5S*,9*R* hydroxyethyl diastereomers **29** (9*S*) and **30** (9*R*) was accomplished using the opposite, *1R*,5*S*, piperidine enantiomer that was needed for the diastereomers **21** and **22**. In the case of these compounds, *O*-demethylation was performed at an earlier stage to give phenol **23** followed by typical conditions to transform the α,β-unsaturated esters to their respective alcohols (Scheme 4). The synthesis of **29** (1*R*,5*S*,9S) and **30** (1*R*,5*S*,9*R*) completed the set of all four C9-hydroxyethyl diastereomers.

**Scheme 5:**
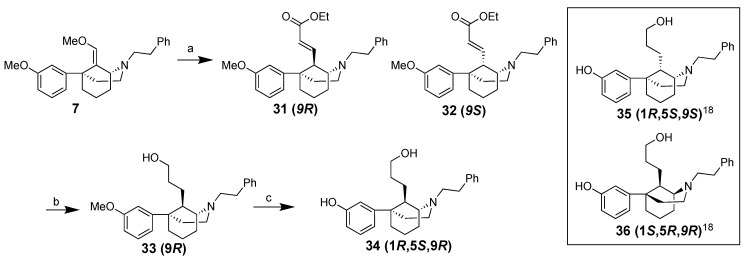
Synthesis of 1*R*,5*S*,9*R*-C9-hydroxypropyl-5-phenylmorphan **34**. Reagent and conditions: (**a**) i. 6 N aq. HCl, THF, 16 h, ii. NaH, triethyl phosphonoacetate, THF, reflux, 16 h, 75%, 1.2:1 **31 (9R):32 (9S)**; (**b**) i. 5% Pd/C, EtOH, 50 °C, H_2_, 50 psi, 1 h, ii. LiAlH_4_, THF, 0 °C→rt, 1 h, 89%; (**c**) BBr_3_, DCM, −78 °C→rt, 1 h, 36%.

With the synthesis of the four 9-hydroxymethyl and 9-hydroxylethyl diastereomers completed, we turned our attention to their 9-hydroxypropyl relatives. Synthesis began with hydrolysis of enol ether **7**, followed by a Horner–Wadsworth–Emmons olefination to give enoates **31** and **32** in good yield, with only slight selectivity for the stereochemistry at C9 (Scheme 5). At this step, the C9*-R* (**31**) and the C9-*S* (**32**) diastereomers were separated. Further reaction of intermediate **31** is shown in Scheme 5 to give phenol **34**. Reductions of both the olefin and the ethyl ester of **31** were achieved with catalytic hydrogenation and lithium aluminum hydride, respectively, to give alcohol **33** in high yield. *O*-Demethylation of **33** gave a side-product in significant yield (39%) that was difficult to characterize and led to poor yield of desired phenol **34**. Despite this, enough material was acquired to crystallize the desired product as the hydrobromide salt. The syntheses of the 1*R*,5*S*,9*S*-diastereomer **35** along with its enantiomer 1*S*,5*R*,9*R*-**36** have been previously described [16] and are included in Table 1 for comparison purposes.

**Scheme 6:**
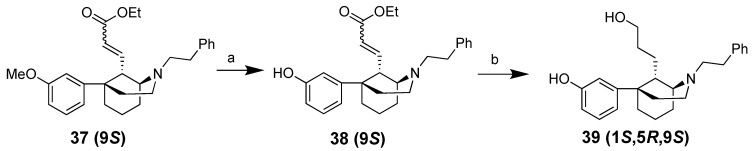
Improved synthesis for 1S,5R,9S-C9-hydroxypropyl-5-phenylmorphan **39**. Reactions and conditions: (**a**) BBr_3_, DCM, −78 °C→rt, 1 h, 59%; (**b**) i. 5% Pd/C, EtOH, H_2_, 50 psi, rt, 1 h, ii. LiAlH_4_, THF, 0 °C→rt, 1 h, 72%.

We sought to improve the synthetic route in Scheme 6 when synthesizing **39**, the 1*S*,5*R*,9*S*-enantiomer of 1*R*,5*S*,9*R*-**34**. Since the *O*-demethylation proceeded in poor yield, we elected to *O*-demethylate enoate **37** before moving on to the hydrogenation step (Scheme 6). This change improved the yield of the demethylation. Phenol **38** was hydrogenated with a palladium catalyst and the ester group was reduced with lithium aluminum hydride. The desired product **39** was obtained in 72% from phenolic enolate **38**. Applying these conditions to the opposite diastereomer yielded similar results, completing the synthesis of all four diastereomers of the C9-hydroxypropyl derivatives.

**Scheme 7:**
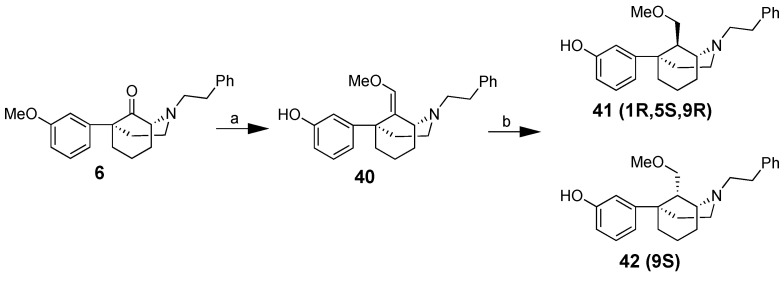
Synthesis of 1*R*,5*R*,9*R*-C9-methoxymethyl-5-phenylmorphan **41**. Reagents and condition: (**a**) i. BBr_3_, DCM, −78 °C→rt, 1 h, 75%, ii. LiHMDS (methoxymethyl) triphenylphosphonium chloride, THF, 0 °C, 21%; (**b**) 5% Pd/C, EtOH, H_2_, 50 psi, rt, 1 h, 55%, 6:1, **41** (9R): **42** (9S).

Lastly, we wanted to assess the role the polar hydroxy group plays in the activity of the described compounds. To achieve this, we synthesized a methyl ether analog of **11**. We *O*-demethylated ketone **6** followed by a Wittig reaction to give phenolic enol ether **40** (Scheme 7) [15]. Subsequent catalytic hydrogenation led to a favorable selectivity for the desired ether 1*R*,5*R*,9*R*-**41**.

### 2.3. Forskolin-Induced cAMP Accumulation Assay for In Vitro Determination of the Potency and Efficacy of the Diastereomers

The potency and efficacy of the nine new diastereomers and the three previously reported compounds [16] are shown in Table 1. Only the MOR potency and efficacy of a compound was determined if the MOR EC_50_ was found to be >30 nM, since those with less potency were unlikely to have useful analgesic activity. The bias factors of the MOR compounds with EC_50_ < 30 nM were also determined.

As is well known, diastereomers on a two-dimensional surface look identical, but in three dimensions they will present different faces to the amino acids in the opioid receptors, resulting in minor or major differences in their pharmacological activity. It is not possible to estimate what these pharmacological effects might be, nor is it possible to a priori calculate those effects using molecular modeling and simulation techniques, since the opioid receptors are able to twist and turn to encompass many different types of three-dimensional structures. At best, it is possible to distinguish molecularly related compounds as either MOR agonists or antagonists using molecular modeling and simulation techniques [21]. Determination of the relative potencies and efficacies of diastereomeric MOR agonists using molecular modeling is still not possible, insofar as we are aware.

In the C9-hydroxyalkyl series, both stereochemistry and chain length at C9 were found to affect potency in the cAMP assay (Table 1). Two compounds with 1*R*,5*S*,9*R* stereochemistry had subnanomolar affinity for the MOR and were fully efficacious, **30** (EC_50_ = 0.19 nM, E_max_ = 98%) and **34** (EC_50_ = 0.32 nM, E_max_ = 100%). The stereochemically comparable compound with a one-carbon chain at C9, **11**, was less potent (EC_50_ = 2.05 nM), but still had nanomolar potency and was also fully efficacious (E_max_ = 99%). Compounds with a two-carbon chain at C9 were generally the most potent diastereomers. Two diastereomers did not have any MOR agonist activity and were weak MOR antagonists (the C9-hydroxymethyl and hydroxyethyl compounds **10** and **29**). Both compounds had 1*R*,5*S*,9*S* stereochemistry. The comparable 1*R*,5*S*,9*S*-hydroxypropyl compound **35** had weak MOR agonist potency and had low efficacy (EC_50_ = 35 nM, E_max_ = 52%). All the diastereomers with C9-*R* stereochemistry were more potent than the comparable compounds with C9-*S* stereochemistry. Three of the diastereomers had good agonist potency at the DOR, **11**, **21**, and **30**, and one (**34**) had weak DOR antagonist activity and no DOR agonist activity. None of the diastereomers had any KOR agonist activity, although several had very weak KOR antagonist activity. Only one diastereomer exhibited partial agonist activity with high (subnanomolar) MOR potency, 1*S*,5*R*,9*R-***21** (EC_50_ = 0.91 nM, E_max_ = 85%). This compound, and the two other 1*S*,5*R*,9*R* compounds **15** and **36**, had extremely high G-protein bias. No beta-arrestin recruitment was observed experimentally (EC_50_ > 50,000 and E_max_ = 0) for these three compounds, thus their bias factors are not calculable when log (EC_50_/E_max_) is used in the equations. One of the main factors in the ability of a diastereomer to recruit beta-arrestin is their stereochemistry (e.g., the 1*S*,5*R*,9*R* compounds **15**, **21**, and **36**). The bias factors that were determined for the other diastereomers (**11**, **30**, **34**, and the methoxy ether **41**) were somewhat higher (better) than those for morphine and DAMGO (set to 1 by definition) and comparable to PZM21 [24]. It was of interest to note that the C9-methoxymethyl compound with 1*R*,5*S*,9*R* stereochemistry (**41**) was more potent than the comparable C9-hydroxymethyl compound **11** (EC_50_ = 0.65 nM for **41** vs. 2.05 nM for **11**). Both compounds were fully efficacious. This finding will be more fully explored in subsequent publications.

### 2.4. In Vivo Data: Antinociceptive and Respiration Assays [25] in Nonhuman Primates (NHPs) for Compounds ***15***, ***21***, and ***36***

**Figure 4:**
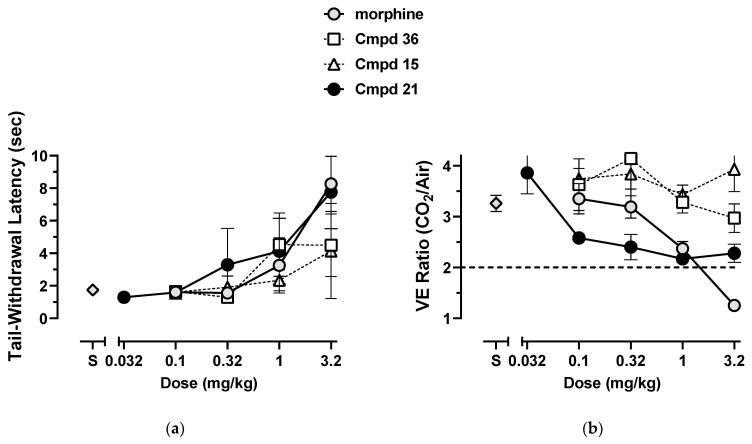
(**a**) Effects of compounds **15**, **21**, and **36** (in comparison to morphine and saline (S)) on tail withdrawal latency and, (**b**) effects on ventilatory ratio, in squirrel monkeys. Compound **21** and morphine significantly increased tail withdrawal latency and reduced the ability of 5% CO_2_ to stimulate increases in ventilation. Compounds **15** and **36** did not significantly increase tail withdrawal latency or alter ventilation. Data are expressed as mean ± SEM (*n* = 3 to 5; results of statistical analysis described in text).

Three MOR partial agonists with varied efficacy (very low efficacy (**15**), low efficacy (**36**), and good efficacy (**21**)), were examined in vivo, choices based on our focus on potent MOR partial agonists with variable efficacy as determined in the cAMP assay. Neither compound **15** nor **36** had antinociceptive effects (F_(4,15)_ = 1.75 and F_(4,10)_ = 0.57, respectively, both n.s.) in nonhuman primates (Figure 4) in accordance with their low efficacy in the cAMP assay (Table 1). Similarly, compounds **15** and **36** had limited respiratory depressant effects (i.e., changes in the ratio of minute volumes in an atmosphere of 5% CO_2_ and room air; ventilatory ratio). In contrast, compound **21** had full antinociceptive effects in three of four subjects (Figure 4). These effects were similar to those obtained with morphine (Figure 4) and approached statistical significance for the group of animals (F_(5,18)_ = 2.692, *p* = 0.055).

**Figure 5:**
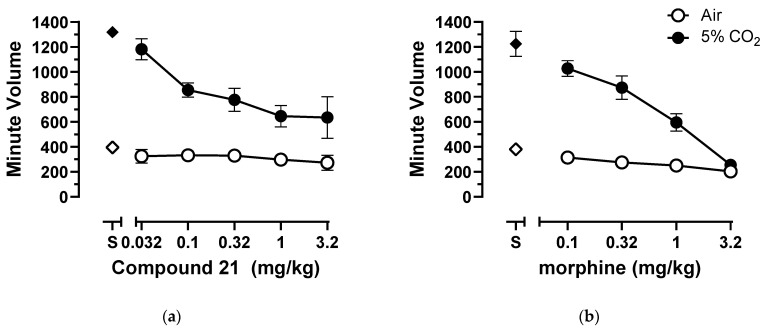
(**a**) Effects of compound **21** and, (**b**) effects of a standard drug, morphine, on minute volume (VE) in air and air mixed with 5% CO_2_ in squirrel monkeys (points above S represent effects of saline). Data are expressed as mean ± SEM (*n* = 4).

Compound **21** also decreased ventilatory ratio (F_(5,17)_ = 7.85, *p* < 0.01), however, unlike morphine, these effects appeared to plateau at a value above 2, indicating that 5% CO_2_ continued to stimulate ventilation in animals administered compound **21**. In comparison, an increasing dose of morphine produced dose-related decreases in ventilatory ratio, to values that approached 1 (Figure 4). The limited ventilatory effects of compound **21** are highlighted in Figure 5, where it is shown that 5% CO_2_ continues to stimulate increased minute volume over all doses of compound **21** whereas the functions relating morphine dose to minute volumes in air or 5% CO_2_ intersect (Figure 5).

## 3. Materials and Methods

### 3.1. General Information

Melting points were determined on a Mettler Toledo MP70 and are uncorrected. Proton and carbon nuclear magnetic resonance (^1^H and ^13^C NMR) spectra were recorded on a Varian Gemini-400 spectrometer in CDCl_3_ (unless otherwise noted) with the values given in ppm (TMS as internal standard) and J (Hz) assignments of ^1^H resonance coupling. Mass spectra (HRMS) were recorded on a Waters (Mitford, MA, USA) Xevo-G X5 QTof. The optical rotation data were obtained on a PerkinElmer polarimeter model 341. Thin layer chromatography (TLC) analyses were carried out on Analtech silica gel GHLF 0.25 mm plates using various gradients of CHCl_3_/MeOH containing 1% NH_4_OH or gradients of *n*-hexane/EtOAc. Visualization was accomplished under UV light or by staining in an iodine chamber. Flash column chromatography was performed with Fluka silica gel 60 (mesh 220−400). Robertson Microlit Laboratories, Ledgewood, N.J., performed elemental analyses, and the results were within ±0.4% of the theoretical values. The NMR spectra are shown in the Supplementary Materials, Figures S1–S26.

### 3.2. Synthesis

General crystallization method for C9-hydroxy(alkyl)-5-phenylmorphan salts. The free base was dissolved in isopropanol (2–4 mL/g) to make a saturated solution. A solution of 48% HBr (1 equiv.) was added dropwise. This mixture was stirred at room temperature for 1 h. In the case of crystals not forming after 1 h, diethyl ether was added dropwise until the solution remained briefly cloudy before becoming clear again then left to stir overnight. In the case of neither method working, the isopropanol–free base solution was placed in a sealed chamber of diethyl ether to allow vapor diffusion overnight. Obtained solid was recrystallized from 8% methanol in isopropanol (10–20 mL/g) at 80 °C. The solution was allowed to slowly cool to room temperature.

*(1R,5R)-5-(3-Methoxyphenyl)-2-azabicyclo[3.3.1]nonan-9-one* (**5**). Tertiary amine **4** (408 mg, 1.573 mmol, 1.0 equiv) was dissolved in dry acetonitrile (10 mL) and to this solution was added K_2_CO_3_ (435 mg, 3.146 mmol, 2.0 equiv) followed by cyanogen bromide (472 µL of a 5.0 M solution in acetonitrile, 3.146 mmol, 2.0 equiv). The reaction was stirred at room temperature for 2 h before being brought to reflux for 1 h. Methanol (1.5 mL) was added and stirred for 10 min. Solvent was removed and the residue taken up in CHCl_3_ (20 mL) and washed with water (15 mL). The organic layer was dried with MgSO_4_. Chloroform was removed under vacuum and the residue was dissolved in 3N HCl (10 mL) and heated at reflux for 16 h. The reaction mixture was transferred to a separatory funnel and made basic (pH > 10.5) with 2 M KOH. The aqueous layer was extracted with CHCl_3_ (20 mL × 2) and the organic layer was dried over MgSO_4_ and concentrated. The crude residue was purified via flash chromatography eluting with 0–10% 50:45:5 CHCl_3_:MeOH:NH_4_OH in CHCl_3_ to yield **9** as a brown oil (197 mg, 51%). Data for **5** were consistent with compound **4** in reference [17].

*(1R,5R)-5-(3-Methoxyphenyl)-2-phenethyl-2-azabicyclo[3.3.1]nonan-9-one* (**6**). Secondary amine **5** (404 mg, 1.647 mmol, 1.0 equiv) was dissolved in dry acetonitrile (10 mL) and to this solution was added K_2_CO_3_ (455 mg, 3.294 mmol, 2.0 equiv) followed by phenethyl bromide (457 mg, 337 µL, 2.47 mmol, 1.5 equiv). The solution was brought to reflux and left under N_2_ for 16 h. The mixture was filtered through celite, concentrated, and purified by flash chromatography eluting with 0–10% 50:45:5 CHCl_3_:MeOH:NH_4_OH to yield **6** as a colorless foam (445 mg, 77%). Data for **6** were consistent with compound **5** in reference [17].

*(1R,5R, E&Z)-9-(Methoxymethylene)-5-(3-methoxyphenyl)-2-phenethyl-2-azabicyclo [3.3.1]nonane* (**7**). To as suspension of tertiary amine 6 (445 mg, 1.273 mmol, 1.0 equiv) and (methoxymethyl)triphenylphosphonium chloride (1.310 g, 3.820 mmol, 3.0 equiv) in dry tetrahydrofuran (6 mL) at 0 °C was added LiHMDS (3.31 mL of 1.0 M solution in THF, 2.6 equiv) dropwise over 10 min. After 30 min the ice bath was removed, and the solution was stirred under argon for 16 h. The mixture was cooled to 0 °C and methanol (4.5 mL) was added and stirred for 10 min. The solvents were removed under vacuum and the residue was taken up in CHCl_3_ (50 mL) and washed with water which was made basic (pH > 10.5) with NH_4_OH. The combined organic extracts were washed with brine, dried over MgSO_4_, concentrated, and purified via flash chromatography eluting with 25–100% ethyl acetate in *n*-hexane to give 7 as a yellow oil (413 mg, 86%). Data matched compound 12 in reference [15].

*((1R,5S,9R & 1R,5S,9S)-5-(3-Methoxyphenyl)-2-phenethyl-2-azabicyclo[3.3.1]nonan-9-yl)methanol (***8** & **9**). A mixture of the *E* and *Z* isomers of enol ether **7** (810 mg, 2.146 mmol, 1.0 equiv) in dry tetrahydrofuran (8 mL) was added dropwise to a stirred solution of 4 N HCl (8 mL) and this solution was stirred under argon for 16 h. Methanol (5 mL) was added dropwise, and the reaction was stirred for 15 min before removing volatile solvents under vacuum. The aqueous mixture was cooled to 0 °C and made basic (pH > 10.5) with NH_4_OH and extracted with 9:1 CHCl_3_:MeOH (20 mL × 2). The organic extracts were washed with brine, dried over MgSO_4_, and solvent removed in vacuo. The crude residue was taken directly to the next step. The crude material was dissolved in tetrahydrofuran (8 mL) and this solution was cooled to 0 °C. To the cooled solution was added NaCNBH_3_ (192 mg, 3.219 mmol, 1.5 equiv) and the solution was stirred at 0 °C for 10 min. The ice bath was removed and the solution stirred at room temperature for 1 h. The solution was again cooled to 0 °C and water (10 mL) added dropwise. The mixture was diluted with CHCl_3_ (25 mL) and the aqueous layer was made basic (pH > 10.5) with NH_4_OH and extracted with 9:1 CHCl_3_:MeOH (20 mL × 2). The organic extracts were washed with brine, dried over MgSO_4_, and solvent removed in vacuo. The crude residue was purified via flash chromatography eluting with 0.5–10% 50:45:5 CHCl_3_:MeOH:NH_4_OH to yield **8** as a teal oil and **9** as a yellow foam in a 3.6:1 diastereomeric ratio (369 mg of **9**, 47% and 103 mg of **8**, 13%).

*For 1R,5S,9S-***8**: ^1^H-NMR (400 MHz; CDCl_3_): *δ* 7.31–7.18 (m, 6H), 7.04–6.97 (m, 2H), 6.73 (dd, *J* = 8.1, 2.1 Hz, 1H), 3.79 (s, 3H), 3.53 (t, *J* = 10.5 Hz, 1H), 3.41 (dd, *J* = 11.2, 4.5 Hz, 1H), 3.28 (s, 1H), 3.03 (dd, *J* = 10.3, 4.1 Hz, 2H), 2.91–2.80 (m, 4H), 2.50 (dt, *J* = 9.1, 4.2 Hz, 1H), 2.25 (dt, *J* = 13.6, 9.6 Hz, 1H), 1.98–1.93 (m, 4H), 1.82–1.72 (m, 2H), 1.57–1.49 (m, 1H); ^13^C-NMR (100 MHz; CDCl_3_): *δ* 159.8, 151.3, 140.9, 129.4, 128.9, 128.5, 126.1, 118.1, 112.3, 110.6, 61.3, 58.4, 55.3, 53.1, 49.9, 48.6, 41.7, 37.5, 34.9, 29.7, 21.9; HRMS-ESI (*m*/*z*): [M +H]^+^ calcd for C_24_H_31_NO_2_: 366.2433; found: 366.2433.

*For 1R,5S,9R-***9**: ^1^H-NMR (400 MHz; CDCl_3_): *δ* 7.30–7.17 (m, 6H), 6.92–6.88 (m, 2H), 6.71 (dd, *J* = 8.1, 2.0 Hz, 1H), 3.78 (s, 3H), 3.64 (dd, *J* = 11.2, 4.0 Hz, 1H), 3.57 (dd, *J* = 10.8, 3.2 Hz, 1H) 3.30 (s, 1H), 3.09–3.15 (m, 2H), 2.88–2.71 (m, 5H), 2.32–2.27 (m, 1H), 2.05–1.83 (m, 4H), 1.75–1.68 (m, 2H), 1.56–1.49 (m, 1H); ^13^C-NMR (100 MHz; CDCl_3_): *δ* 159.6, 150.9, 140.2, 129.2, 128.7, 128.5, 126.2, 118.1, 112.4, 110.2, 64.4, 57.0, 56.9, 55.2, 48.9, 45.2, 43.0, 38.4, 34.5, 31.3, 25.3, 23.2; HRMS-ESI (*m*/*z*): [M +H]^+^ calcd for 366.2433 C_24_H_31_NO_2_, found: 366.2433.

*3-((1R,5S,9S)-9-(Hydroxymethyl)-2-phenethyl-2-azabicyclo[3.3.1]nonan-5-yl)phenol* (**10**). A solution of **8** (247 mg, 0.676 mmol, 1.0 equiv.) in dry dichloromethane (9 mL) was brought to −78 °C under an atmosphere of argon. To this cooled solution was added BBr_3_ (677 mg, 256 µL, 2.703 mmol, 4.0 equiv.) dropwise and the solution was stirred and allowed to warm to room temperature under argon for 16 h. The solution was cooled to 0 °C and methanol (3 mL) was added dropwise and stirred for 30 min. A solution of 1N HCl (4 mL) was added, and the mixture was brought to 100 °C. After 1 h, the solution was cooled to 0 °C, made basic (pH > 10.5) with NH_4_OH, and then extracted with 9:1 CHCl_3_ and MeOH (30 mL × 2). The organic extracts were washed with brine, dried over MgSO_4_, and solvent was removed in vacuo. The crude residue was purified via flash chromatography eluting with 20–100% EtOAc in *n*-hexane to give **10** as a colorless oil (167 mg, 70%) [α]_D_^25^ −27.3° (*c* 0.55, CHCl_3_). The free base was crystallized as the hydrobromide salt from isopropanol and diethyl ether by adding 48% HBr, mp 243–245 °C. ^1^H-NMR (400 MHz; CDCl_3_): *δ* 7.30–7.09 (m, 7H), 6.79 (d, *J* = 8.7 Hz, 2H), 6.58 (dd, *J* = 7.9, 1.4 Hz, 1H), 3.68 (dd, *J* = 11.1, 4.0 Hz, 1H), 3.55 (dd, *J* = 11.2, 2.6 Hz, 1H), 3.33 (s, 1H), 3.17–3.13 (m, 2H), 2.93–2.78 (m, 5H), 2.34–2.29 (m, 1H), 2.04–1.87 (m, 5H), 1.76–1.69 (m, 2H), 1.60–1.50 (m, 1H); ^13^C-NMR (100 MHz; CDCl_3_): *δ* 156.5, 150.6, 140.0, 129.4, 128.70, 128.67, 126.4, 117.4, 113.2, 112.8, 64.7, 57.5, 56.9, 48.9, 45.0, 42.7, 38.3, 34.4, 31.3, 25.3, 23.2; HRMS-ESI (*m*/*z*): [M +H]^+^ calcd for C_23_H_29_NO_2_ 352.2277; found: 352.2274. HBr salt: mp: 243–245 °C; anal. calcd for C_23_H_30_BrNO_2_·0.5 H_2_O: C, 62.62%; H, 7.02%; N, 3.19%. Found C, 62.58%; H, 7.08%; N, 3.17%.

*3-((1R,5S,9R)-9-(Hydroxymethyl)-2-phenethyl-2-azabicyclo[3.3.1]nonan-5-yl)phenol* (**11**). A solution of **9** (165 mg, 0.451 mmol, 1.0 equiv.) was dissolved in dry dichloromethane (7 mL) and brought to −78 °C under an atmosphere of argon. To this cooled solution was added BBr_3_ (452 mg, 171 µL, 1.806 mmol, 4.0 equiv.) dropwise and the solution was stirred and allowed to warm to room temperature under argon for 16 h. The solution was cooled to 0 °C and methanol (2 mL) was added dropwise and stirred for 30 min. A solution of 1N HCl (4 mL) was added and the mixture was brought to 100 °C in a distillation apparatus. After 1 h, the solution was cooled to 0 °C, made basic (pH >10.5) with NH_4_OH, and then extracted with 9:1 CHCl_3_:MeOH (30 mL × 2). The organic extracts were washed with brine, dried over MgSO_4_, and concentrated. The crude residue was purified via flash chromatography eluting with 20–100% EtOAc in *n*-hexane to give **11** as a colorless oil (116 mg, 73%) [α]_D_^25^ + 24.5° (*c* 0.27, CHCl_3_). The free base was crystallized as the hydrobromide salt from isopropanol and diethyl ether by adding 48% HBr, mp 257–259 °C. ^1^H-NMR (400 MHz; CDCl_3_): *δ* 7.28–7.07 (m, 7H), 6.82 (d, *J* = 7.7 Hz, 1H), 6.71 (d, *J* = 7.9 Hz, 1H), 3.57–3.50 (m, 3H), 3.09–3.02 (m, 2H), 2.91–2.77 (m, 4H), 2.29–2.21 (m, 1H), 2.01–1.85 (m, 4H), 1.75–1.64 (m, 2H), 1.38–1.15 (m, 2H). ^13^C-NMR (100 MHz; CDCl_3_): *δ* 156.9, 150.0, 139.3, 129.5, 128.7, 128.5, 126.3, 116.7, 114.1, 112.5, 59.8, 57.6, 52.9, 49.9, 46.7, 41.1, 36.8, 33.1, 29.0, 21.5, 17.5; HRMS-ESI (*m*/*z*): [M +H]^+^ calcd for C_23_H_29_NO_2_ 352.2277; found: 352.2275. HBr salt: mp: 257–259 °C; anal. calcd for C_23_H_30_BrNO_2_·0.1 H2O: C, 63.46%; H, 6.90%; N, 3.20%. Found C, 63.57%; H, 7.01%; N, 3.22%.

*(1S,5S,E&Z)-9-(Methoxymethylene)-5-(3-methoxyphenyl)-2-phenethyl-2-azabicyclo [3.3.1]nonane* (**12***).* See synthesis of compound **7**. ^1^H NMR (400 MHz; CDCl_3_): δ 7.32–7.17 (m, 6H), 7.02 (d, *J =* 7.9 Hz, 1H), 6.97 (s, 1H), 6.76 (d, *J* = 8.3 Hz, 1H), 5.85 (s, 1H), 5.20 (s, 1H), 4.07 (bs, 1H), 3.82 (s, 3H), 3.41 (s, 3H), 3.08 (dt, *J =* 11.5, 5.9 Hz, 1H), 2.92–2.75 (m, 5H), 2.34 (dt, *J* = 13.5, 6.7 Hz, 1H), 2.20–1.92 (m, 5H), 1.75–1.68 (m, 1H), 1.54–1.42 (m, 1H), ^13^C NMR (101 MHz; CDCl_3_): δ 159.1, 149.5, 141.6, 140.8, 128.8, 128.6, 128.3, 125.9, 123.2, 119.9, 113.7, 110.6, 59.5, 58.8, 55.2, 52.1, 49.0, 40.6, 39.0, 38.2, 34.5, 29.7, 20.6. HRMS-ESI (*m*/*z*): [M +H]^+^ calcd for C_25_H_32_NO_2_ 378.2433, found 378.2432.

*((1S,5R,9R & 1S,5R,9S)-5-(3-Methoxyphenyl)-2-phenethyl-2-azabicyclo[3.3.1]nonan-9-yl)methanol* (**13** & **14**). A 25 mL single-neck round-bottom flask was charged with 4N aq HCl (7.3 mL). A solution of enol ether **12** (0.276 g, 0.73 mmol) in THF (7.3 mL) was added dropwise to the flask and stirred under argon at room temperature for 18 h. TLC analysis revealed complete consumption of the enol ethers. The reaction was cooled to 0 °C in an ice bath and charged with NaCNBH_3_ (0.069 g, 0.1 mmol). TLC analysis revealed complete consumption of the intermediate aldehydes after 2 h. The reaction was quenched with MeOH (5 mL) and stirred for 10 min. The bulk of the solvent was removed in vacuo and the residue was taken up in CHCl_3_ (10 mL) and H_2_O (10 mL). The aqueous phase was made alkaline with concentrated aq NH_4_OH (1 mL) and extracted with CHCl_3_ (3 × 10 mL). The combined organic layers were dried over MgSO_4_, filtered, and concentrated in vacuo. The resulting residue was purified via flash chromatography eluting with EtOAc/hexanes (0 to 100%) to afford 9-hydroxymethyl-5-phenylmorphans **13**:**14** as a 1:1.5 mixture of epimers (0.183g, 0.52 mmol, 69%).

*For 1S,5R,9R-***13***:* ^1^H NMR (400 MHz; CDCl_3_): δ 7.31–7.16 (m, 6H), 6.91 (d, *J* = 7.9 Hz, 1 H), 6.87 (s, 1H), 6.71 (d, *J* = 8.1 Hz, 1H), 3.79 (s, 3H), 3.64 (dd, *J* = 11.3, 3.4 Hz, 1H), 3.56 (dd, *J* = 11.5, 2.6 Hz, 1H), 3.31 (s, 1H), 3.13 (d, *J* = 10.1 Hz, 2H), 2.92–2.67 (m, 5H), 2.30 (dd, *J* = 14.6, 4.7 Hz, 1H), 2.06 (s, 1H), 2.00–1.81 (m, 2H), 1.77–1.65 (m, 2H), 1.59–1.47 (m, 1H); ^13^C NMR (101 MHz; CDCl_3_): δ 159.5, 150.8, 140.1, 129.1, 128.6, 128.4, 118.0, 112.4, 110.1, 64.4, 56.9, 55.2, 48.8, 45.0, 42.9, 38.3, 34.4, 31.2, 25.2, 23.1. HRMS-ESI (*m*/*z*): [M +H]^+^ calcd For C_24_H_32_NO_2_ 366.2433, found 366.2437.

*For 1S,5R,9S-***14***:* ^1^H NMR (400 MHz; CDCl_3_): δ 7.33–7.18 (m, 6H), 7.04 (d, *J* = 8.0 Hz, 1H), 6.98 (s, 1H), 6.74 (d, *J* = 7.8 Hz, 1H), 3.81 (s, 3H), 3.53 (t, *J* = 10.5 Hz, 1H), 3.42 (dd, *J* = 11.1, 4.4 Hz, 1H), 3.31 (s, 1H), 3.05 (dd, *J* = 9.2, 2.8 Hz, 1H), 2.95–2.79 (m, 4H), 2.53 (bs, 1H), 2.32–2.21 (m, 1H), 2.01–1.93 (m, 4H), 1.84–1.71 (m, 2H), 1.59–1.47 (m, 1H). ^13^C NMR (101 MHz; CDCl_3_): δ 159.6, 151.0, 140.6, 129.3, 128.8, 128.4, 126.0, 118.0, 112.1, 110.5, 61.2, 58.2, 55.2, 52.9, 49.7, 48.4, 41.4, 37.3, 34.6, 29.5, 21.7, 18.7; HRMS-ESI (*m*/*z*): [M +H]+ calcd for C_24_H_32_NO_2_ 366.2433, found 366.2434.

*3-((1S,5R,9R)-9-(Hydroxymethyl)-2-phenethyl-2-azabicyclo[3.3.1]nonan-5-yl)phenol* (**15**). A 10 mL flame-dried round-bottom flask was charged with phenylmorphan **13** (0.099 g, 0.27 mmol) and DCM (2.7 mL). The flask was cooled to −78 °C and charged with BBr_3_ (0.077 mL, 0.81 mmol) dropwise over 5 min. The reaction was allowed to warm gradually to room temperature over the course of 4 h at which time all the starting material was consumed as determined by TLC. The reaction was cooled to 0 °C and quenched by the dropwise addition of MeOH (2 mL). The crude reaction mixture was transferred to a separatory funnel and portioned between water (10 mL) and CHCl_3_ (10 mL). The aqueous layer was made basic by addition of saturated aq NH_4_OH and extracted with 9:1 CHCl_3_:MeOH (3 × 10 mL). The combined organic extracts were dried over MgSO_4_, filtered, and concentrated in vacuo. The resulting residue was purified by column chromatography eluting with EtOAc/hexanes (0 to 100%) to afford **15** as a white foam (0.076 g, 0.22 mmol, 81%): ^1^H-NMR (400 MHz; CDCl_3_): δ 7.32–7.28 (m, 2H), 7.22–7.20 (m, 3H), 7.15 (t, *J* = 8.1 Hz, 1H), 6.82 (m, 2H), 6.65 (d, *J* = 8.0 Hz, 1H), 3.64 (dd, *J* = 11.3, 3.5 Hz, 1H), 3.55 (dd, *J* = 11.1, 2.4 Hz, 1H), 3.32 (bs, 1H), 3.17–3.13 (m, 2H), 2.91–2.78 (m, 4H), 2.71 (q, *J* = 11.6 Hz, 1H), 2.31 (d, *J* = 15.3 Hz, 1H), 2.05–1.88 (m, 5H), 1.78–1.69 (m, 3H); ^13^C NMR (101 MHz; CDCl_3_): δ 156.4, 150.5, 139.9, 129.3, 128.6, 128.5, 126.2, 117.2, 113.0, 112.7, 64.4, 57.1, 56.8, 48.8, 45.1, 42.4, 38.1, 34.3, 31.2, 25.1, 23.0; HRMS-ESI (*m*/*z*): [M +H]+ calcd for C_23_H_30_NO_2_ 352.2277, found 352.2275. The free base was converted to its HBr salt for analysis and optical rotation, mp 237–240 °C, [α]_D_^25^ +37.8° (*c* 0.27, MeOH). Anal. calcd for C_25_H_30_BrNO_2_·0.3 H_2_O C, 63.10%; H, 7.04; N, 3.20%. Found C, 63.24%; H, 7.06%; N, 3.10%.

*3-((1S,5R,9S)-9-(Hydroxymethyl)-2-phenethyl-2-azabicyclo [3.3.1]nonan-5-yl)phenol* (**16**). See procedure for synthesis of **15**, phenylmorphan **16** was isolated a white foam (0.045 g, 0.13 mmol, 71%). ^1^H-NMR (400 MHz; CDCl_3_): δ 7.23 (d, *J* = 7.3 Hz, 2H), 7.15 (m, 2H), 7.06 (d, *J* = 7.2 Hz, 2H), 7.00 (s, 1H), 6.82 (d, *J* = 7.8 Hz, 1H), 6.68 (d, *J* = 8.0 Hz, 1H), 3.56–3.48 (m, 3H), 3.04–2.95 (m, 2H), 2.89–2.66 (m, 5H), 2.15 (q, *J* = 11.5 Hz, 1H), 2.05–1.75 (m, 5H), 1.72–1.59 (m, 1H), 1.60–1.49 (m, 1H); ^13^C NMR (101 MHz; CDCl_3_): δ 157.0, 150.5, 139.9, 129.5, 128.7, 128.4, 126.1, 116.9, 114.5, 113.4, 60.1, 57.7, 52.1, 50.1, 47.4, 41.3, 37.0, 33.5, 29.1, 21.7, 17.6; HRMS-ESI (*m*/*z*): [M +H]+ calcd for C_23_H_30_NO_2_ 352.2277, found 352.2276. The free base was converted to its HBr salt for analysis. Anal. calcd for C_25_H_30_BrNO_2_·0.2 H_2_O C, 63.36%; H, 7.03; N, 3.21%. Found C, 63.31%; H, 7.02%; N, 3.09%.

*tert-Butyl (1S,5S)-5-(3-methoxyphenyl)-9-oxo-2-azabicyclo[3.3.1]nonane-2-carboxylate* (**17**). To a solution of the 1*S*,5*S*-relative of **5**, (1*S*,5*S*)-5-(3-methoxyphenyl)-2-azabicyclo[3.3.1]nonan-9-one (4.6 g, 18 mmol) in acetonitrile (27 mL) was added potassium carbonate (5.4 g, 2.2 equiv, 39 mmol). The flask was purged with argon and cyanogen bromide (3.8 g, 7.1 mL, 5.0 molar, 2.0 equiv, 35 mmol) was added dropwise. The mixture was stirred at room temperature for 1.5 h, then brought to reflux for 1.5 h. At this point TLC showed consumption of starting material. Methanol (3.0 mL) and 2N HCl (37 mL) were added, and the solution was brought to reflux. The solution was left to stir at reflux overnight. After 18 h the solution was cooled to 0 °C and 7 M NH_4_OH in MeOH was added until pH 11. The aqueous mixture was extracted with CHCl_3_ and concentrated in vacuo. The crude mixture was dissolved in dry dichloromethane (20 mL) at 0 °C and to this was added di-tert-butyl dicarbonate (3.9 g, 1.1 equiv, 19 mmol), *N*,*N*-dimethylpyridin-4-amine (430 mg, 0.2 equiv, 3.5 mmol), and triethylamine (1.8 g, 2.5 mL, 1.1 equiv, 18 mmol) dropwise. The solution was stirred under argon. After 1 h, TLC showed consumption of starting material. Saturated ammonium chloride was added, and the mixture was extracted with dichloromethane (30 mL × 2), washed with brine, and dried over sodium sulfate. The crude mixture was loaded onto silica and purified via flash chromatography eluting with 0–30% ethyl acetate in hexane to yield **17** as a yellow oil (3.39 g, 55%) [α]_D_^25^ −33.8° (*c* 1.4, CHCl_3_). ^1^H-NMR (400 MHz; CDCl_3_): *δ* 7.30–7.26 (m, 1H), 6.81–6.77 (m, 3H), 4.39–4.09 (m, 2H), 3.80 (s, 3H), 3.21–3.14 (m, 1H), 2.65–2.48 (m, 2H), 2.39–2.27 (m, 2H), 2.23–2.14 (m, 2H), 1.80–1.64 (m, 2H), 1.49 (s, 9H); ^13^C-NMR (100 MHz; CDCl_3_): δ 159.6, 155.0, 145.9, 129.4, 119.7, 113.9, 111.7, 80.7, 64.0, 55.5, 53.2, 41.3, 41.0, 38.5, 35.9, 28.7, 17.9; HRMS-ESI (*m*/*z*): [M +H]^+^ calcd for C20H27NO4Na 368.1838; found: 368.1833.

*tert-Butyl (1S,5S,E)-9-(2-ethoxy-2-oxoethylidene)-5-(3-methoxyphenyl)-2-azabicyclo[3.3.1]nonane-2-carboxylate* (**18**). To dry tetrahydrofuran (10 mL) was added sodium hydride (275 mg, 60% weight, 3.0 equiv, 6.87 mmol), followed by slow addition of ethyl 2-(diethoxyphosphoryl)acetate (1.540 g, 1.36 mL, 3.0 equiv., 6.87 mmol). After 15 min, tert-butyl **17** (791 mg, 1 equiv., 2.29 mmol) was added dropwise as a solution in tetrahydrofuran (10 mL). The mixture was brought to reflux under an argon atmosphere. After 16 h, the mixture was cooled to 0 °C and ethanol (3 mL) was added. After stirring for 15 min, silica (3 g) was added directly to the mixture and then concentrated in vacuo. The mixture was purified via flash chromatography eluting with 0–45% ethyl acetate in hexane to give **18** as a colorless foam (834 mg, 88%) ^1^H-NMR (400 MHz; CDCl_3_): *δ* 7.32–7.25 (m, 1H), 6.90–6.78 (m, 3H), 6.01–5.95 (m,1H), 5.11 (s, 1H), 4.12–4.07 (m, 4H), 3.81 (s, 3H), 3.22–3.14 (m, 1H), 2.34–2.26 (m, 3H), 2.07 (d, *J* = 11.8 Hz, 3H), 1.71–1.68 (m, 1H), 1.51 (s, 9H), 1.22 (t, *J* = 7.1 Hz, 3H); ^13^C-NMR (100 MHz; CDCl_3_): δ 166.0, 164.5, 159.4, 155.5, 149.00, 148.95, 129.2, 119.91, 119.90, 119.88, 116.1, 113.9, 111.0, 79.7, 60.0, 55.2, 51.3, 45.7, 40.32, 40.26, 38.5, 33.8, 28.5, 18.1, 14.2; HRMS-ESI (*m*/*z*): [M +H]^+^ calcd for C_24_H_33_NO_5_ 416.2437; found: 416.2445.

*Ethyl 2-((1S,5R,9R & 1S,5R,9S)-5-(3-methoxyphenyl)-2-phenethyl-2-azabicyclo[3.3.1]nonan-9-yl)acetate* (**19** and **20**). Compound **18** (185 mg, 1 equiv, 443 µmol) was dissolved in isopropyl acetate (50 mL) and isopropanol (5 mL) in a 100 mL pear shaped flask. The vessel was attached to a Thales-Nano H-Cube Pro flow reactor. The solution was put through the reactor at a temperature of 80 °C, a pressure of 45 psi, and a flow rate of 0.4 mL/min. The reaction was monitored by ^1^H NMR to determine the consumption of starting material. The resulting solution was concentrated and redissolved in dichloromethane (5 mL) and brought to 0 °C. To this cooled solution was added 2,2,2-trifluoroacetic acid (505 mg, 339 µL, 10 equiv, 4.43 mmol) dropwise. After 15 min, the reaction was allowed to warm to room temperature. After 1 h, TLC showed consumption of starting material. Saturated NaHCO_3_ (15 mL) was added to quench the reaction and the solution was extracted with dichloromethane (15 mL × 3). The organic fractions were washed with brine, dried over MgSO_4_, and concentrated. The crude residue was dissolved in dry acetonitrile (20 mL) and K_2_CO_3_ (122 mg, 886 µmol, 2.0 equiv.) was added, followed by (2-bromoethyl)benzene (98.4 mg, 71.9 µL, 532 µmol, 1.2 equiv.). This mixture was brought to reflux and stirred for 16 h. The solution was then cooled to room temperature, filtered through celite, concentrated, and purified via flash chromatography eluting with 3–50% ethyl acetate in hexane to give the diastereomers **19** (96 mg, 51%) and **20** (21 mg, 11%).

*For* **19**-9*R:* [α]_D_^25^ +5.64° (*c* 3.0, CHCl_3_). ^1^H-NMR (400 MHz; CDCl_3_): *δ* 7.27–7.16 (m, 6H), 6.93–6.88 (m, 2H), 6.72–6.70 (m, 1H), 4.02 (q, *J* = 7.1 Hz, 2H), 3.78 (s, 3H), 3.13–2.96 (m, 3H), 2.86–2.62 (m, 6H), 2.29–2.25 (m, 1H), 2.14–2.07 (m, 1H), 2.00 (dd, *J* = 13.4, 4.8 Hz, 1H), 1.92–1.82 (m, 3H), 1.75 (dd, *J* = 12.6, 5.0 Hz, 1H), 1.70–1.66 (m, 1H), 1.60–1.54 (m, 1H), 1.18 (t, *J* = 7.1 Hz, 3H); ^13^C-NMR (100 MHz; CDCl_3_): *δ* 174.3, 159.6, 151.6, 140.9, 129.2, 128.7, 128.2, 125.7, 117.9, 111.8, 110.5, 59.9, 56.7, 55.1, 54.5, 49.0, 42.6, 41.8, 38.7, 34.4, 32.7, 30.1, 25.7, 23.4, 14.2; HRMS-ESI (*m*/*z*): [M +H]^+^ calcd for C_24_H_31_NO_2_: 422.2695; found: 422.2698.

*For* **20**-9*S*: [α]_D_^20^ +1.84° (*c* 2.4, CHCl_3_). ^1^H-NMR (400 MHz; CDCl_3_): *δ* ^1^H-NMR (400 MHz; CDCl_3_): δ 7.32–7.18 (m, 6H), 7.03–6.98 (m, 2H), 6.75–6.73 (m, 1H), 4.05 (t, *J* = 7.1 Hz, 2H), 3.80 (s, 3H), 3.05–3.01 (d, 3H), 2.90–2.80 (m, 5H), 2.29–2.23 (m, 2H), 2.09–1.94 (m, 5H), 1.81–1.78 (m, 2H), 1.58–1.56 (m, 1H), 1.19 (t, *J* = 7.1 Hz, 3H); 13C-NMR (100 MHz; CDCl3): *δ*13-C NMR (101 MHz; CDCl_3_): δ ^13^C-NMR (100 MHz; CDCl3): δ 173.0, 159.6, 150.9, 140.6, 129.2, 128.7, 128.3, 125.9, 118.1, 111.9, 110.9, 60.2, 58.0, 55.1, 54.4, 49.7, 41.9, 41.2, 38.2, 34.5, 33.4, 29.7, 28.9, 21.6, 18.6, 14.2; HRMS-ESI (*m*/*z*): [M +H]^+^ calcd for C_24_H_31_NO_2_: 422.2695; found: 422.2699.

*3-((1S,5R,9R)-9-(2-Hydroxyethyl)-2-phenethyl-2-azabicyclo[3.3.1]nonan-5-yl)phenol* (**21**). A flame-dried flask was charged with lithium aluminum hydride (55.7 mg, 95% weight, 3.0 equiv, 1.20 mmol) and brought to 0 °C under an argon atmosphere. To this flask was added dry tetrahydrofuran (1.5 mL). After 5 min, **19** (196 mg, 1 equiv, 401 µmol) was added dropwise as a solution in dry tetrahydrofuran (1.0 mL × 2). After 20 min, the ice bath was removed. Reaction was complete by TLC after 1 h. The mixture was cooled to 0 °C and water (400 µL) added to quench the reaction. After 10 min, sodium sulfate (500 mg) was added directly to the solution and the mixture was stirred for 10 min. The solution was filtered through celite and the filter was washed with dichloromethane (10 mL × 3). The filtrate was stripped of solvent in vacuo and used without purification in the next reaction. The crude reaction mixture was transferred in dry dichloromethane (3 mL) to a flame-dried round-bottom flask and the mixture was cooled to −78 °C. Tribromoborane (232 mg, 88 µL, 2 equiv, 0.93 mmol) was added dropwise and the reaction was stirred for 20 min. The cold bath was then removed, and the reaction continued to stir for 1.5 h at room temperature. At this point a small aliquot was removed and extracted with an ammonium hydroxide solution buffered to pH 9.5 with sodium bicarbonate. TLC of this mixture indicated complete consumption of starting material. The reaction mixture was cooled to 0 °C and quenched with 3 mL of methanol dropwise and stirred for 20 min. Then, 2N HCl (4 mL) was added, and a short-path distillation apparatus was fitted to the flask and distilled at 100 °C for 1 h. The resulting aqueous mixture was then cooled to 0 °C and made basic (~9.5) with NH_4_OH and extracted with 9:1 CHCl_3_:MeOH (15 mL × 3). The combined organic layers were washed with water and brine, dried with sodium sulfate, and concentrated. The crude mixture was purified with flash chromatography eluting with 5–45% ethyl acetate in hexanes to give **21** as a colorless foam (74 mg, 44% over two steps). [α]_D_^20^ +33.8° (*c* 1.1, CHCl_3_). The free base was crystallized as the hydrobromide salt from isopropanol by adding 48% HBr, mp 215–217 °C. HBr salt ^1^H NMR (400 MHz; CD_3_OD): δ 7.37–7.26 (m, 5H), 7.16 (t, *J* = 8.0 Hz, 1H), 6.83–6.77 (m, 2H), 6.65 (dd, *J* = 8.0, 2.0 Hz, 1H), 4.08 (s, 1H), 3.63 (tdd, *J* = 16.6, 9.2, 4.0 Hz, 4H), 3.53–3.39 (m, 2H), 3.14–3.07 (m, 2H), 2.61–2.40 (m, 3H), 2.24 (d, *J* = 14.7 Hz, 1H), 2.10–2.02 (m, 3H), 1.86–1.84 (m, 2H), 1.71–1.65 (m, 1H), 1.48–1.43 (m, 1H); ^13^C NMR (101 MHz; CD_3_OD): δ 158.8, 150.0, 137.7, 130.7, 130.0, 129.91, 129.87, 128.3, 117.4, 114.2, 113.4, 61.0, 57.7, 56.8, 51.3, 43.3, 42.2, 38.6, 31.6, 29.2, 28.8, 24.8, 21.9; HRMS-ESI (*m*/*z*): [M +H]^+^ calcd for C_24_H_31_NO_2_ 366.2433; found: 366.2430. HBr salt: mp: 215–217 °C; anal. calcd for C_24_H_32_BrNO_2_·0.3 H2O: C, 63.74%; H, 6.97%; N, 2.81%. Found C, 63.93%; H, 7.26%; N, 3.11%.

*3-((1S,5R,9S)-9-(2-Hydroxyethyl)-2-phenethyl-2-azabicyclo[3.3.1]nonan-5-yl)phenol* (**22**). A flame-dried flask was charged with lithium aluminum hydride (82.4 mg, 95% weight, 2.06 mmol) and brought to 0 °C under an argon atmosphere. To this flask was added dry tetrahydrofuran (1.5 mL). After 5 min, **20** (290 mg, 1 equiv, 688 µmol) was added dropwise as a solution in dry tetrahydrofuran (1.2 mL × 2). After 20 min, the ice bath was removed. Reaction was complete by TLC after 1 h. The mixture was cooled to 0 °C and water (400 µL) added to quench the reaction. After 10 min, sodium sulfate (500 mg) was added directly to the solution and stirred for 10 min. The solution was filtered through celite and the filter was washed with dichloromethane (10 mL × 3). The filtrate was stripped of solvent in vacuo and used without purification in the next reaction. The crude reaction mixture was transferred in dry dichloromethane (3 mL) to a flame-dried round-bottom flask and the mixture was cooled to −78 °C. Tribromoborane (226 mg, 85.5 µL, 0.90 mmol) was added dropwise and the reaction was stirred for 20 min. The cold bath was then removed, and the reaction continued to stir for 1.5 h at room temperature. At this point a small aliquot was removed and extracted with an ammonium hydroxide solution buffered to pH 9.5 with sodium bicarbonate. TLC of this mixture indicated complete consumption of starting material. The reaction mixture was cooled to 0 °C and quenched with 3 mL of methanol dropwise and stirred for 20 min. Then, 2N HCl (4 mL) was added, and a short-path distillation apparatus was fitted to the flask and distilled at 100 °C for 1 h. The resulting aqueous mixture was then cooled to 0 °C and made basic (~9.5) with NH_4_OH and extracted with 9:1 CHCl_3_:MeOH (15 mL × 3). The combined organic layers were washed with water and brine, dried with sodium sulfate, and concentrated. The crude mixture was purified with flash chromatography eluting with 5–65% ethyl acetate in hexanes to give **22** as a colorless foam (140 mg, 64% over two steps). [α]_D_^20^ −35.1° (*c* 1.0, CHCl_3_). The free base was crystallized as the hydrobromide salt from isopropanol by adding a solution of 48% HBr in water, mp 257–259 °C. ^1^H-NMR (400 MHz; CDCl_3_): δ 7.30–7.14 (m, 7H), 6.95 (s, 1H), 6.88 (d, *J* = 7.7 Hz, 1H), 6.68 (dd, *J* = 8.0, 1.8 Hz, 1H), 3.51–3.38 (m, 2H), 3.12–2.97 (m, 3H), 2.86 (t, *J* = 10.6 Hz, 4H), 2.51 (d, *J* = 10.2 Hz, 1H), 2.32–2.23 (m, 1H), 2.01–1.89 (m, 4H), 1.81–1.76 (m, 2H), 1.60–1.41 (m, 2H), 1.35–1.29 (m, 1H); ^13^C NMR (101 MHz; CDCl_3_): δ 156.8, 151.4, 140.2, 129.2, 128.7, 128.4, 126.1, 117.3, 113.7, 113.4, 60.8, 58.5, 55.4, 49.6, 41.0, 40.7, 38.2, 34.1, 30.0, 28.9, 21.7, 18.3; HRMS-ESI (*m*/*z*): [M +H]^+^ calcd for C_24_H_31_NO_2_ 366.2433; found: 366.2434. HBr salt: mp: 257–259 °C; anal. calcd for C_24_H_32_BrNO_2_: C, 64.42%; H, 6.92%; N, 2.85%. Found C, 64.57%; H, 7.22%; N, 3.14%.

*tert-Butyl (1R,5R)-5-(4-methoxyphenyl)-9-oxo-2-azabicyclo[3.3.1]nonane-2-carboxylate* (**23**). To a cooled (0 °C) solution of **5** (1.0 g, 4.08 mmol) in dry dichloromethane (50 mL) in a 100 mL round-bottom flask was added di-tert-butyl decarbonate (1.03 mL, 1.1 equiv., 4.49 mmol), *N*,*N*-dimethylpyridin-4-amine (10 mg, cat.), and triethylamine (0.63 mL, 1.1 equiv, 4.49 mmol) dropwise. The solution was stirred under argon. After 2 h, TLC showed consumption of starting material. Saturated ammonium chloride was added, and the mixture was extracted with dichloromethane (30 mL × 3), washed with brine, and dried over sodium sulfate. The crude product was purified via flash chromatography (EtOAc in hexanes, gradient 0–20%) to yield **23** as a yellow oil (1.10 g, 78%). Spectroscopic data matched enantiomer **17**.

*tert-Butyl (1R,5R,Z)-9-(2-ethoxy-2-oxoethylidene)-5-(4-methoxyphenyl)-2-azabicyclo[3.3.1]nonane-2-carboxylate* (**24**). To dry tetrahydrofuran (10 mL) was added sodium hydride (348 mg, 60% weight, 3.0 equiv, 8.7 mmol), followed by slow addition of ethyl 2-(diethoxyphosphoryl)acetate (1.70 mL, 3.0 equiv, 8.7 mmol). After 15 min, **23** (1.0 g, 1 equiv, 2.9 mmol) was added dropwise as a solution in tetrahydrofuran (10 mL). The mixture was brought to reflux under an argon atmosphere. After 16 h, the mixture was cooled to 0 °C and ethanol (3 mL) was added. After stirring for 15 min, the mixture was concentrated and loaded onto silica (3 g). The mixture was purified via flash chromatography (EtOAc in hexanes, gradient 0–20%) to give **24** as a white solid (1.14 g, 95%). Spectroscopic data matched enantiomer **18**.

*Ethyl 2-((1R,5S,9S & 1R,5S,9R)-5-(4-methoxyphenyl)-2-phenethyl-2-azabicyclo[3.3.1]nonan-9-yl)acetate* (**25** and **26**). (i) Compound **24** (1.0 g, 2.41 mmol) was dissolved in ethanol (20 mL) and transferred to a 250 mL pressure-tested reaction bottle. The vessel was charged with Escat 103 (5% Pd/C, 100 mg). The vessel was pressurized to 50 psi H_2_ in a Parr shaker at 60 °C for 1 h. The reaction mixture was filtered through celite and concentrated under vacuum to afford a yellow oil, used without further purification. (ii) The residue was dissolved in anhydrous dichloromethane (25 mL) and added to a 50 mL round-bottom flask, cooled to 0 °C, trifluoroacetic acid (1.83 mL, 24.0 mmol) was added slowly, and stirred at 0 °C for 1 h. The reaction was quenched with saturated aq NaHCO_3_, extracted with dichloromethane (3 × 25 mL), dried with Na_2_SO_4_, filtered, and concentrated in vacuo. The resultant yellow oil was used without further purification. (iii) The oil was dissolved in anhydrous acetonitrile (25 mL) and added to a 50 mL round-bottom flask. The flask was charged with K_2_CO_3_ (663 mg, 4.8 mmol) and 2-phenylethyl bromide (490 µL, 3.6 mmol) and heated to reflux for 18 h. The reaction was filtered through celite and concentrated in vacuo. The crude product was purified via flash chromatography (EtOAc in hexanes, gradient 0–50%), to afford **25** as a clear oil (600 mg, 60% yield) and **26** as a clear oil (200 mg, 20%).

*For* **25***:* [α]_D_^25^ −6.6° (*c* 1.4, CHCl_3_) ^1^H NMR (400 MHz; CDCl_3_) δ 7.27—7.14 (m, 6H), 6.92 (d, J = 7.9 Hz, 1H), 6.87 (t, J = 2.0 Hz, 1H), 6.70 (dd, J = 8.1, 2.4 Hz, 1H), 4.01 (q, J = 7.1 Hz, 2H), 3.81—3.76 (m, 3H), 3.08 (td, J = 12.0, 5.3 Hz, 1H), 3.00 (dd, J = 11.2, 7.9 Hz, 1H), 2.95 (d, J = 2.8 Hz, 1H), 2.62 (dd, J = 10.0, 1.5 Hz, 1H), 2.26 (dd, J = 14.8, 2.4 Hz, 1H), 2.16—2.07 (m, 1H), 2.00 (dd, J = 13.5, 5.3 Hz, 1H), 1.92—1.81 (m, 3H), 1.74 (dd, J = 13.0, 5.7 Hz, 1H), 1.68 (dd, J = 12.1, 4.9 Hz, 1H), 1.59—1.54 (m, 1H), 1.17 (t, J = 7.1 Hz, 3H). ^13^C-NMR (101 MHz, CDCl_3_): δ 174.3, 159.6, 151.5, 140.9, 129.2, 128.7, 128.1, 125.7, 117.9, 111.8, 110.5, 59.9, 56.7, 55.1, 54.4, 48.9, 42.6, 41.7, 38.7, 34.4, 32.7, 30.1, 25.7, 23.3, 14.2. HRMS-ESI (*m*/*z*): [M +H]^+^ calcd for C_27_H_36_NO_3_ 422.2695; found: 422.2695.

*For* **26**: [α]_D_^25^ −0.5° (*c* 1.4, CHCl_3_), ^1^H NMR (400 MHz; CDCl_3_) δ 7.30—7.26 (m, 2H), 7.24—7.19 (m, 5H), 7.02—7.00 (m, 1H), 6.96 (t, J = 2.1 Hz, 1H), 6.72 (dd, J = 8.1, 1.9 Hz, 1H), 4.03 (q, J = 7.1 Hz, 2H), 3.79 (s, 3H), 3.02 (dd, J = 9.0, 6.4 Hz, 3H), 2.86—2.80 (m, 5H), 2.24 (dd, J = 15.3, 11.3 Hz, 2H), 2.06 (dd, J = 15.4, 3.7 Hz, 1H), 2.02—1.92 (m, 4H), 1.78 (ddd, J = 14.0, 4.3, 2.7 Hz, 2H), 1.59—1.53 (m, 1H), 1.18 (t, J = 7.1 Hz, 3H). ^13^C-NMR (101 MHz, CDCl_3_): δ 173.0, 159.6, 150.9, 140.6, 129.2, 128.7, 128.3, 125.9, 118.1, 111.9, 110.9, 63.7, 60.2, 58.1, 55.2, 54.4, 49.7, 42.0, 41.2, 39.2, 38.2, 34.5, 33.4, 28.9, 21.6, 18.6, 14.2. HRMS-ESI (*m*/*z*): [M +H]^+^ calcd for C_27_H_36_NO_3_ 422.2695; found: 422.2695.

*Ethyl 2-((1R,5S,9S & 1R,5S,9R)-5-(4-hydroxyphenyl)-2-phenethyl-2-azabicyclo[3.3.1]nonan-9-yl)acetate* (**27** and **28**). A solution of **25** and **26** (200 mg, 0.475 mmol, 1.0 equiv) in dry dichloromethane (95 mL) was brought to −78 °C under an atmosphere of argon. To this cooled solution was added BBr_3_ (224 µL, 2.38 mmol, 5.0 equiv) dropwise and the solution was stirred and allowed to warm to room temperature under argon for 16 h. The solution was cooled to 0 °C and ethanol (3 mL) was added dropwise and stirred for 30 min. The solution was made basic (pH > 10.5) with NH_4_OH, and then extracted with CHCl_3_ (30 mL × 3). The organic extracts were washed with brine, dried over MgSO_4_, and solvent was removed in vacuo. The crude residue was purified via flash chromatography (50:45:5 CHCl_3_:MeOH:NH_4_OH in CHCl_3_, gradient 0–10%) (190 mg, 98%). Both epimers were used without further characterization.

*4-((1R,5S,9S)-9-(2-Hydroxyethyl)-2-phenethyl-2-azabicyclo[3.3.1]nonan-5-yl)phenol* (**29**). A solution of **27** (100 mg, 0.26 mmol) in dry tetrahydrofuran (5 mL) was cooled to 0 °C and lithium aluminum hydride (370 µL, 0.74 mmol, 3.0 equiv., 2.0M solution in THF) added dropwise. The reaction was warmed to room temperature and quenched with Na_2_SO_4_·10H_2_O, stirred for 15 min, and filtered through celite. The filtrate was concentrated in vacuo and purified via flash chromatography (50:45:5 CHCl_3_:MeOH:NH_4_OH, gradient 0–10%) to yield **29** as a white foam (62.4 mg, 66%). [α]_D_^25^ −36.1° (*c* 1.4, CHCl_3_), ^1^H NMR (400 MHz; CDCl_3_) δ 7.2–7.23 (m, 2H), 7.1–7.11 (m, 4H), 6.82 (d, J = 7.9 Hz, 1H), 6.79 (d, J = 2.0 Hz, 1H), 6.64 (dd, J = 7.9, 2.0 Hz, 1H), 3.5–3.44 (m, 2H), 3.18 (dd, J = 11.6, 8.2 Hz, 1H), 3.03 (dt, J = 12.5, 6.1 Hz, 2H), 2.8–2.72 (m, 4H), 2.45 (td, J = 12.5, 8.6 Hz, 1H), 2.2–2.21 (m, 2H), 1.96 (dd, J = 13.7, 5.3 Hz, 1H), 1.9–1.80 (m, 2H), 1.70 (ddt, J = 23.7, 13.9, 6.3 Hz, 3H), 1.5–1.46 (m, 1H), 1.4–1.35 (m, 1H). ^13^C-NMR (101 MHz, CDCl_3_): δ 156.3, 151.0, 140.1, 129.3, 128.8, 128.3, 126.0, 117.3, 112.8, 112.7, 60.6, 57.7, 55.2, 48.7, 43.6, 42.9, 38.6, 33.9, 31.2, 29.6, 25.9, 23.0. HRMS-ESI (*m*/*z*): [M +H]^+^ calcd for C_24_H_32_NO_2_ 366.2433; found: 366.2429. Anal. calcd for C_24_H_31_NO_2_·0.15 H_2_O·0.2 CHCl_3_: C, 78.86%; H, 8.55%; N, 3.83%. Found C, 74.14%; H, 8.08%; N, 3.52%.

*4-((1R,5S,9R)-9-(2-Hydroxyethyl)-2-phenethyl-2-azabicyclo[3.3.1]nonan-5-yl)phenol* (**30**). A solution of **28** (75 mg, 0.18 mmol) in dry tetrahydrofuran (5 mL) was cooled to 0 °C and lithium aluminum hydride (272 µL, 0.55 mmol, 2.0M solution in THF) added dropwise. The reaction was warmed to room temperature and quenched with Na_2_SO_4_·10H_2_O, stirred for 15 min, and filtered through celite. The filtrate was concentrated in vacuo and purified via flash chromatography (50:45:5 CHCl_3_:MeOH:NH_4_OH, gradient 0–10%) to yield **30** as a white foam (45 mg, 47%). [α]_D_^25^ +38.7° (*c* 1.4, CHCl_3_), ^1^H NMR (400 MHz; CDCl_3_) δ 7.26 (t, J = 7.4 Hz, 2H), 7.2–7.10 (m, 4H), 6.91 (s, 1H), 6.84 (d, J = 7.9 Hz, 1H), 6.67 (dd, J = 8.0, 1.7 Hz, 1H), 3.4–3.34 (m, 2H), 3.0–3.03 (m, 2H), 2.97 (td, J = 12.0, 4.9 Hz, 1H), 2.8–2.78 (m, 4H), 2.47 (d, J = 10.0 Hz, 1H), 2.24 (td, J = 12.9, 7.7 Hz, 1H), 1.9–1.86 (m, 4H), 1.7–1.70 (m, 2H), 1.55 (t, J = 9.6 Hz, 1H), 1.43 (td, J = 9.5, 5.1 Hz, 1H), 1.3–1.25 (m, 1H). ^13^C-NMR (101 MHz, CDCl_3_): δ 157.0, 151.2, 140.0, 129.2, 128.7, 128.4, 126.1, 117.1, 113.7, 113.7, 60.9, 58.3, 55.3, 49.6, 41.2, 40.7, 38.2, 33.9, 29.9, 28.8, 21.7, 18.0. HRMS-ESI (*m*/*z*): [M +H]^+^ calcd for C_24_H_32_NO_2_ 366.2433; found: 366.2430. Anal. calcd for C_24_H_31_NO_2_·0.5 H_2_O·0.25 CH_2_Cl_2_: C, 78.87%; H, 8.55%; N, 3.83%. Found C, 73.70%; H, 8.43%; N, 3.47%.

*Ethyl (E&Z)-3-((1R,5S,9R)-5-(3-methoxyphenyl)-2-phenethyl-2-azabicyclo[3.3.1]nonan-9-yl)acrylate* (**31** [9*R*] and **32** [9*S*]). In step 1, compound **7** (1.192 g, 3.157 mmol) was dissolved in THF (8 mL) and to this was added 6 N HCl (8 mL). After 4 h, TLC showed consumption of starting material. The solution was cooled to 0 °C and made basic (pH > 9.5) with 12 N NH_4_OH and extracted with dichloromethane (25 mL × 3) and the organic layers were washed with brine, filtered through sodium sulfate, and concentrated in a flame-dried flask. To another flame-dried flask was added NaH (328.4 mg, 60% weight, 2.6 equiv, 8.209 mmol) anhydrous THF (5 mL). To this solution was added ethyl 2-(diethoxyphosphoryl)acetate (2.189 g, 1.94 mL, 97% weight, 3.0 equiv, 9.472 mmol). The mixture was brought to 0 °C and the crude material from step 1 was added dropwise over 5 min in a solution in THF (2.5 mL × 2). After 30 min the ice bath was removed, and the reaction was left for 16 h. The solution was cooled to 0 °C and ethanol (5 mL) was added and stirred for 10 min to quench the reaction. Silica (20 g) was added directly to the mixture and solvents removed in vacuo. The material was loaded onto a column and purified via flash chromatography eluting with 5–65% ethyl acetate in hexanes. The C_9_*R* and C_9_*S* isomers were each separated as mixtures of E and Z (834 mg, 88%, 1.2:1 C_9_*R*:C_9_*S*).

*For* **31**-9*R:* ^1^H NMR (400 MHz; CDCl_3_): δ 7.40–7.19 (m, *J* = 18.9, 9.4 Hz, 6H), 6.95–6.88 (m, 2H), 6.69 (dd, *J* = 8.1, 2.0 Hz, 1H), 5.83 (d, *J* = 15.7 Hz, 1H), 4.08 (q, *J* = 7.1 Hz, 2H), 3.74 (s, 3H), 3.25–3.23 (m, 1H), 3.06–3.01 (m, 3H), 2.82 (q, *J* = 7.4 Hz, 4H), 2.14 (t, *J* = 5.8 Hz, 2H), 1.95 (d, *J* = 11.6 Hz, 2H), 1.82 (dd, *J* = 13.2, 3.6 Hz, 2H), 1.52–1.45 (m, 1H), 1.26–1.18 (m, 4H); ^13^C NMR (101 MHz; CDCl_3_): δ 166.3, 159.5, 150.6, 148.9, 140.4, 129.18, 129.0, 128.7, 128.4, 126.1, 123.1, 118.2, 112.3, 110.7, 60.2, 58.3, 57.8, 55.1, 49.6, 48.5, 41.1, 38.2, 34.7, 29.7, 22.0, 19.9, 14.2; HRMS-ESI (*m*/*z*): [M +H]^+^ calcd for C_28_H_36_NO_3_ 434.2695; found: 434.2691.

*For* **32**-9*S:* ^1^H NMR (400 MHz; CDCl_3_): δ 7.27–7.10 (m, 7H), 6.87–6.81 (m, 2H), 6.68 (dd, *J* = 8.1, 2.1 Hz, 1H), 5.77–5.67 (m, 1H), 4.08 (qd, *J* = 7.1, 2.1 Hz, 2H), 3.76 (s, 3H), 3.14–3.07 (m, 3H), 2.86–2.72 (m, 5H), 2.44–2.36 (m, 1H), 2.30–2.27 (m, 1H), 2.09–2.05 (m, 1H), 1.99–1.79 (m, 3H), 1.67 (t, *J* = 5.0 Hz, 1H), 1.53–1.44 (m, 1H), 1.21 (t, *J* = 7.1 Hz, 3H); ^13^C NMR (101 MHz; CDCl_3_): δ 166.7, 159.4, 151.2, 150.8, 140.9, 129.1, 128.8, 128.2, 125.8, 121.9, 118.2, 112.4, 110.3, 59.9, 57.9, 57.1, 55.1, 49.1, 49.01, 42.1, 38.7, 34.3, 30.4, 25.7, 23.1, 14.3; HRMS-ESI (*m*/*z*): [M +H]^+^ calcd for C_28_H_36_NO_3_ 434.2695; found: 434.2694.

*3-((1R,5S,9R)-5-(3-Methoxyphenyl)-2-phenethyl-2-azabicyclo[3.3.1]nonan-9-yl)propan-1-ol* (**33**). Compound **31** (**9*R***) (570 mg, 1 equiv., 1.31 mmol) was dissolved in a 10:1 mixture of ethanol:ethyl acetate (20 mL) and transferred to a Parr shaker vessel. Escat Pd/c (5%) (0.1 equiv) was added and the vessel was attached to a Parr shaker, charged with hydrogen up to 45 psi, and shaken overnight. The mixture was filtered through celite and sodium sulfate into a flame-dried flask. This flask was brought to 0 °C under an argon atmosphere and anhydrous tetrahydrofuran (1.5 mL) was added. After 5 min, lithium aluminum hydride (155 mg, 2.05 mL, 2.0 molar, 3.0 equiv, 4.10 mmol) was added dropwise as a solution in tetrahydrofuran. After 20 min, the ice bath was removed. TLC taken after 1 h showed complete reaction. The mixture was cooled to 0 °C and water (600 µL) added to quench the reaction which was then stirred for 10 min. Sodium sulfate (500 mg) was added directly to the mixture, and it was filtered through a pad of celite. The celite was washed with 10% MeOH in dichloromethane and the crude material was purified via flash chromatography eluting with 0.5–20% 50:45:5 CHCl_3_:MeOH:NH_4_OH to give **33** as a yellow foam (480 g, 89%). ^1^H NMR (400 MHz; CDCl_3_): δ 7.31–7.18 (m, 6H), 7.00–6.94 (m, 2H), 6.84–6.70 (m, 2H), 3.78 (s, 3H), 3.54–3.40 (m, 2H), 3.08–3.01 (m, 2H), 2.88–2.80 (m, 4H), 2.45–2.15 (m, 4H), 2.10–1.86 (m, 5H), 1.84–1.65 (m, 3H), 1.60–1.48 (m, 2H), 1.42–1.25 (m, 3H), 1.06–0.99 (m, 1H); ^13^C NMR (101 MHz; CDCl_3_): δ 159.5, 151.7, 140.4, 129.1, 128.7, 128.4, 126.1, 118.1, 112.2, 110.4, 62.7, 58.2, 55.1, 54.2, 49.9, 44.9, 41.3, 38.8, 34.4, 30.7, 28.8, 23.2, 21.8, 18.1; HRMS-ESI (*m*/*z*): [M +H]^+^ calcd for C_26_H_36_NO_2_ 394.2746; found: 394.2752.

*3-((1R,5S,9R)-9-(3-Hydroxypropyl)-2-phenethyl-2-azabicyclo[3.3.1]nonan-5-yl)phenol* (**34**). A solution of **33** (185 mg, 0.470 mmol, 1.0 equiv.) was dissolved in dry dichloromethane (3 mL) and brought to −78 °C under an atmosphere of argon. To this cooled solution was added BBr_3_ (177 mg, 67 µL, 0.705 mmol, 1.5 equiv.) dropwise and the solution was stirred and allowed to warm to room temperature under argon for 16 h. The solution was cooled to 0 °C and methanol (2 mL) was added dropwise and stirred for 30 min. A solution of 2N HCl (4 mL) was added, and the mixture was brought to 100 °C in a distillation apparatus for removal of the dichloromethane. After 1 h, the solution was cooled to 0 °C, made basic (pH 9.5) with 14 N NH_4_OH and sodium bicarbonate, and then extracted with 9:1 CHCl_3_:MeOH (30 mL × 2). The organic extracts were washed with brine, dried over MgSO_4_, and concentrated in vacuo. The crude residue was purified via flash chromatography eluting with 20–100% ethyl acetate in *n*-hexane to give **34** as a colorless oil (65 mg, 36%) [α]_D_^20^ +42.7° (*c* 0.9, CHCl_3_). The free base was crystallized as the hydrobromide salt from isopropanol and diethyl ether by adding a solution of 48% HBr, mp 218–220 °C. ^1^H NMR (400 MHz; CDCl_3_): δ 7.31–7.13 (m, 6H), 6.92–6.86 (m, 2H), 6.67–6.64 (m, 1H), 3.52–3.43 (m, 2H), 3.08–2.99 (m, 3H), 2.89–2.84 (m, 3H), 2.33–2.22 (m, 2H), 2.06–1.74 (m, 6H), 1.58–1.49 (m, 2H), 1.38–1.15 (m, 3H); ^13^C NMR (101 MHz; CDCl_3_): δ 156.6, 151.6, 140.2, 129.2, 128.7, 128.4, 126.1, 117.3, 113.3, 113.2, 62.2, 58.3, 54.6, 49.8, 43.9, 41.0, 38.6, 34.0, 30.0, 28.8, 22.9, 21.7, 18.0; HRMS-ESI (*m*/*z*): [M +H]^+^ calcd for C_25_H_34_NO_2_ 380.2590; found: 380.2589. C_25_H_34_BrNO_2_ 0.3 H_2_O: C, 64.46%; H, 7.49%; N, 3.01%. Found C, 64.50%; H, 7.49%; N, 3.02%.

*Ethyl 3-((1S,5R,9S)-5-(3-methoxyphenyl)-2-phenethyl-2-azabicyclo[3.3.1]nonan-9-yl)acrylate* (**37**). See the procedure for the synthesis of compounds **31** and **32**. ^1^H NMR (400 MHz; CDCl_3_): δ 7.32–7.19 (m, 6H), 6.95–6.88 (m, 3H), 6.70 (dd, *J* = 8.1, 2.4 Hz, 1H), 5.82 (dd, *J* = 15.8, 0.9 Hz, 1H), 4.10 (q, *J* = 7.2 Hz, 2H), 3.77 (s, 3H), 3.24 (dd, *J* = 8.2, 2.7 Hz, 1H), 3.12–2.99 (m, 3H), 2.83 (q, *J* = 7.5 Hz, 4H), 2.14 (t, *J* = 4.6 Hz, 3H), 2.01–1.94 (m, 2H), 1.86–1.79 (m, 2H), 1.53 (qt, *J* = 8.8, 4.3 Hz, 1H), 1.22 (t, *J* = 7.1 Hz, 3H); ^13^C NMR (101 MHz; CDCl_3_): δ 166.4, 159.5, 150.6, 148.9, 140.4, 129.1, 128.7, 128.4, 126.0, 123.1, 118.2, 112.2, 110.7, 60.2, 58.3, 57.7, 55.1, 49.6, 48.5, 41.1, 38.2, 34.7, 29.7, 22.0, 19.8, 14.2; HRMS-ESI (*m*/*z*): [M +H]^+^ calcd for C_28_H_36_NO_3_ 434.2695; found: 434.2693.

*Ethyl 3-((1S,5R,9S)-5-(3-hydroxyphenyl)-2-phenethyl-2-azabicyclo[3.3.1]nonan-9-yl)acrylate* (**38**). A solution of **37** (548 mg, 1.26 mmol, 1.0 equiv.) was dissolved in dry dichloromethane (12 mL) and brought to −78 °C under an atmosphere of argon. To this cooled solution was added BBr_3_ (633 mg, 239 µL, 2.53 mmol, 2.0 equiv.) dropwise and the solution was stirred and allowed to warm to room temperature under argon for 16 h. The solution was cooled to 0 °C and methanol (2 mL) was added dropwise and stirred for 30 min. A solution of 2N HCl (4 mL) was added, and the mixture was brought to 100 °C in a distillation apparatus. After 1 h, the solution was cooled to 0 °C, made basic (pH 9.5) with 14 N NH_4_OH and sodium bicarbonate, and then extracted with CHCl_3_ (30 mL × 2). The organic extracts were washed with brine, dried over MgSO_4_, and concentrated. The crude residue was purified via flash chromatography eluting with 5–55% ethyl acetate in *n*-hexane to give **38** as a yellow oil (310 mg, 59%). ^1^H NMR (400 MHz; CDCl_3_): δ 7.31–7.18 (m, 5H), 7.17–7.14 (m, 1H), 6.95–6.88 (m, 2H), 6.81–6.76 (m, 1H), 6.63 (dd, *J* = 7.9, 2.1 Hz, 1H), 5.81 (d, *J* = 15.8 Hz, 1H), 4.12–4.05 (m, 2H), 3.24–3.22 (m, 1H), 3.09–3.01 (m, 3H), 2.89–2.80 (m, 4H), 2.19–2.05 (m, 3H), 2.03–1.92 (m, 2H), 1.84–1.78 (m, 2H), 1.59–1.50 (m, 1H), 1.21 (t, *J* = 7.1 Hz, 3H); ^13^C NMR (101 MHz; CDCl_3_): δ 166.6, 156.0, 150.6, 148.9, 140.2, 129.4, 128.7, 128.4, 126.1, 123.1, 117.7, 113.4, 113.2, 60.3, 58.0, 57.7, 49.6, 48.1, 40.7, 38.0, 34.3, 29.6, 21.9, 19.6, 14.2; HRMS-ESI (*m*/*z*): [M +H]^+^ calcd for C_27_H_34_NO_3_ 420.2539; found: 420.2539.

*3-((1S,5R,9S)-9-(3-Hydroxypropyl)-2-phenethyl-2-azabicyclo[3.3.1]nonan-5-yl)phenol* (**39**). Phenolic ester **38** (300 mg, 1 equiv, 0715 mmol) was dissolved in a 10:1 mixture of ethanol:ethyl acetate (20 mL) and transferred to a Parr shaker vessel. Escat Pd/C (5%) (0.1 equiv) was added and the vessel was attached to a Parr shaker, charged with hydrogen up to 45 psi, and shaken overnight. The mixture was filtered through celite and sodium sulfate into a flame-dried flask. This flask was brought to 0 °C under an argon atmosphere and anhydrous tetrahydrofuran (1.5 mL) was added. After 5 min, lithium aluminum hydride (84.5 mg, 1.11 mL, 2.0 molar, 3.0 equiv, 2.23 mmol) was added dropwise as a solution in tetrahydrofuran. After 20 min, the ice bath was removed. TLC taken after 1 h showed complete reaction. The mixture was cooled to 0 °C and water (600 µL) was added to quench the reaction which was then stirred for 10 min. Sodium sulfate (500 mg) was added directly to the mixture and the mixture was filtered through a pad of celite. The celite was washed with 10% MeOH in dichloromethane and the crude material was purified via flash chromatography eluting with 0.5–20% 50:45:5 CHCl_3_:MeOH:NH_4_OH in CHCl_3_ to give **39** as a yellow foam (203 mg, 72%) [α]_D_^20^ −42.7° (*c* 0.9, CHCl_3_). The free base was crystallized as the hydrobromide salt from isopropanol and diethyl ether by adding a solution of 48% HBr, mp 218–220 °C. ^1^H NMR (400 MHz; CDCl_3_): δ 7.30–7.12 (m, 6H), 6.88–6.86 (m, 2H), 6.66–6.64 (m, 1H), 3.51–3.40 (m, 2H), 3.10–3.01 (m, 3H), 2.85 (q, *J* = 9.7 Hz, 4H), 2.32–2.19 (m, 2H), 2.05–1.85 (m, 4H), 1.81–1.71 (m, 2H), 1.57–1.48 (m, 2H), 1.37–1.14 (m, 3H); ^13^C NMR (101 MHz; CDCl_3_):δ 156.6, 151.6, 140.2, 129.2, 128.7, 128.4, 126.1, 117.2, 113.4, 113.2, 62.2, 58.3, 54.6, 49.8, 43.9, 41.0, 38.6, 34.0, 30.0, 28.8, 22.9, 21.7, 18.0; HRMS-ESI (*m*/*z*): [M +H]^+^ calcd for C_25_H_34_NO_2_ 380.2590; found: 380.2586. C_25_H_34_BrNO_2_: C, 65.21%; H, 7.44%; N, 3.04%. Found C, 65.36%; H, 7.58%; N, 3.14%.

*3-((1R,5R,E)-9-(Methoxymethylene)-2-phenethyl-2-azabicyclo[3.3.1]nonan-5-yl)phenol* (**40**). Ketone **6** (2.28 g, 1 equiv, 6.52 mmol) was dissolved in dry dichloromethane (30 mL) in a flame-dried round-bottom flask and the mixture was cooled to −78 °C. Tribromoborane (8.17 g, 3.10 mL, 5 equiv, 32.6 mmol) was added dropwise and the reaction was stirred for 30 min. The cold bath was then removed, and the reaction continued to stir for 30 min at room temperature. At this point a small aliquot was removed and extracted with an ammonium hydroxide solution buffered to pH 9.5 with sodium bicarbonate. TLC of this mixture indicated complete consumption of starting material. The reaction mixture was cooled to 0 °C and quenched with 3 mL of methanol dropwise and stirred for 20 min. Then, 2N HCl (20 mL) was added, and a short-path distillation apparatus was fitted to the flask and distilled at 100 °C for 1 h. The resulting aqueous mixture was then cooled to 0 °C and made basic (~9.5) with NH_4_OH and extracted with 9 CHCl_3_ (50 mL × 3). The combined organic layers were washed with water and brine, dried with sodium sulfate, and purified via flash chromatography, eluting with 10–40% ethyl acetate in hexane to give free phenol as a colorless oil (1.634 g, 75%). The free phenolic tertiary amine (1.634 g, 1 equiv, 4.871 mmol) from the reaction was dissolved in dry tetrahydrofuran (40 mL) and (methoxymethyl)triphenylphosphonium chloride (5.009 g, 14.61 mmol, 3.0 equiv) was added and the solution brought to 0 °C. LiHMDS (2.119 g, 12.66 mL, 2.6 equiv, 1.0 M solution in THF, 12.66 mmol) was added dropwise over 10 min. After 30 min the ice bath was removed, and the solution was stirred under argon for 16 h. The mixture was cooled to 0 °C and methanol (20 mL) was added and stirred for 10 min. The solvents were removed in vacuo and the residue was taken up in CHCl_3_ (50 mL) and washed with water which was made basic (pH 9–9.5) with NH_4_OH. The combined organic extracts were washed with brine, dried over MgSO_4_, concentrated in vacuo, and purified via flash chromatography eluting with 0.5–8% 50:45:5 CHCl_3_:MeOH:NH_4_OH to give **40** as a yellow oil (385 mg, 21.7%) [15].

*3-((1R,5S,9R)-9-(Methoxymethyl)-2-phenethyl-2-azabicyclo[3.3.1]nonan-5-yl)phenol* (**41**). Enol ether **40** (240 mg, 1 equiv., 660 umol) was dissolved in ethanol (20 mL) and transferred to a Parr shaker vessel. Escat Pd/c (10%) (0.2 equiv.) was added and the vessel was attached to a Parr shaker, charged with hydrogen up to 45 psi, and shaken overnight. The mixture was filtered through celite and purified via flash chromatography 0.5–10% 50:45:5 CHCl_3_:MeOH:NH_4_OH to give **41** and **42** as a colorless foam (113 mg, 46.8% and 20 mg, 8.3%). Then, **41** was further purified by crystallization as the hydrobromide salt from isopropanol. [α]_D_^20^ +34.5° (*c* 1.0, CHCl_3_). mp 250–252 °C. ^1^H NMR (400 MHz; CDCl_3_): δ 7.30–7.26 (m, 3H), 7.21–7.16 (m, 4H), 6.95 (d, *J* = 7.9 Hz, 1H), 6.88 (t, *J* = 1.9 Hz, 1H), 6.69 (dd, *J* = 7.9, 2.1 Hz, 1H), 3.38 (t, *J* = 10.5 Hz, 1H), 3.30 (s, 1H), 3.18 (s, 3H), 3.08–2.98 (m, 3H), 2.90–2.78 (m, 4H), 2.68–2.64 (m, 1H), 2.23 (dt, *J* = 13.7, 9.8 Hz, 1H), 1.97–1.77 (m, 6H), 1.74–1.69 (m, 1H), 1.61–1.52 (m, 1H). ^13^C NMR (101 MHz; CDCl_3_): δ156.1, 151.3, 140.6, 129.3, 128.7, 128.3, 125.9, 117.5, 113.2, 113.1, 70.5, 58.6, 58.2, 52.6, 49.6, 44.6, 41.5, 36.9, 34.3, 29.5, 21.7, 18.4. HRMS-ESI (*m*/*z*): [M +H]^+^ calcd for C_24_H_32_NO_2_ 366.2433; found: 366.2430. C_24_H_32_BrNO_2_. 0.2 H_2_O: C, 64.05%; H, 7.26%; N, 3.11%. Found C, 64.14%; H, 7.19%; N, 3.05%.

### 3.3. In Vitro Assay

#### 3.3.1. Cell Lines and Cell Culture

cAMP Hunter^TM^ Chinese hamster ovary cells (CHO-K1) expressing human μ-opioid receptor (OPRM1, catalog # 95-0107C2), human δ-receptor (OPRMD1, catalog # 95-0108C2), and human κ-opioid receptor (OPRMK1, catalog # 95-0088C2) and the PathHunter^TM^ Chinese hamster ovary cell line stably expressing the human µ-opioid receptor β-arrestin2 EFC (catalog # 93-0213C2) were purchased from Eurofins DiscoverX (Fremont, CA, USA). All cell lines were maintained in F-12 media with 10% fetal bovine serum (Life Technologies, Grand Island, NY, USA), 1% penicillin/streptomycin/L-glutamine (Life Technologies), and 800 µg/mL geneticin (Mirus Bio, Madison, WI, USA), except for the media for the PathHunter^TM^ cells that was supplemented with an additional 300 µg/mL hygromycin B (Mirus Bio). All cells were grown at 37 °C and 5% CO_2_ in a humidified incubator.

#### 3.3.2. Forskolin-Induced cAMP Accumulation Assays

Assays were performed as previously described [26]. Briefly, the cAMP Hunter cells were plated in a 384-well white tissue culture microplate at a 10,000 cells/well density and incubated overnight at 37 °C. Compounds were first dissolved in DMSO to form 5 mM stock solutions, and then 9–10 doses of 100X solutions were prepared by serial dilution with DMSO. Subsequently, these 100X solutions were further diluted with assay buffer consisting of Hanks’s buffered salt solution, HEPES, and forskolin to generate the 5× working solutions. In the agonist assay, cells were treated with compounds (at 1× final concentration) and incubated at 37 °C for 30 min. In the antagonist assay [22], cells were pretreated with compounds for 15 min at 37 °C followed by 30 min incubation at 37 °C with selected agonists at their EC50 or EC90 dose. The HitHunter cAMP Assay for Small Molecules by Eurofins DiscoverX (Fremont, CA, USA) was then used according to the manufacturer’s directions and the BioTek Synergy H1 hybrid and Cytation 5 plate readers (BioTek, Winooski, VT, USA) and Gen5 Software version 2.01 (BioTek, Winooski, VT, USA) were used to quantify luminescence.

#### 3.3.3. β-Arrestin2 EFC Recruitment Assay

Assays proceeded as previously described [23]. Briefly, the PathHunter CHO-K1 OPRM1 β-arrestin2 cell line was plated in 384-well white tissue culture microplates at 5000 cells/well and incubated overnight at 37 °C. Compounds were first dissolved in DMSO to form 5 mM stock solutions, and then 10 doses of 100× solutions were prepared by serial dilution with DMSO. Subsequently, these 100× solutions were further diluted with assay buffer consisting of Hanks’s buffered salt solution and HEPES to generate the 5× working solutions. Cells were treated with compounds (at 1× final concentration) and incubated at 37 °C for 90 min. The PathHunter Detection Kit by Eurofins DiscoverX (Fremont, CA, USA) was then used according to the manufacturer’s directions and the BioTek Synergy H1 hybrid and Cytation 5 plate readers (BioTek, Winooski, VT, USA) and Gen5 Software version 2.01 (BioTek, Winooski, VT, USA) were used to quantify luminescence. Data were blank subtracted with vehicle control followed by normalization to the maximum response of DAMGO and were then analyzed in GraphPad Prism 8 (GraphPad, LaJolla, CA, USA) using nonlinear regression. Bias factors were calculated using Equations (1)–(3) as previously described [27,28], where B is the test compound and A is the reference compound (DAMGO).

(1) $$∆\log\left(E_{max}/{EC}_{50}\right)=\log\left(E_{max}B/{EC}_{50}B\right)-\log\left(E_{max}A/{EC}_{50}A\right)$$

(2) $$∆∆\log\left(E_{max}/{EC}_{50}\right)=∆\log{\left(E_{max}/{EC}_{50}\right)}_{cAMP\;pathway}-∆\log{\left(E_{max}/{EC}_{50}\right)}_{\beta -arrestin-2pathway}$$

(3) $$bias\;factor=10^{∆∆log(E_{max}/{EC}_{50})}$$

### 3.4. In Vivo Activity

#### 3.4.1. General Information

Male squirrel monkeys (*Saimiri sciureus*) were housed in a climate-controlled vivarium with a 12 h light/dark cycle (7 a.m.–7 p.m.) in the McLean Hospital Animal Care Facility (licensed by the U.S. Department of Agriculture and compliant with guidelines provided by the Committee on Care and Use of Laboratory Animals of the Institute of Laboratory Animals Resources, Commission on Life Sciences, National Research Council; 2011). These experiments were approved under IACUC protocol #2015N000165.

#### 3.4.2. Warm-Water Squirrel Tail Withdrawal

Tail withdrawal latencies were assessed as described previously [18,25]. Briefly, monkeys were seated in customized Plexiglas chairs that allowed their tails to hang freely. Tail withdrawal latencies were measured by immersing the subject’s tail in water held at 35 °C or 52 °C (temperatures were presented in a randomized order during successive test components). After obtaining a baseline tail withdrawal latency, complete dose response curves were generated in each subject using standard cumulative dosing procedures. Briefly, every 15 min after an injection, tail withdrawal latencies at each temperature were redetermined and subjects were injected with the next dose, such that the total (cumulative) dose was increased by ½ log_10_ units in each successive cycle. This procedure was repeated until either (a) the tail withdrawal latency from 52 °C water reached the maximum allowable latency (10 s), or (b) tail withdrawal latency no longer increased with increases in dose of the test drug.

#### 3.4.3. Squirrel Monkey Ventilation

Ventilation measures were assessed as described previously [18]. Briefly, squirrel monkeys were acclimated to a customized acrylic chamber (10′′d × 10′′w × 10′′h) that served as a whole-body plethysmograph (EMKA Technologies, Montreal, PQ, Canada). Gas (either air or a 5% CO_2_ in air mixture) was introduced to and extracted from the chamber at a constant flow rate of 5 L/min. Experimental sessions consisted of 4–6 consecutive 30 min cycles, each comprising a 20 min exposure to air followed by a 10 min exposure to 5% CO_2_. Drug effects were determined using cumulative dosing procedures, and injections were administered following each exposure to 5% CO_2_. Respiratory rate and tidal volume (mL/breath) were recorded over 1 min periods and were multiplied to provide minute volumes. Data from the last three minutes of each exposure to air or CO_2_ were averaged and used for analysis of drug effects on ventilation.

#### 3.4.4. Data Analysis

All statistical analyses and graphic representations were completed with GraphPad Prism version 9.3.0 (GraphPad Software, San Diego, CA, USA) using log-transformed values of doses. Group means ± SEM tail withdrawal latencies (in sec) and minute volume ratios are plotted as a function of drug dose. Data were analyzed using one-way ANOVA with significance set at *p* < 0.05, followed by Dunnett’s multiple comparison test. Animals that did not receive all doses of a drug in tail withdrawal studies because they attained a maximum effect at less than the highest dose were assigned 10 sec latencies for all doses higher than the last dose tested.

### 3.5. X-ray Crystal Data

Single-crystal X-ray diffraction data on compound **15** were collected using Mo Kα radiation and a Bruker SMART APEX II CCD area detector. The crystal was prepared for data collection by coating with high-viscosity microscope oil. The oil-coated crystal was mounted on a micromesh mount (MiTeGen, Inc., Ithaca, NY, USA) and transferred to the diffractometer and a data set collected at 296(2) K. The 0.409 × 0.293 × 0.219 mm^3^ crystal was orthorhombic in space group P2_1_2_1_2, with unit cell dimensions a = 7.24780(10) Å, b = 14.8829(3) Å, c = 19.7753(4) Å, *α* = *β* = *γ* = 90°. Data were 99.0% complete to 29.163° θ (~0.717 Å) with an average redundancy of 6.52. The final anisotropic full matrix least-squares refinement on F^2^ with 244 variables converged at R_1_ = 4.11%, for the observed data and wR2 = 9.40% for all data. The structure was solved by direct methods and refined by full-matrix least-squares on F^2^ values using the programs found in the SHELXL suite (Bruker, SHELXL v2014.7, 2014, Bruker AXS Inc., Madison, WI, USA). Corrections were applied for Lorentz, polarization, and absorption effects. Parameters refined included atomic coordinates and anisotropic thermal parameters for all nonhydrogen atoms. The H atoms were included using a riding model. Complete information on data collection and refinement is available in the Supplementary Materials, Tables S1–S7. It is of note that there is not a hydrogen bond acceptor for H9B. This is likely due to the overwhelming number of van der Waals interactions the oxygen atom and the riding hydrogen are involved in.

Atomic coordinates for **15** have been deposited with the Cambridge Crystallographic Data Centre, deposition number 2258067. Copies of the data can be obtained, free of charge, on application to CCDC, 12 Union Road, Cambridge, CB2 1EZ, UK (fax: +44(0)-1223-336033 or e-mail: deposit@ccdc.cam.ac.uk).

## 4. Conclusions

All 12 diastereomers of the C9-hydroxymethyl, hydroxyethyl, and hydroxypropyl-5-phenylmorphan series were synthesized. The diastereomers had a wide range of activity, as determined in the forskolin-induced cAMP accumulation assay. Several were extremely potent compounds with subnanomolar EC_50_s (**21**, **30**, and **34**), and these potent compounds ranged in efficacy from full agonists (**30** and **34**) to a partial agonist (**21**). Several of the diastereomers synthesized had low potency and efficacy (**16**, **22**, **39**, and **25**). A few were found to be moderately potent DOR agonists (**11**, **30**). For our focus on the evaluation of compounds found to be MOR partial agonists with varied efficacy in the cAMP assay, three of the MOR partial agonists were examined in vivo (one with very low efficacy (**15**, %E_max_ = 26%), another with low efficacy (**36**, %E_max_ = 65%), and the third with good efficacy (**21**, %E_max_ = 85%)). We eliminated fully efficacious MOR agonists from further work because we have observed that potent and fully efficacious MOR agonists exhibit many or all of the side-effects that have been found with morphine [18,24]. Only one of the synthesized hydroxyalkyl diastereomers, **21**, was a potent MOR agonist with good efficacy (EC_50_ = 0.91 nM, E_max_ = 85%). Compound **21** was very unusual in that it was seen to fit into three theories that have been used to probe for an improved antinociceptive. Compound **21** was found to be a partial agonist, and partial agonists have been noted to have fewer side-effects. In agreement with that theory, compound **21** did not fully depress respiration, a major side-effect of opioids. Compound **21** also did not recruit beta-arrestin and therefore its activity might be rationalized using the G-protein bias theory which, put simplistically, notes that G-protein-biased compounds would not show all the side-effects seen with the clinically used opioids. Lastly, it was a MOR–DOR agonist (DOR EC_50_ = 13 nM), although its efficacy at the DOR (E_max_ = 38%) was low. Some MOR–DOR agonists (or MOR agonists and DOR antagonists) have been noted to have fewer side-effects. Compound **21** interacted poorly with the KOR as an antagonist (IC_50_ > 100 nM) and did not have any KOR agonist activity. Since **21** was found in vivo to have morphine-like antinociceptive activity and was unlike morphine in its limited effect in a respiratory depression assay it may hold promise as a useful analgesic with fewer side-effects than those associated with the classical analgesics currently used clinically and, perhaps, as a medication for opioid use disorder. Further work will be carried out with that compound.

## 5. Patents

K.C. Rice: A.E. Jacobson, F. LI, E.S. Gutman, E.W. Bow, Biased potent opioid-like agonists as improved medications to treat chronic and acute pain and methods of using the same. US Application Serial No.: 62/644,791 filed on 19 March 2018. US Patent 11,352,365 issued 6-7-2022. International Publication WO 2019/182950 A1 26 September 2019, USA, p. 91.

## Figures and Tables

**Table 1:** Opioid Receptor Activity Measured in the Forskolin-induced cAMP Accumulation Assay ^a^.

		MOR		DOR		KOR		MOR β-Arrestin Recruitment ^e^	
		Agonist	Antagonist ^b^	Agonist	Antagonist ^c^	Agonist	Antagonist ^d^		
Name	Structure	EC_50_ ± SEM (nM) (%E_max_ ± SEM)	IC_50_ ± SEM (nM) (%I_max_ ± SEM)	EC_50_ ± SEM (nM) (%E_max_ ± SEM)	IC_50_ ± SEM (nM) (%I_max_ ± SEM)	EC_50_ ± SEM (nM) (%E_max_ ± SEM)	EC_50_ ± SEM (nM) (%E_max_ ± SEM)	EC_50_ ± SEM (nM) (%E_max_ ± SEM)	Bias Factor ^f^
	Set 1—C9-Hydroxymethyl								
15 (EG-1-199) 1*S*,5*R*,9*R*	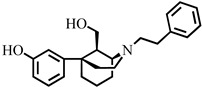	3.8 ± 1.4 (26 ± 4%)	13 ± 6 (96 ± 8%)	0.6 ± 0.3 (17 ± 3%)	336 ± 139 (109 ± 8%)	>10,000	24 ± 5 (107 ± 8%)	>50,000	N/C
16 (EG-1-200) 1*S*,5*R*,9*S*	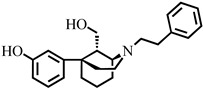	203 ± 38 (54 ± 5%)	N/D	N/D	N/D	N/D	N/D	N/D	
10 (JAL-1-069) 1*R*,5*S*,9*S*	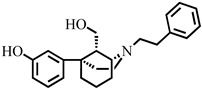	>10,000	91 ± 34 (173 ± 24%)	N/D	N/D	N/D	N/D	N/D	
11 (JAL-1-071) 1*R*,5*S*,9*R*	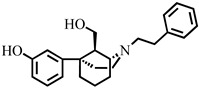	2.0 ± 0.6 (99 ± 2%)	N/D	8.5 ± 3.5 (35 ± 6%)	N/D	>10,000	271 ± 92 (84 ± 6%)	187 ± 75 (2.7 ± 0.4%)	13.4
	Set 2—C9-Hydroxyethyl								
21 (JL-02-0039 and 0111) 1*S*,5*R*,9*R*	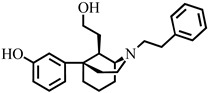	0.9 ± 0.5 (85 ± 5%)	N/D	13 ± 3 (39 ± 3%)	N/D	>10,000	111 ± 45 (83 ± 7%)	>50,000	N/C
22 (JL-02-0042) 1*S*,5*R*,9*S*	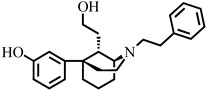	35 ± 4 (83 ± 3%)	N/D	N/D	N/D	N/D	N/D	N/D	
29 (EWB-3-79) 1*R*,5*S*,9*S*	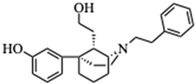	>10,000	52 ± 12 (173 ± 19%)	N/D	N/D	N/D	N/D	N/D	
30 (EWB-3-93) 1*R*,5*S*,9*R*	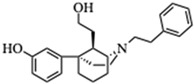	0.2 ± 0.04 (98 ± 1%)	N/D	4.5 ± 2.3 (29 ± 6%)	29 ± 8 (82 ± 10%)	>10,000	493 ± 76 (89 ± 6%)	18 ± 6 (7.2 ± 0.6%)	5.2
	Set 3—C9-Hydroxypropyl								
36 (EG-1-100) 1*S*,5*R*,9*R*	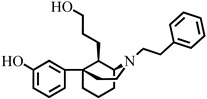	1.2 ± 0.4 (65 ± 6.0%)	N/D	102 ± 16 (41 ± 12%)	N/D	>10,000	180 ± 30 (95 ± 9%)	>50,000	N/C
39 (JAL-2-0067) 1*S*,5*R*,9*S*	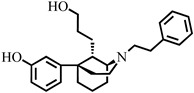	37 ± 19 (37 ± 6%)	1420 ± 265 (106 ± 11%)	N/D	N/D	N/D	N/D	N/D	
35 (EG-1-166) 1*R*,5*S*,9*S*	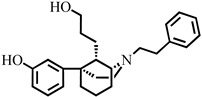	35 ± 13 (52 ± 3%)	N/D	N/D	N/D	N/D	N/D	N/D	
34 (JAL-2-0063) 1*R*,5*S*,9*R*	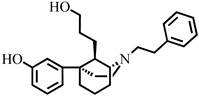	0.3 ± 0.05 (100 ± 1%)	N/D	>10,000	41 ± 5 (111 ± 4%)	>10,000	1913 ± 571 (93 ± 6%)	34 ± 8 (3.1 ± 0.2%)	14.0
	Ether corresponding to phenol 11								
41 (JAL-02-0120) 1*R*,5*S*,9*R*	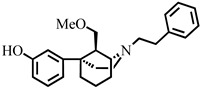	0.7 ± 0.2 (100 ± 1%)	N/D	2.4 ± 1.6 (20 ± 5%)	54 ± 14 (80 ± 8%)	>10,000	1366 ± 361 (92 ± 3%)	29 ± 5 (2.8 ± 0.2%)	6.4
	Standards								
	Morphine	5.8 ± 0.3 (102 ± 0.1%)						383 ± 52 (33 ± 2%)	0.8
	DAMGO	0.3 ± 0.1 (103 ± 1%)						83 ± 4 (103 ± 0.2%)	1.0
	Buprenorphine	1.4 ± 0.4 (100 ± 1%)				3.6 ± 0.9 (66 ± 7%)			
	U50488H					0.3 ± 0.03 (100 ± 0.3%)			
	SNC80			1.7 ± 0.2 (79 ± 2%)					
	Naltrexone	2.1 ± 1.2 (30 ± 6%)	11 ± 1 (104 ± 1%)	>10,000	295 ± 47.5 (99 ± 1%)	0.6 ± 0.3 (57 ± 7%)	5.5 ± 1.0 (41 ± 7%)		
	nor-BNI						2.3 ± 0.3 (102 ± 1%)		
	Naltrindole				1.1 ± 0.2 (107 ± 5%)				

^a^ Inhibition of forskolin-induced cAMP accumulation; cAMP Hunter^TM^ Chinese hamster ovary cells (CHO-K1) that express human μ-opioid receptor (OPRM1), human κ-opioid receptor (OPRK1), and human δ-opioid receptor (OPRD1) were used for the forskolin-induced cAMP accumulation assay to determine potency and efficacy of the compounds following the previously established methods [22]; to determine % efficacy in forskolin-induced cAMP assays, data were blank subtracted with the vehicle control, followed by normalization to the forskolin control. Data were then analyzed in GraphPad Prism 8 (GraphPad, LaJolla, CA, USA) using nonlinear regression; values are expressed as the mean ± SEM of at least three independent experiments; N/D = not determined. ^b^ MOR antagonist potency (IC_50_) determined versus EC_90_ of fentanyl; degree of antagonism (I_max_) normalized to naltrexone. ^c^ DOR antagonist potency (IC_50_) determined versus EC_50_ of SNC80; degree of antagonism (I_max_) normalized to naltrexone. ^d^ KOR antagonist potency (IC_50_) determined versus EC_90_ of U50488H; degree of antagonism (Imax) normalized to *nor*-BNI. ^e^ β-arrestin recruitment; PathHunter CHO-K1 OPRM1 β-arrestin cells were used to determine potency and efficacy following the previous established methods [23]; to determine % efficacy in β-arrestin recruitment, data were blank subtracted with vehicle control followed by normalization to the maximum response of DAMGO. Data were then analyzed in GraphPad Prism 8 (GraphPad, LaJolla, CA, USA) using nonlinear regression; values are expressed as the mean ± SEM of at least three independent experiments; N/D = not determined. ^f^ Bias factors were calculated using equations as described in the Materials and Methods. DAMGO is the reference compound, with a bias factor = 1. Bias factors > 1 indicate bias toward the cAMP pathway in compared to DAMGO and bias factors < 1 indicate bias toward the β-arrestin2 pathway in comparison to DAMGO. N/C = not calculable (the bias factor equations use log (EC_50_/E_max_) during the calculation, and it is not calculable when the E_max_ = 0).

## Data Availability

The data presented in this study are available in this article or in the supplementary material.

## Supplementary Materials

The following supporting information can be downloaded at: https://www.mdpi.com/article/10.3390/molecules28124795/s1, ^1^H and ^13^C-NMR spectra of novel compounds (Figures S1–S26) and crystal data, atomic coordinates from X-ray crystallographic determination of **15** (Tables S1–S7).

## Sample Availability

Not available.

## Abbreviations

MOR: mu-opioid receptor; DOR, delta-opioid receptor; KOR, kappa-opioid receptor; cAMP, cyclic adenosine monophosphate; DAMGO, [D-Ala2, N-Me-Phe4, Gly5-ol]-enkephalin.
