# Supplementary material for: Discovery of a Potent Highly Biased MOR Partial Agonist among Diastereomeric C9-Hydroxyalkyl-5-phenylmorphans
Source: Molecules. 2023 Jun 15;28(12):4795. doi: 10.3390/molecules28124795 (PMC10304876; doi:10.3390/molecules28124795)
Supplement: Supplementary file 1 [file molecules-28-04795-s001.zip › molecules-2396687-supplementary.pdf]

SUPPLEMENTARY DATA

Discovery of a Potent Highly Biased MOR Partial Agonist Among Diastereomeric C9-Hydroxyalkyl-5-phenylmorphans

Joshua A. Lutz[a], Agnieszka Sulima[a], Eugene S. Gutman[a], Eric W. Bow[a], Dan Luo[b], Sophia Kaska[b], Thomas E. Prisinzano[b], Carol A. Paronis[c], Jack Bergman[c], Gregory H. Imler [d], Andrew T. Kerr[d], Arthur E. Jacobson[a,*], Kenner C. Rice[a,*]

[a]Drug Design and Synthesis Section, Molecular Targets and Medications Discovery Branch, Intramural Research Program, National Institute on Drug Abuse and the National Institute on Alcohol Abuse and Alcoholism, National Institutes of Health, Department of Health and Human Services, 9800 Medical Center Drive, Bethesda, MD 20892-3373, USA

[b]Department of Pharmaceutical Sciences, College of Pharmacy, University of Kentucky, 789 S. Limestone Street, Lexington, Kentucky 40536, USA

[c]McLean Hospital / Harvard Medical School, 115 Mill Street, Belmont MA 02478, USA

[d]Center for Biomolecular Science and Engineering, Naval Research Laboratory, Washington DC, 20375-0001, USA

*Correspondence: arthurj@nida.nih.gov (A.E.J.); kennerr@nida.nih.gov (K.C.R.)

Tel.: +1-301-451-5028 (A.E.J.); +1-301-451-4799 (K.C.R.)

Supplementary Data

**^{1}H and ^{13}C NMR spectra**	**Figures S1-S26**	Pages 2-28, respectively
**X-ray Spectroscopic data**	**Tables S1-S7**	Pages 29-40, respectively

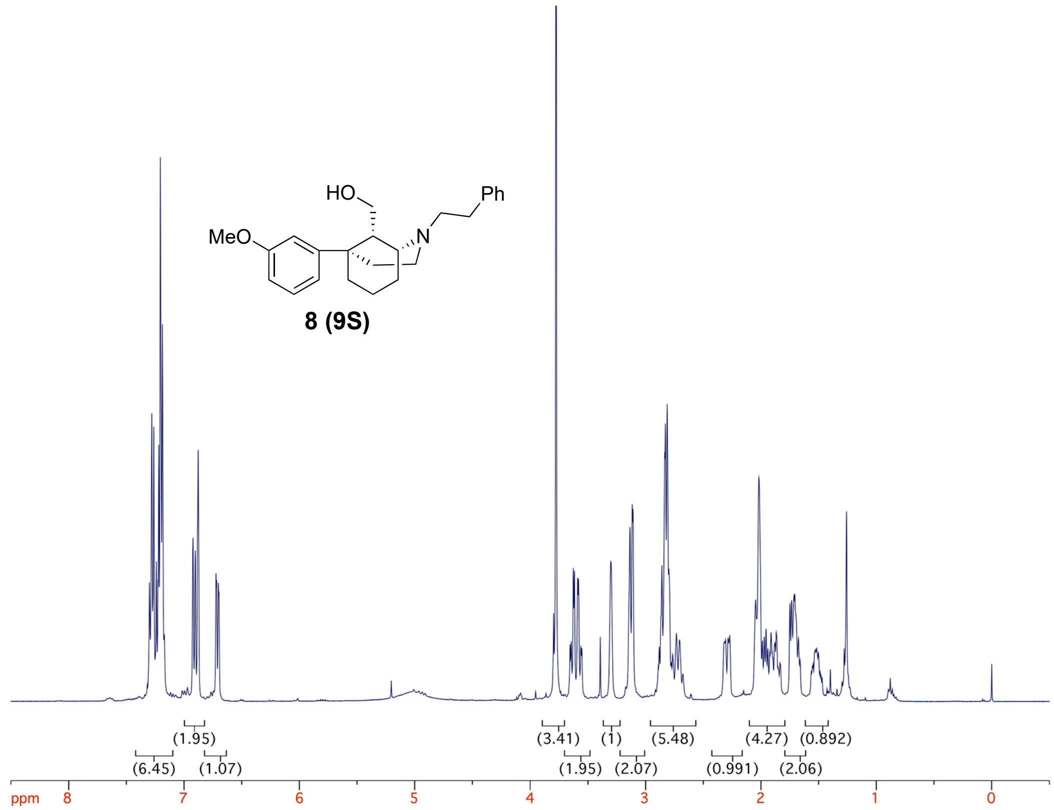

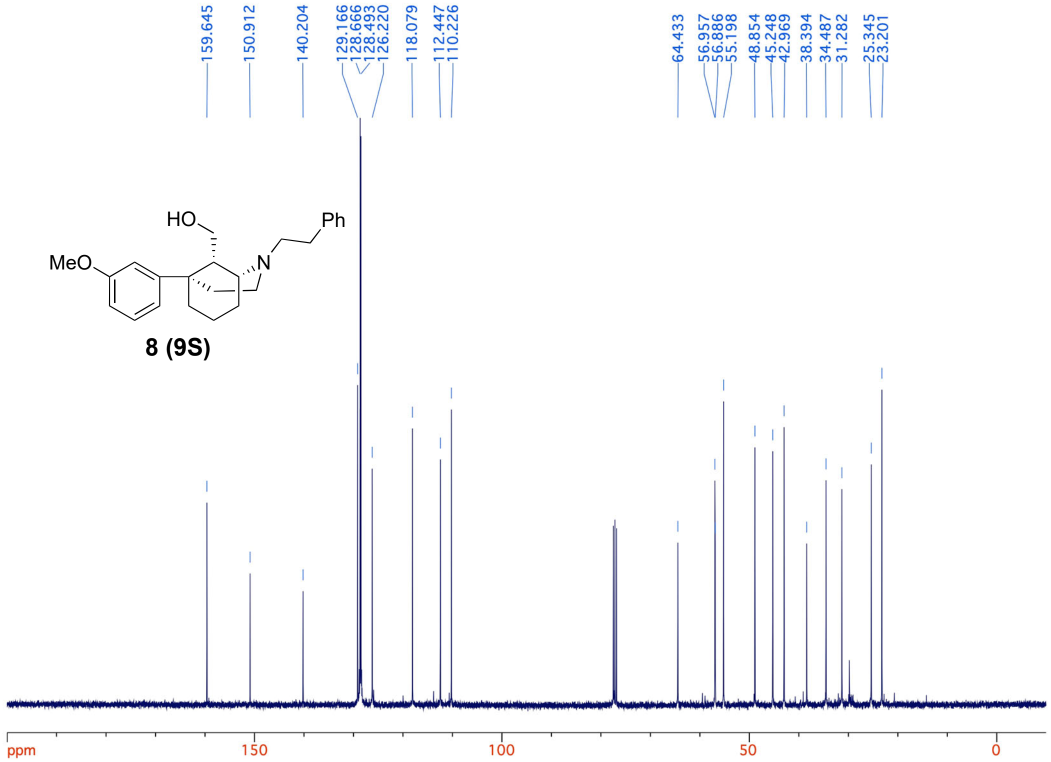

Figure S1: ^{1}H and ^{13}C-NMR spectra of compound **8**

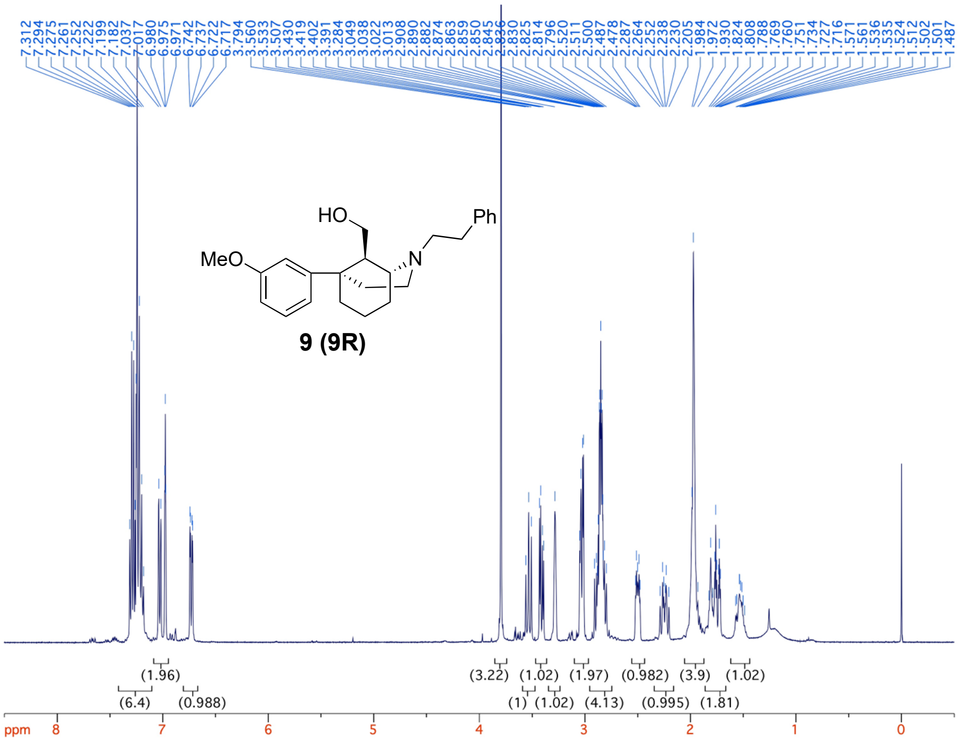

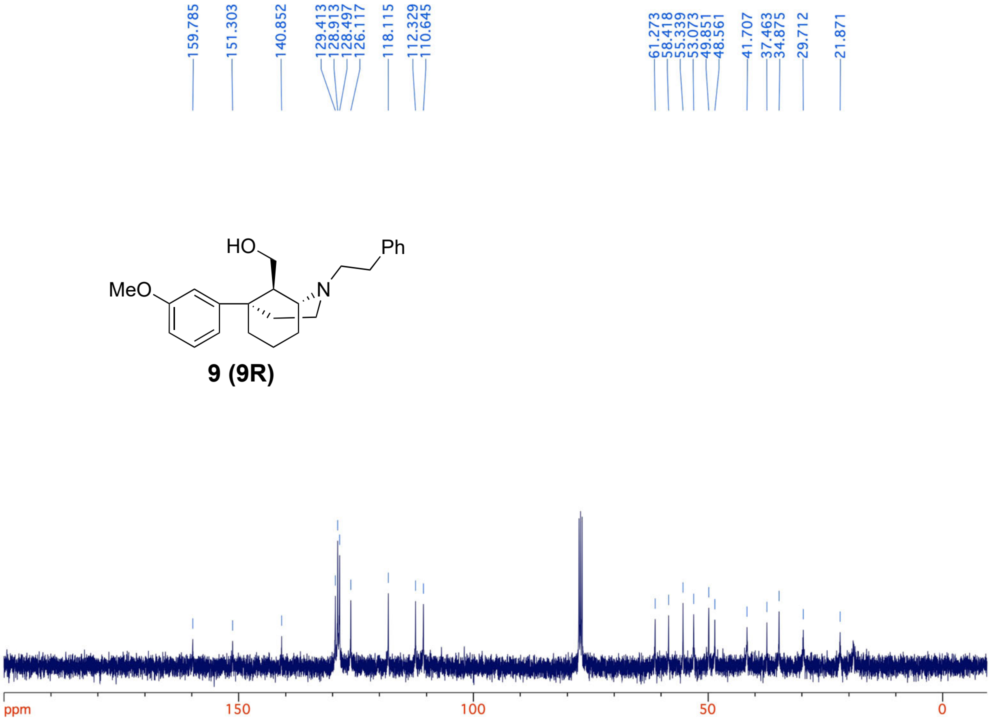

Figure S2: ^{1}H and ^{13}C-NMR spectra of compound **9**

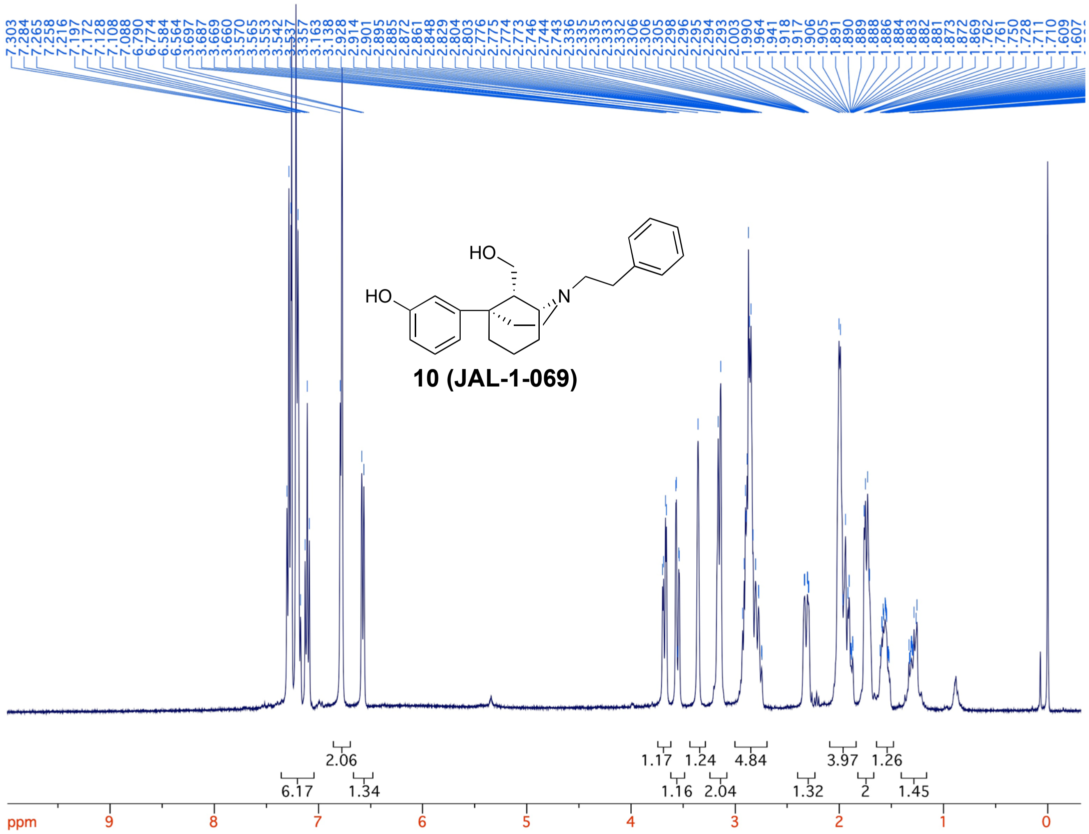

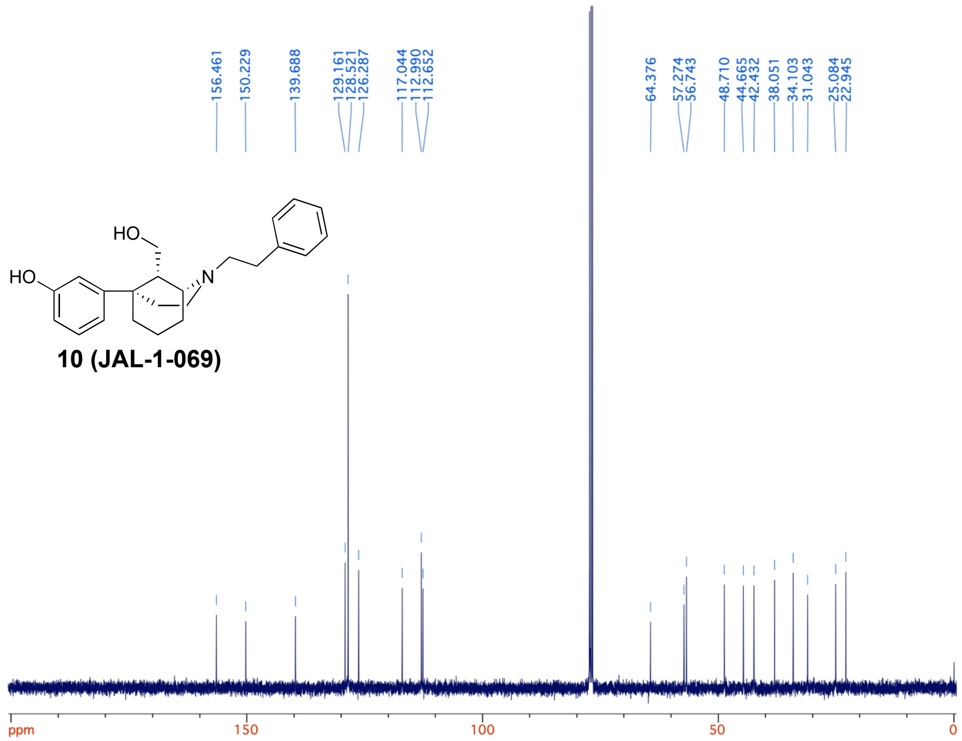

Figure S3: ^{1}H and ^{13}C-NMR spectra of compound **10**

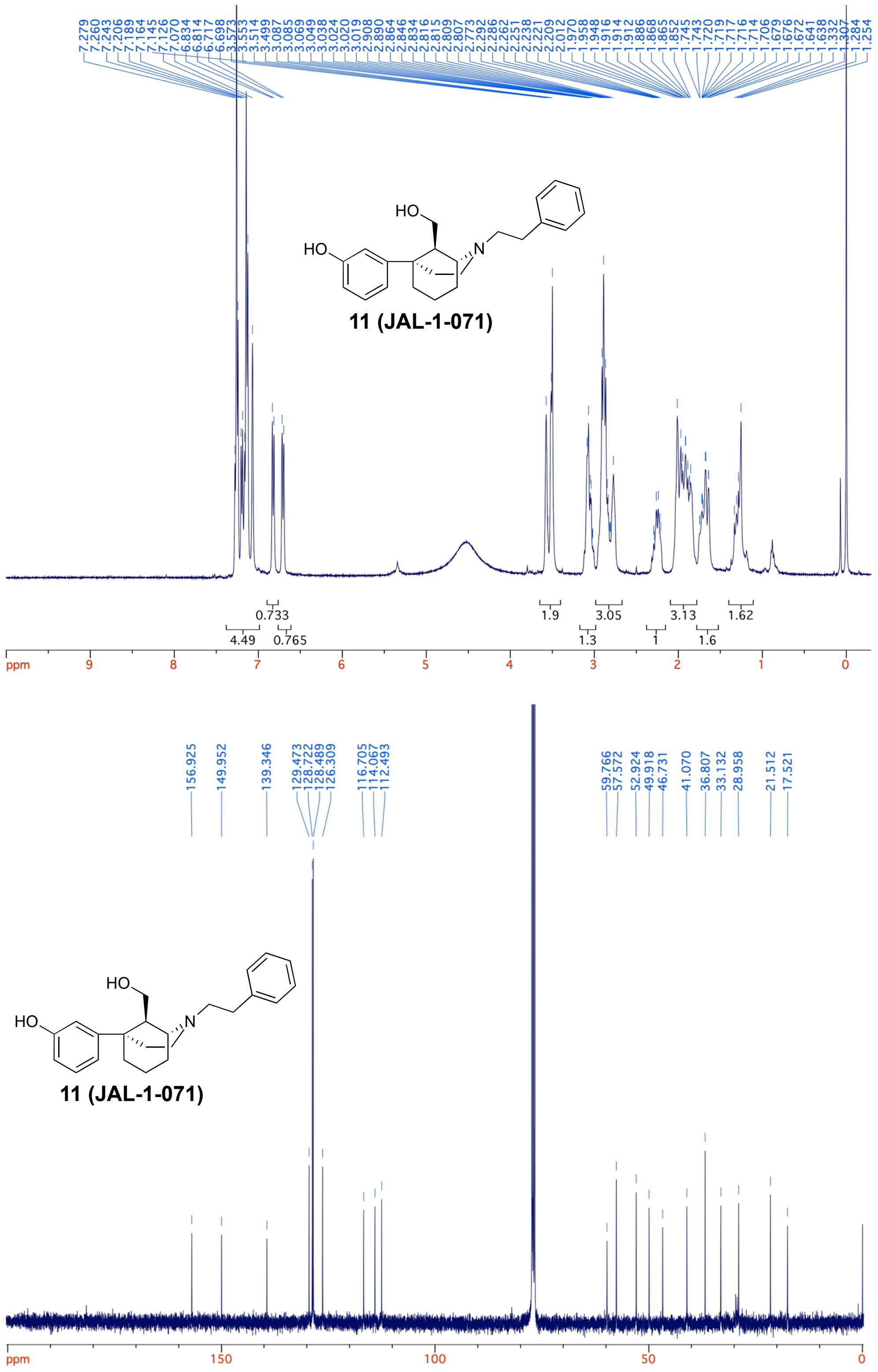

Figure S4: ^{1}H and ^{13}C-NMR spectra of compound **11**

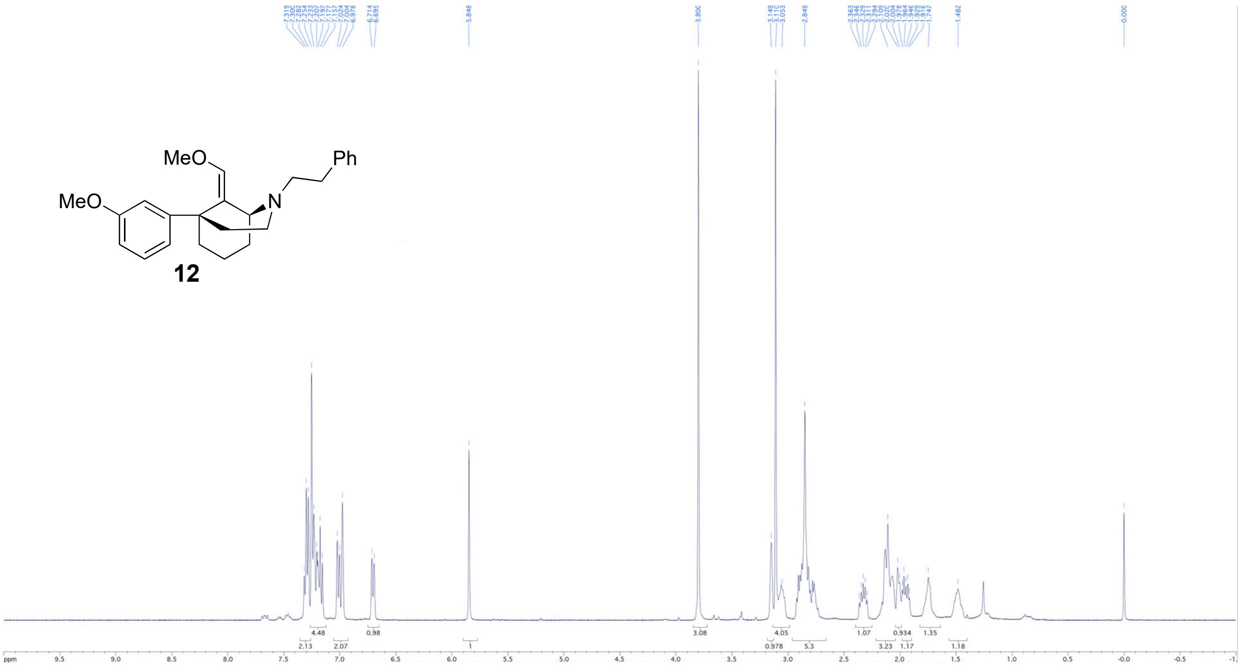

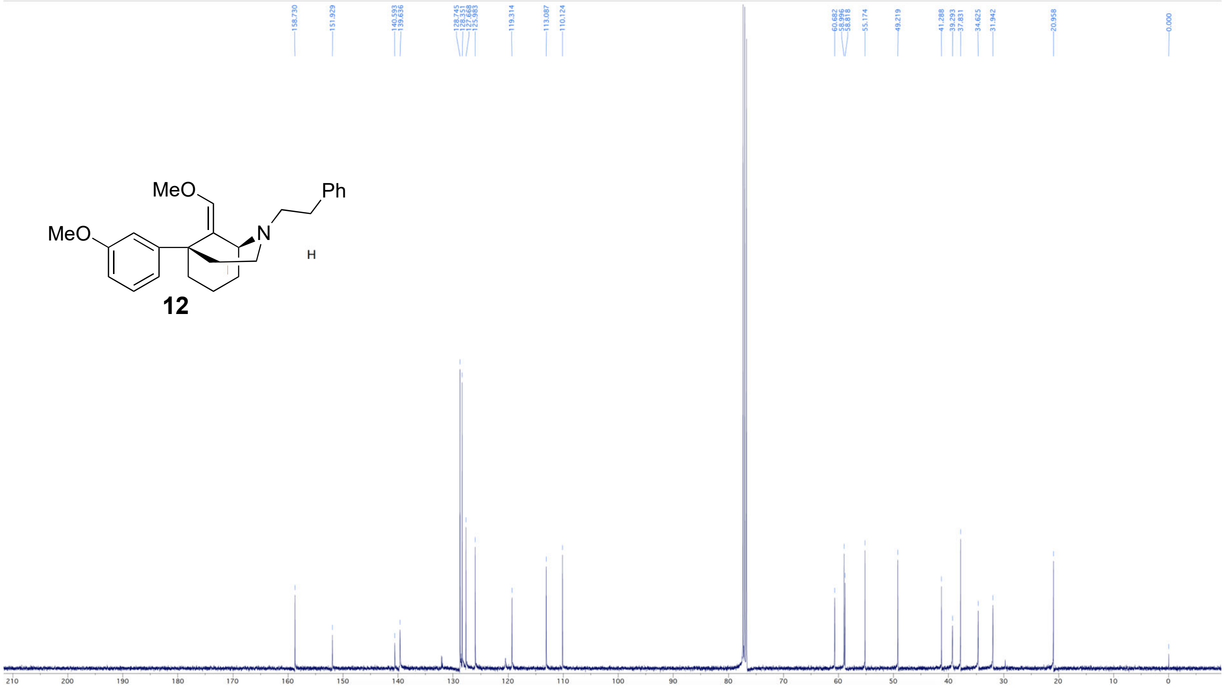

Figure S5: ^{1}H and ^{13}C-NMR spectra of compound **12**

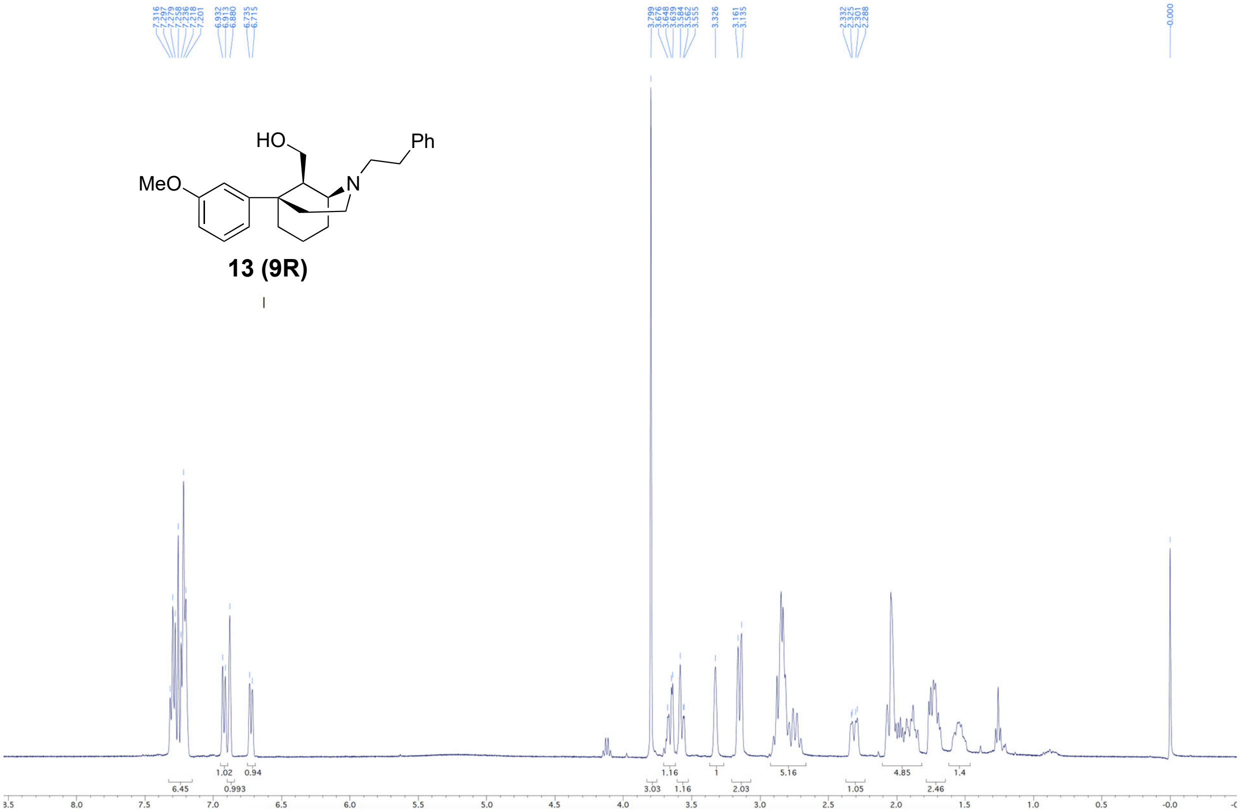

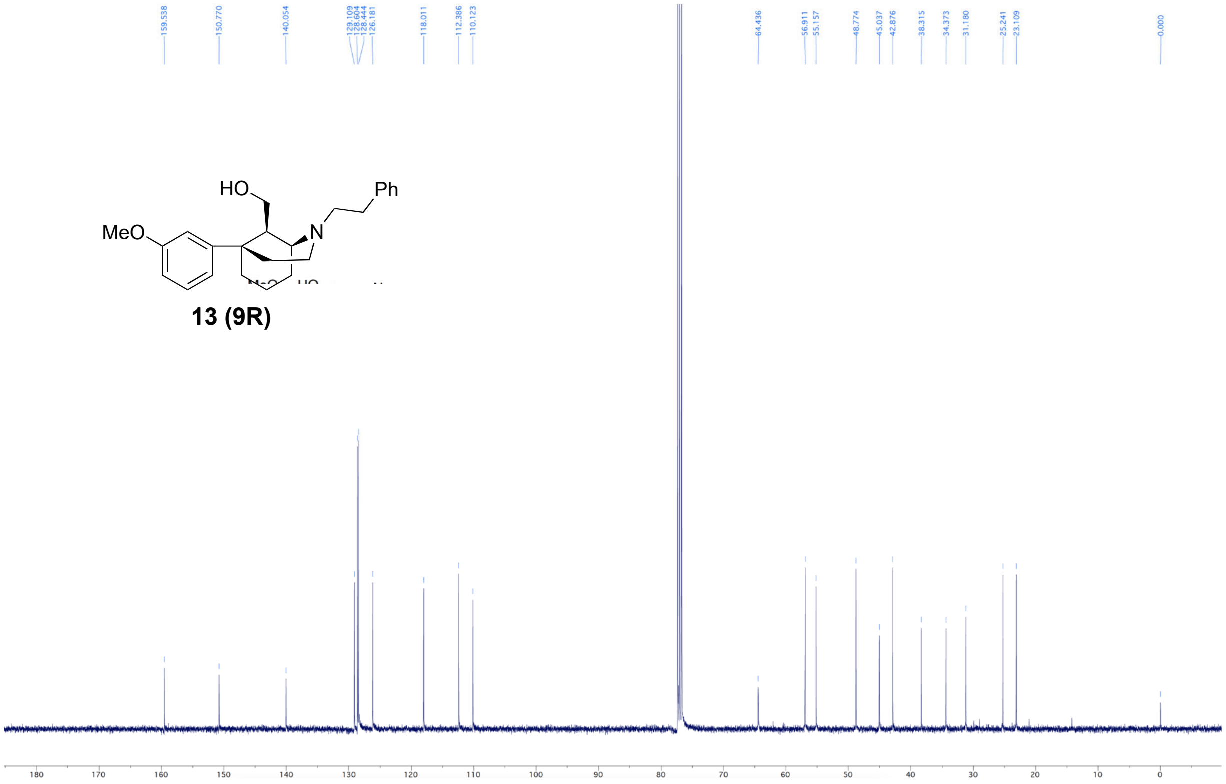

Figure S6: ^{1}H and ^{13}C-NMR spectra of compound **13**

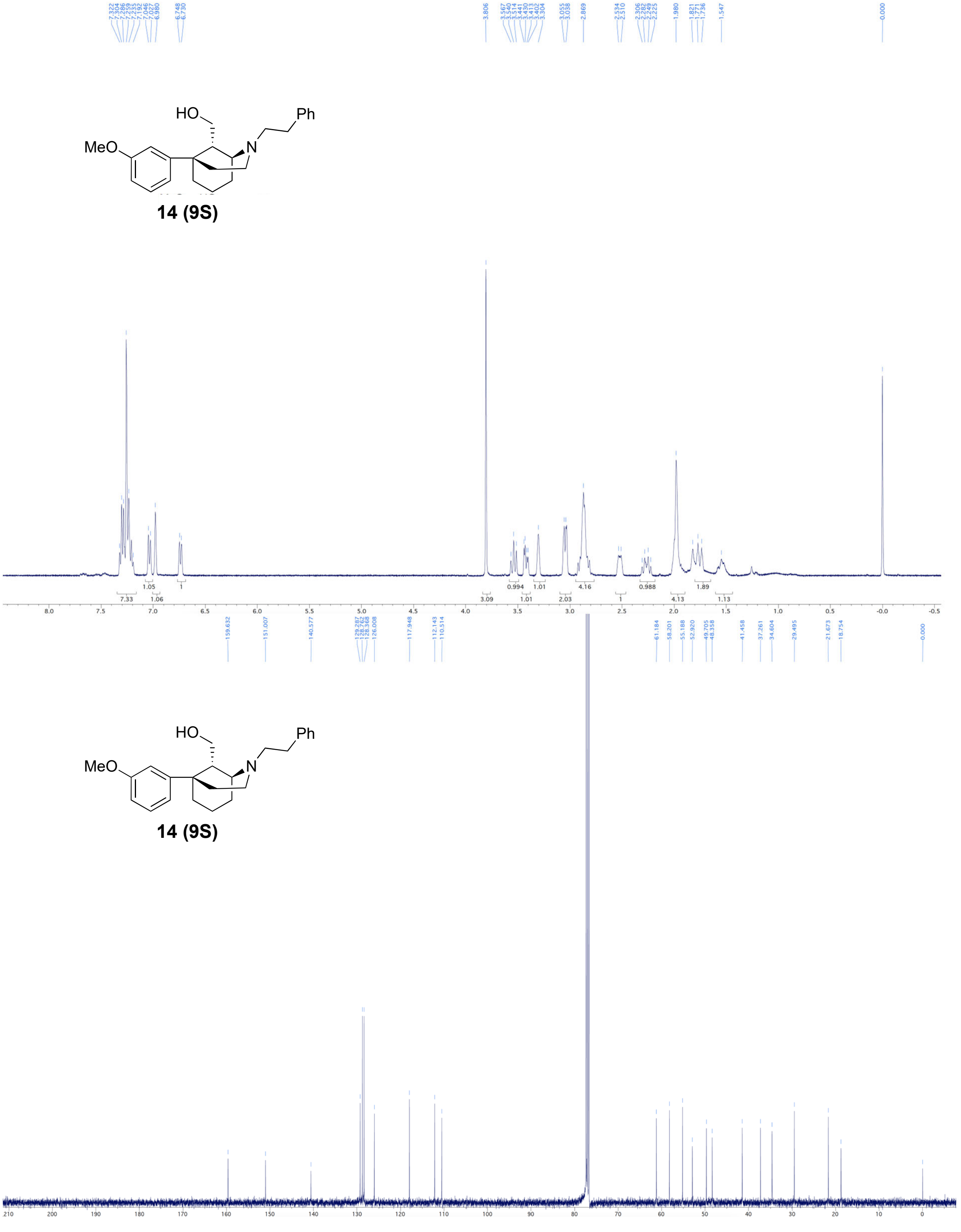

Figure S7: ^{1}H and ^{13}C-NMR spectra of compound 1**4**

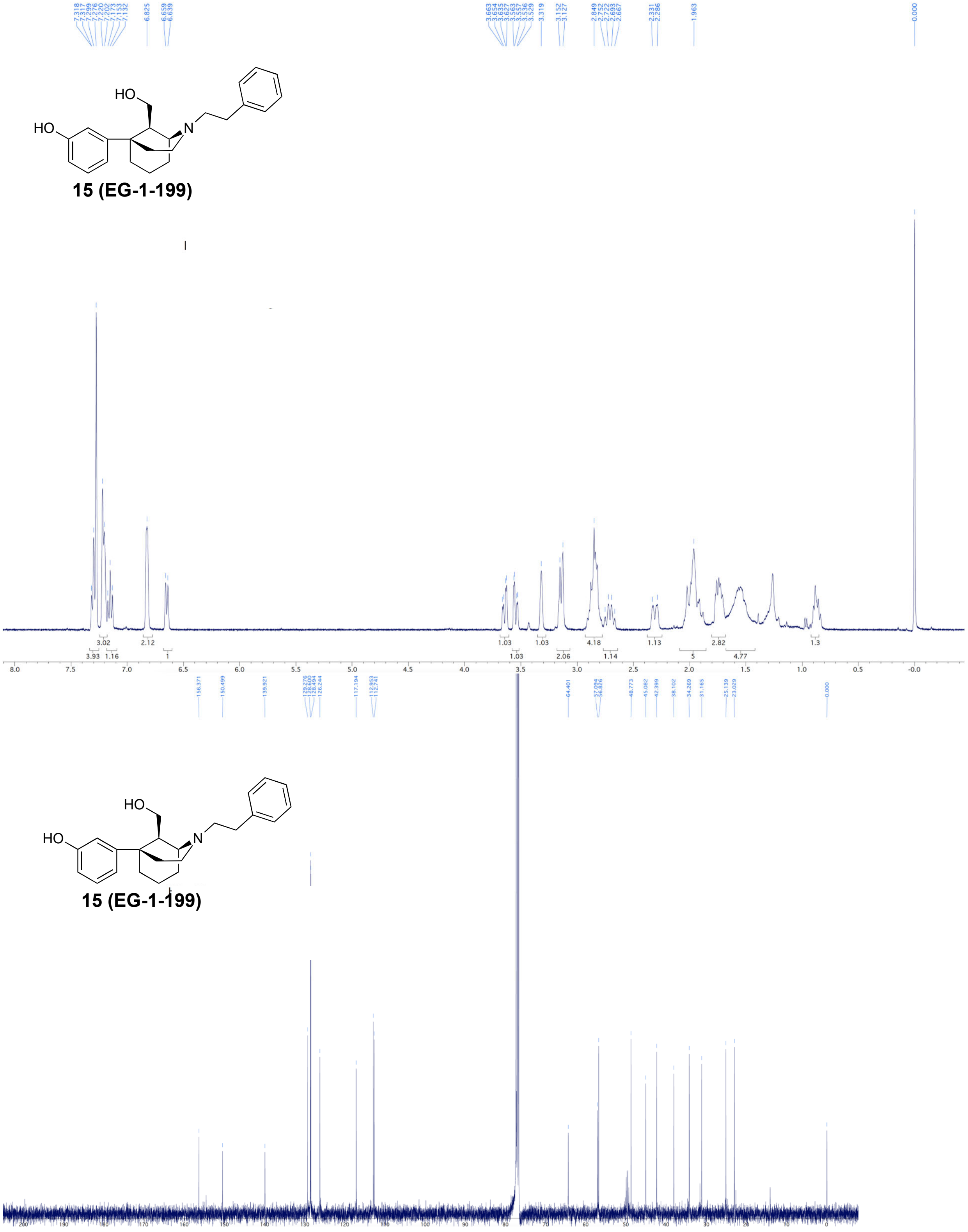

Figure S8: ^{1}H and ^{13}C-NMR spectra of compound **15**

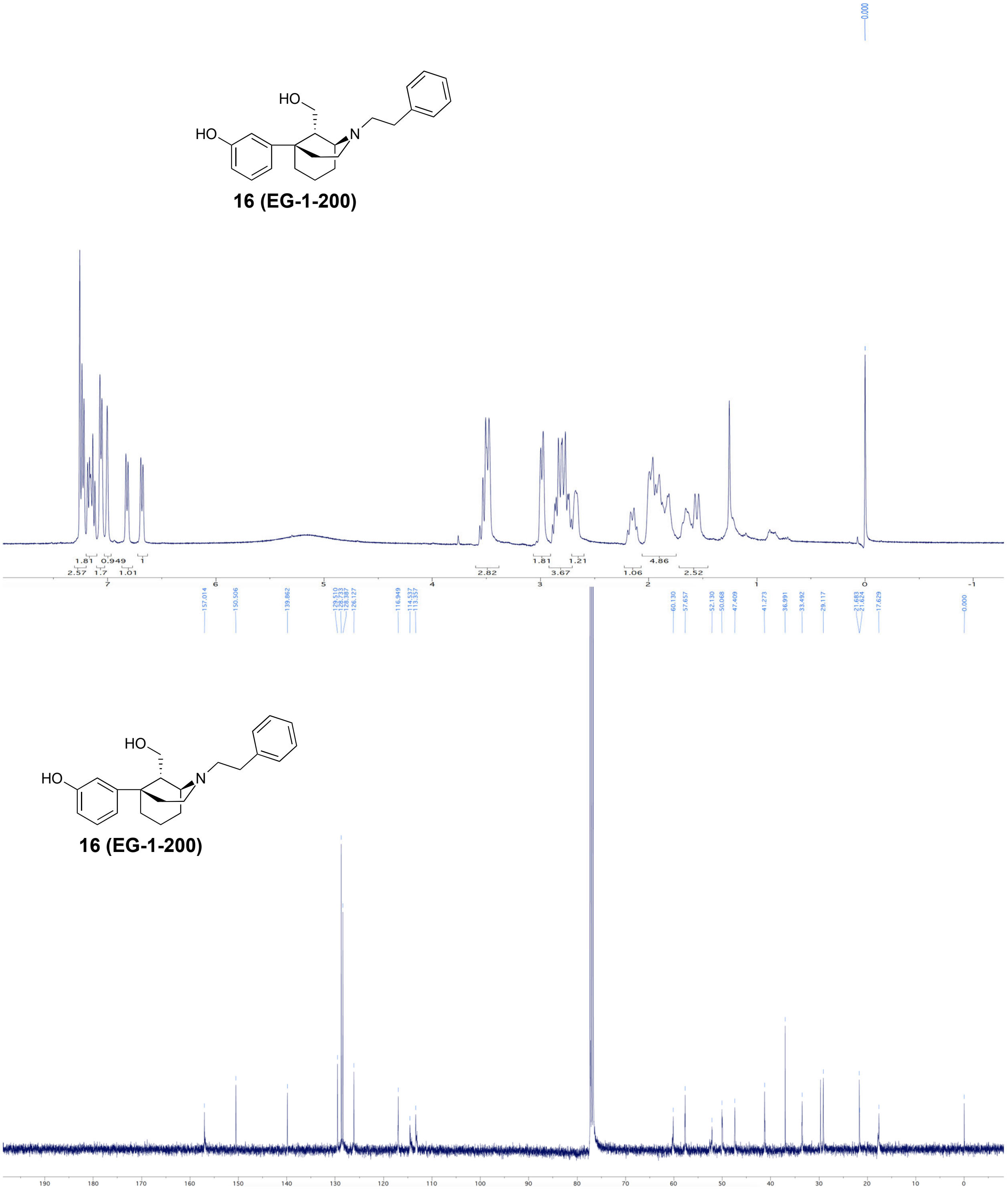

Figure S9: ^{1}H and ^{13}C-NMR spectra of compound **16**

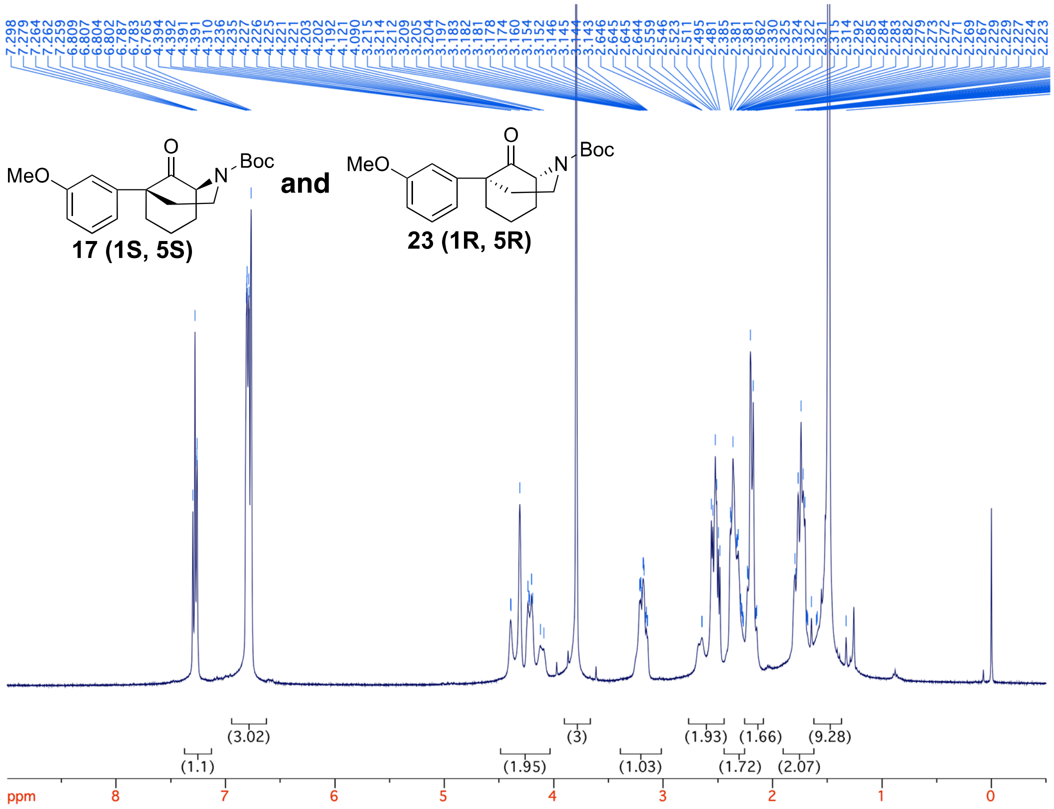

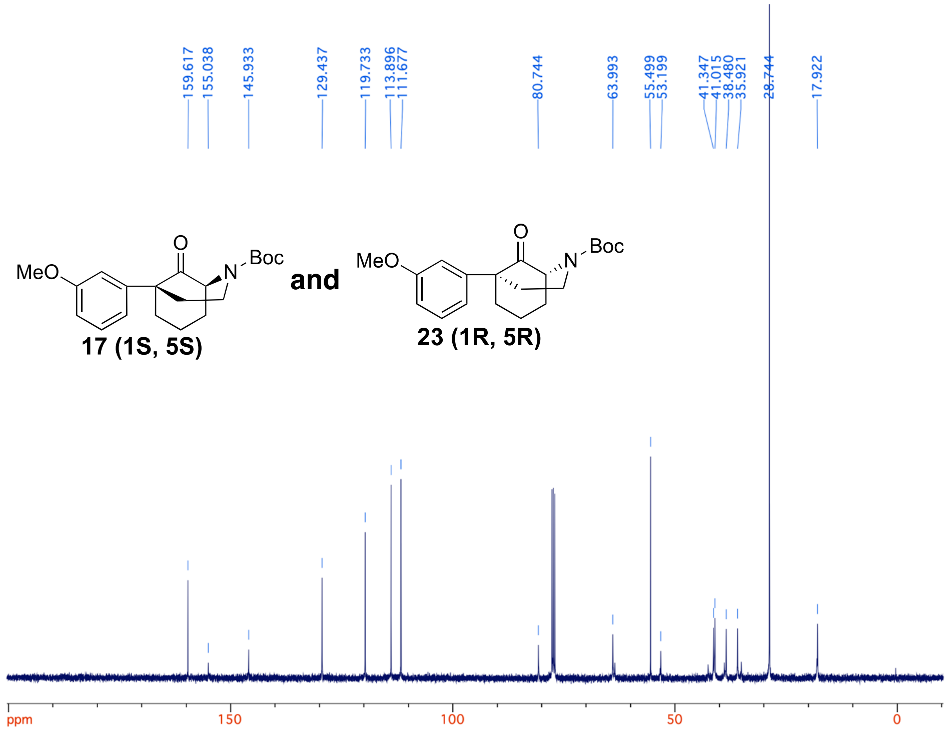

Figure S10: ^{1}H and ^{13}C-NMR spectra of compound **17** and **23**

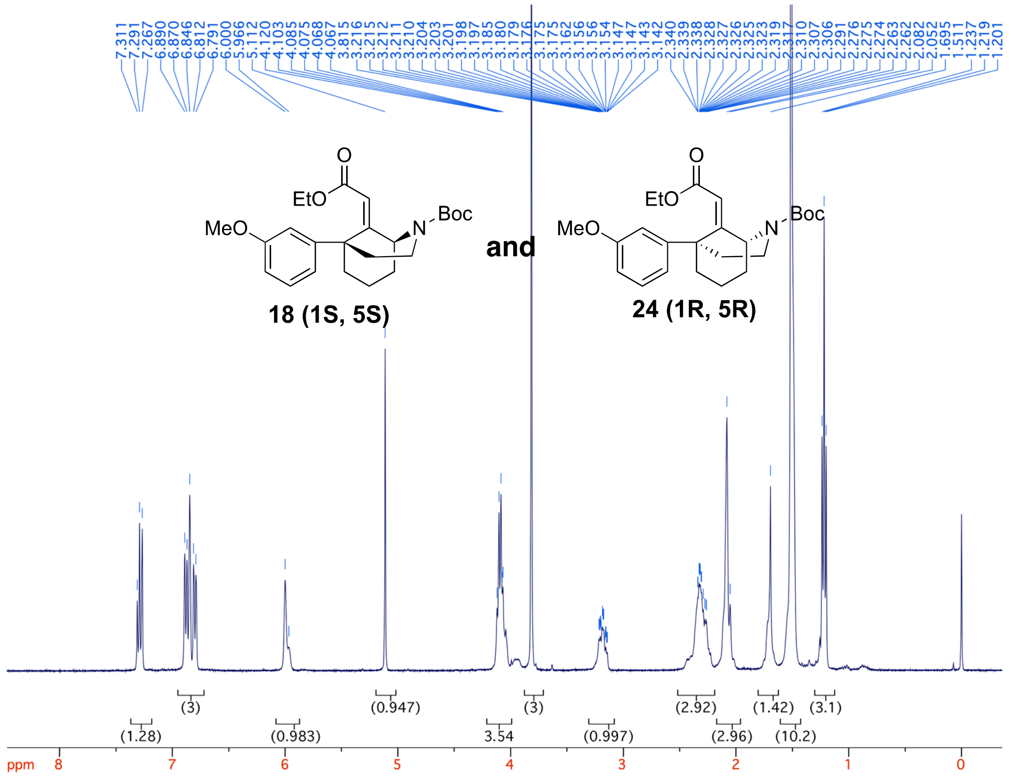

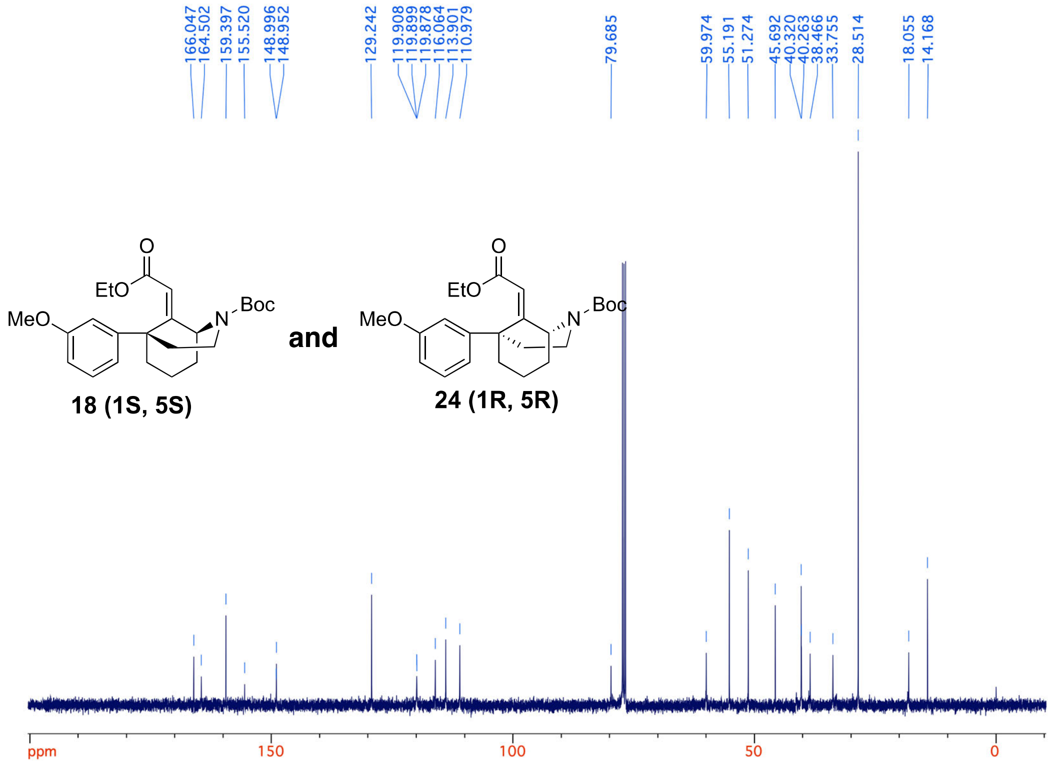

Figure S11: ^{1}H and ^{13}C-NMR spectra of compound **18** and **24**

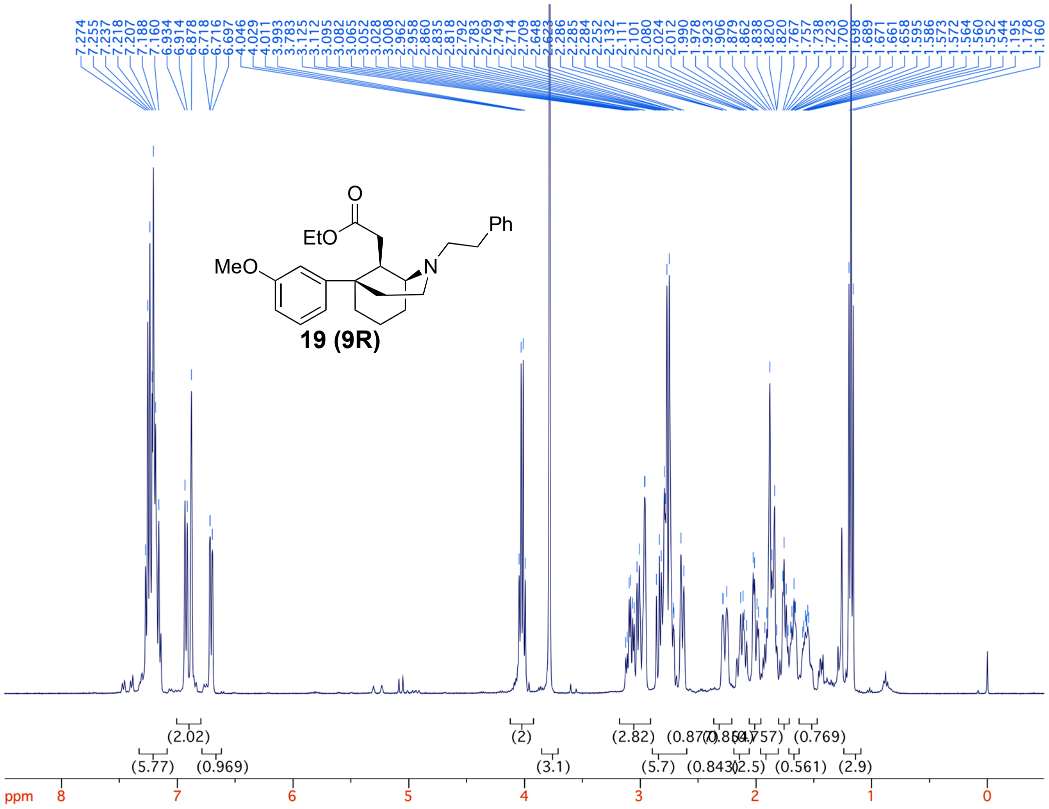

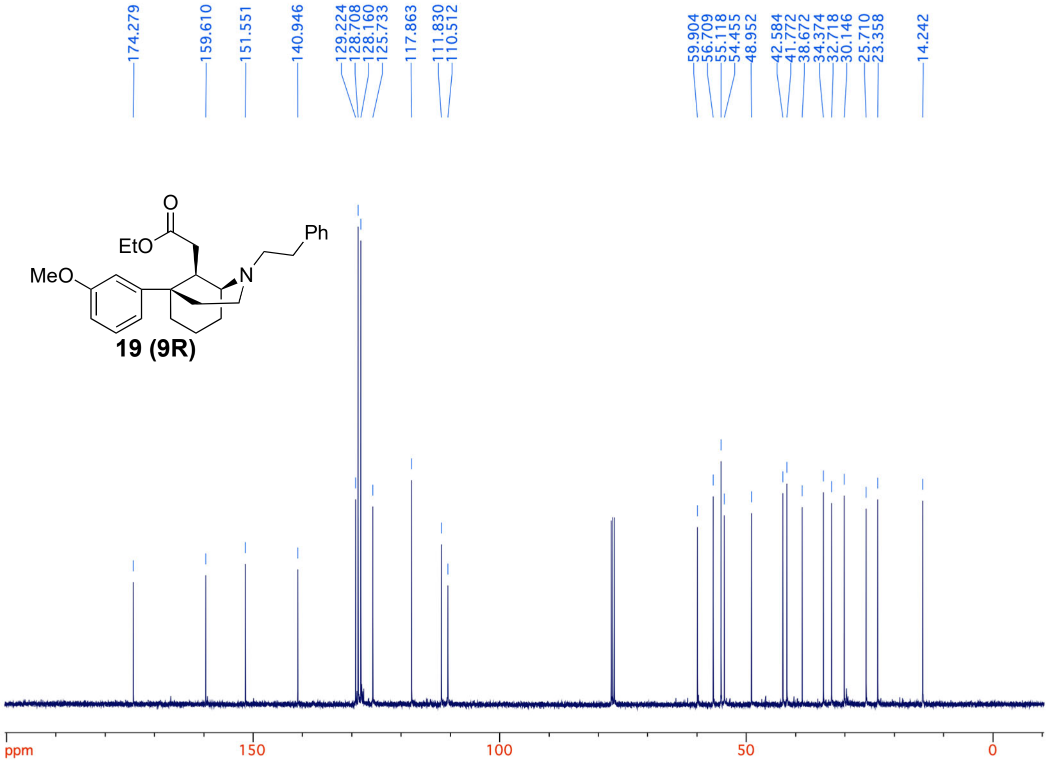

Figure S12: ^{1}H and ^{13}C-NMR spectra of compound **19**

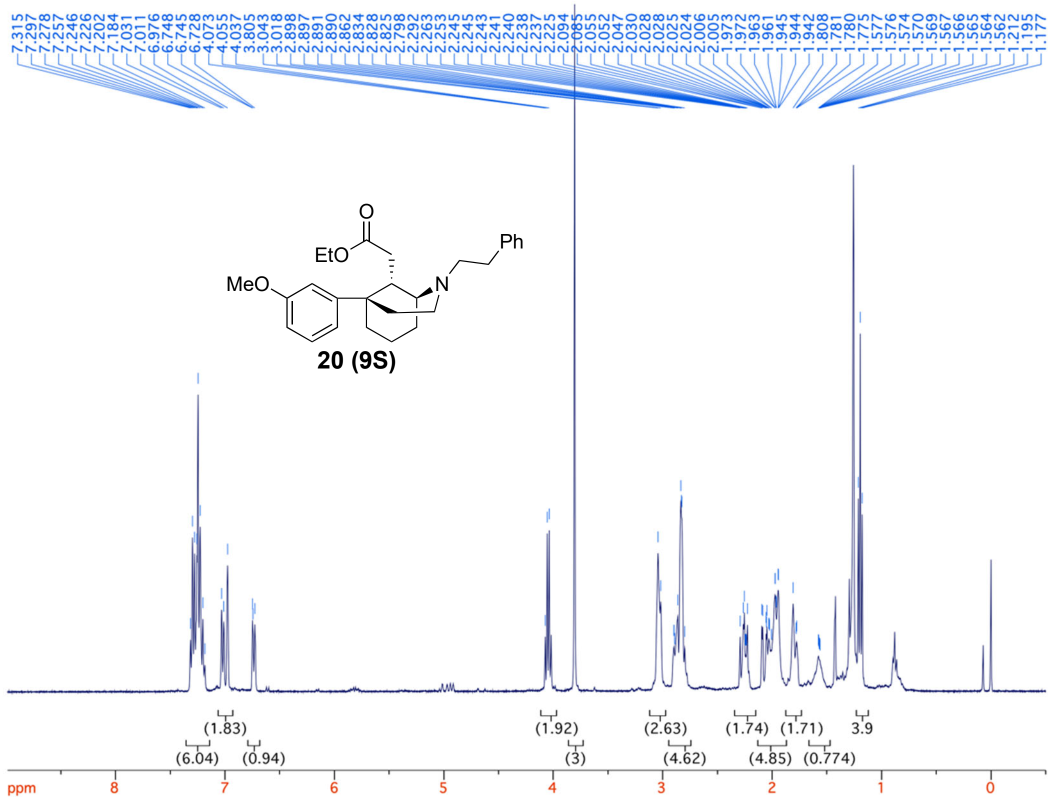

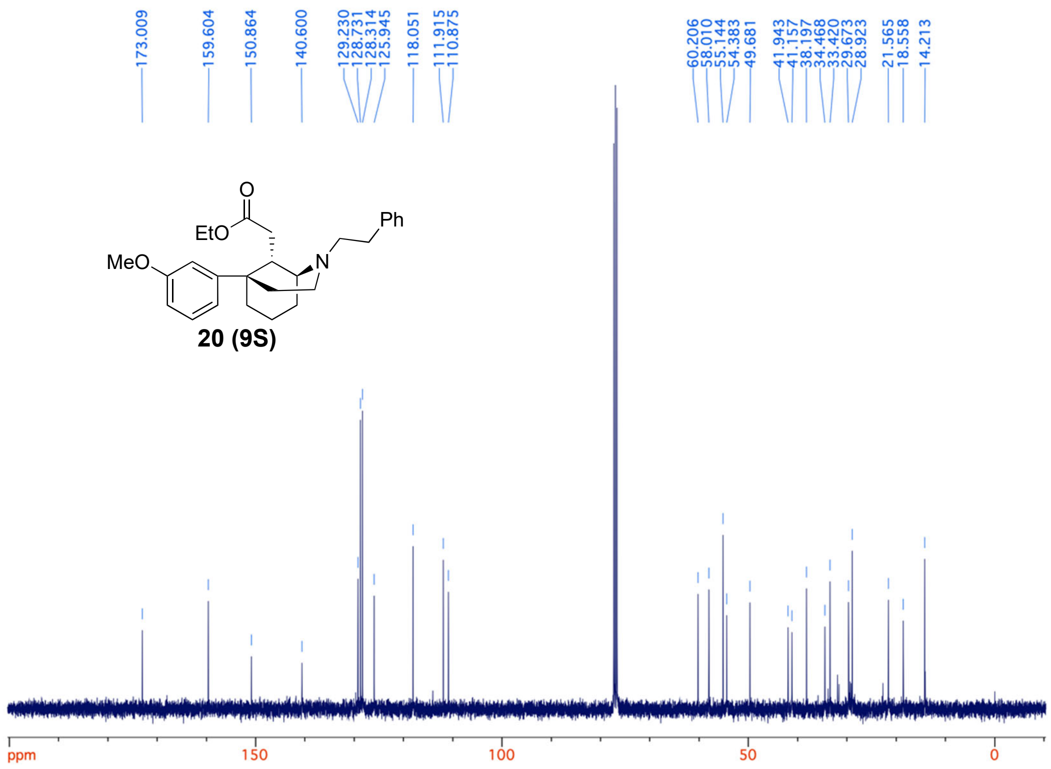

Figure S13: ^{1}H and ^{13}C-NMR spectra of compound **20**

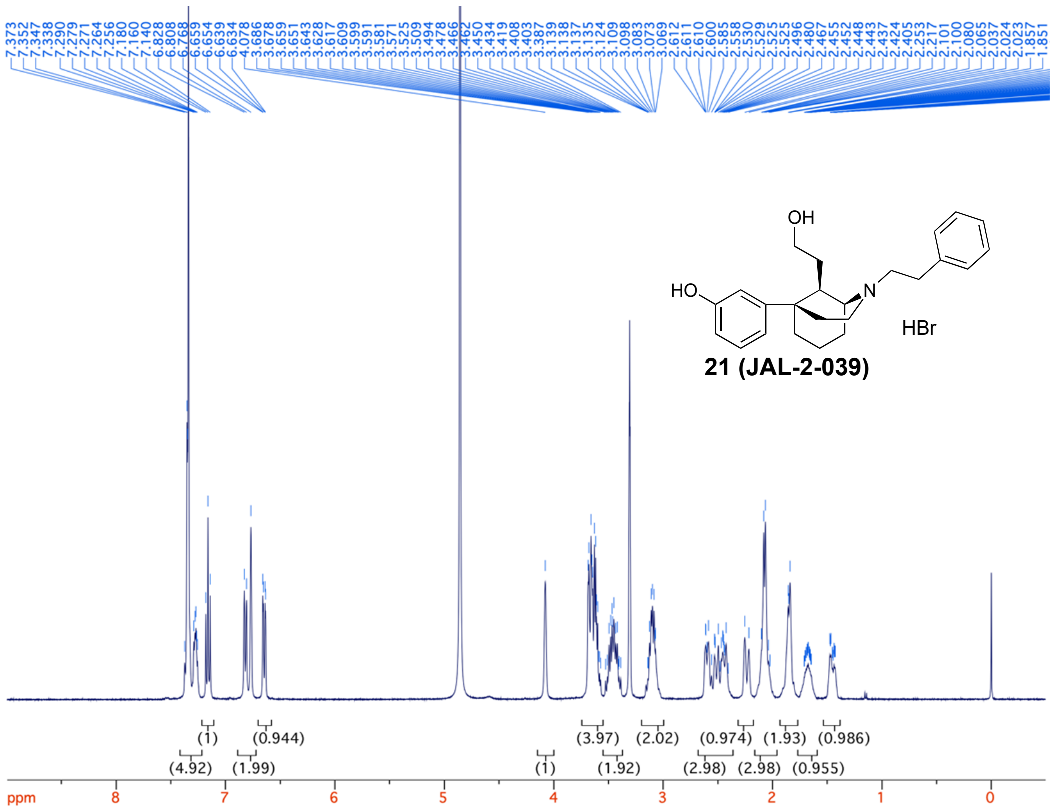

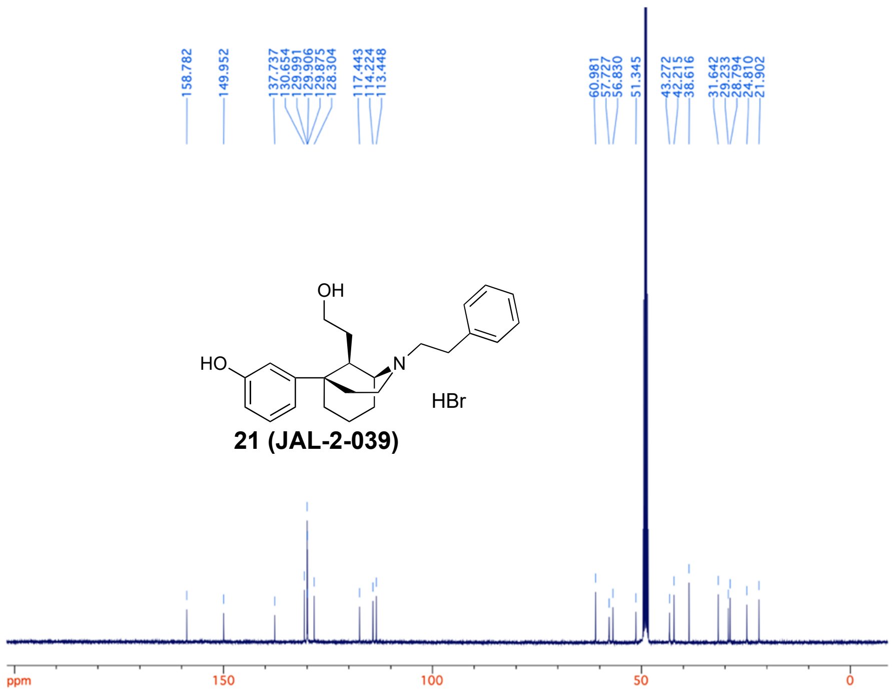

Figure S14: ^{1}H and ^{13}C-NMR spectra of compound **21**

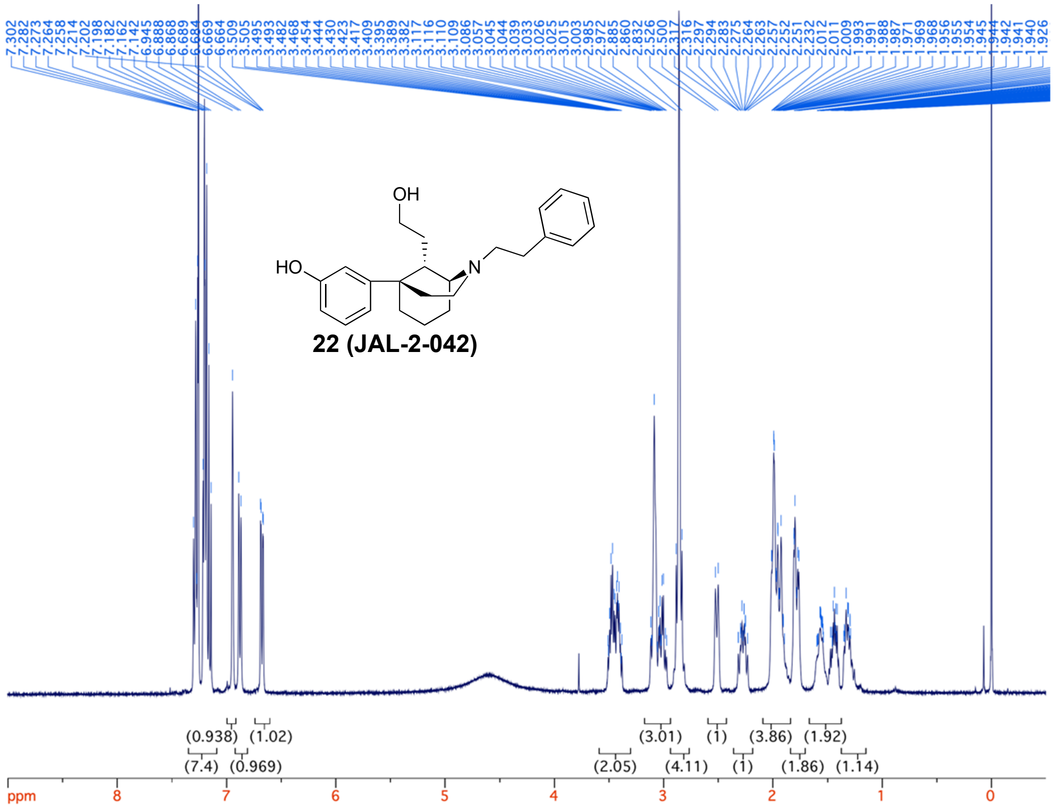

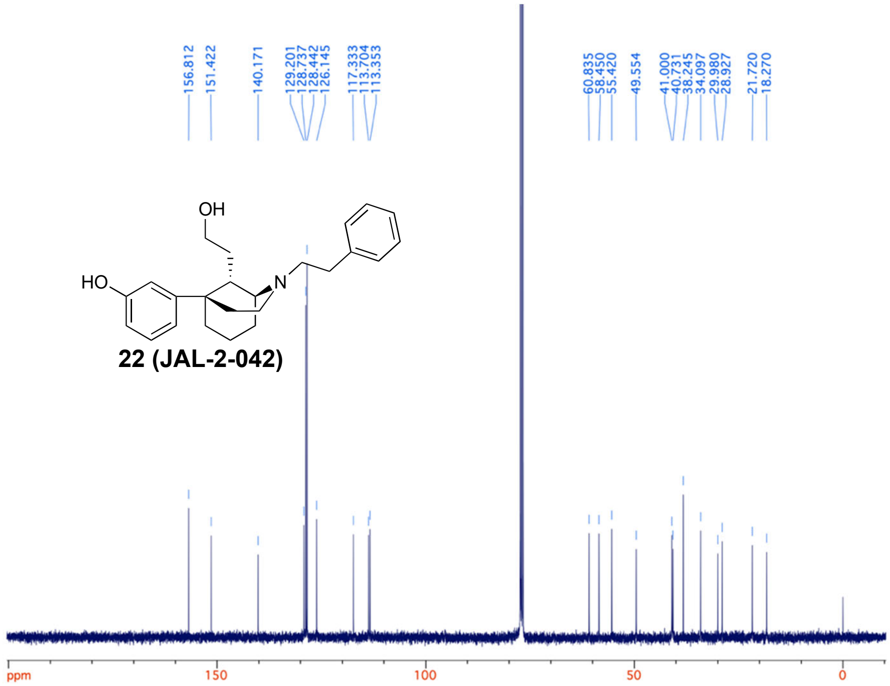

Figure S15: ^{1}H and ^{13}C-NMR spectra of compound **22**

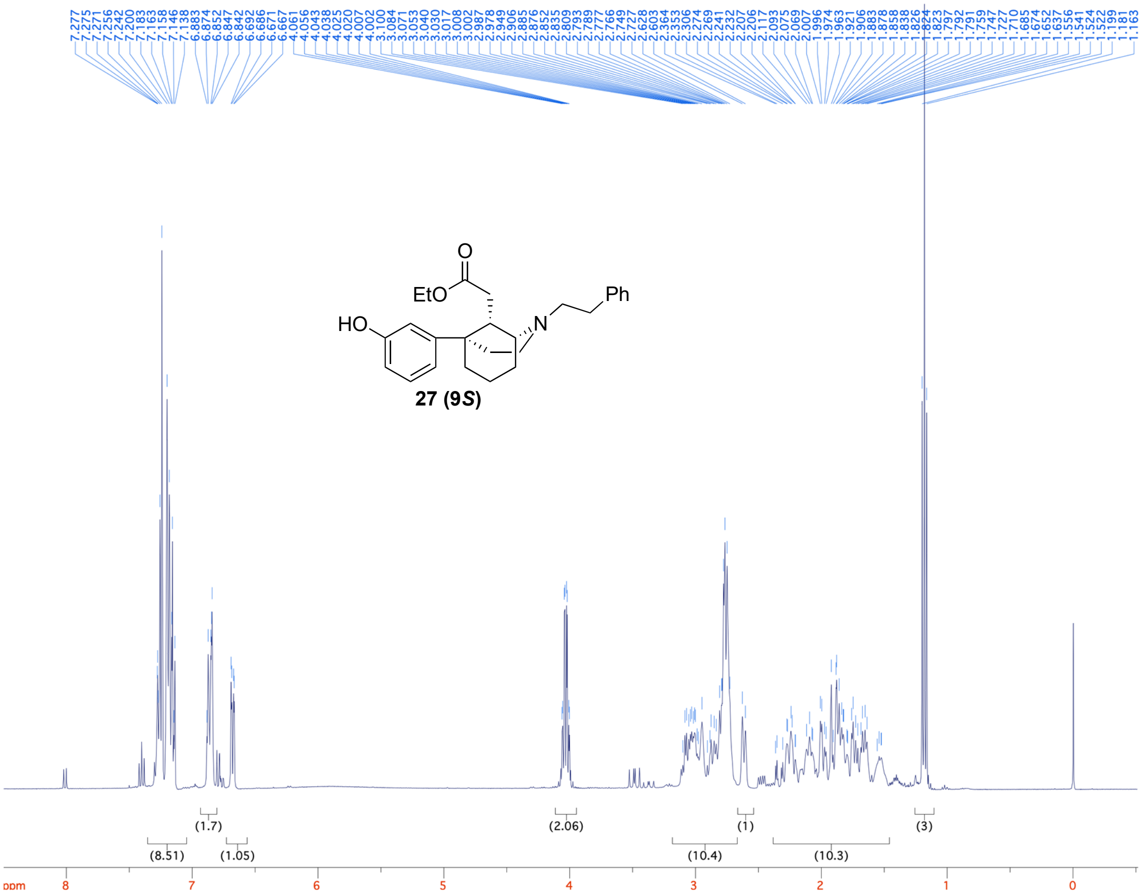

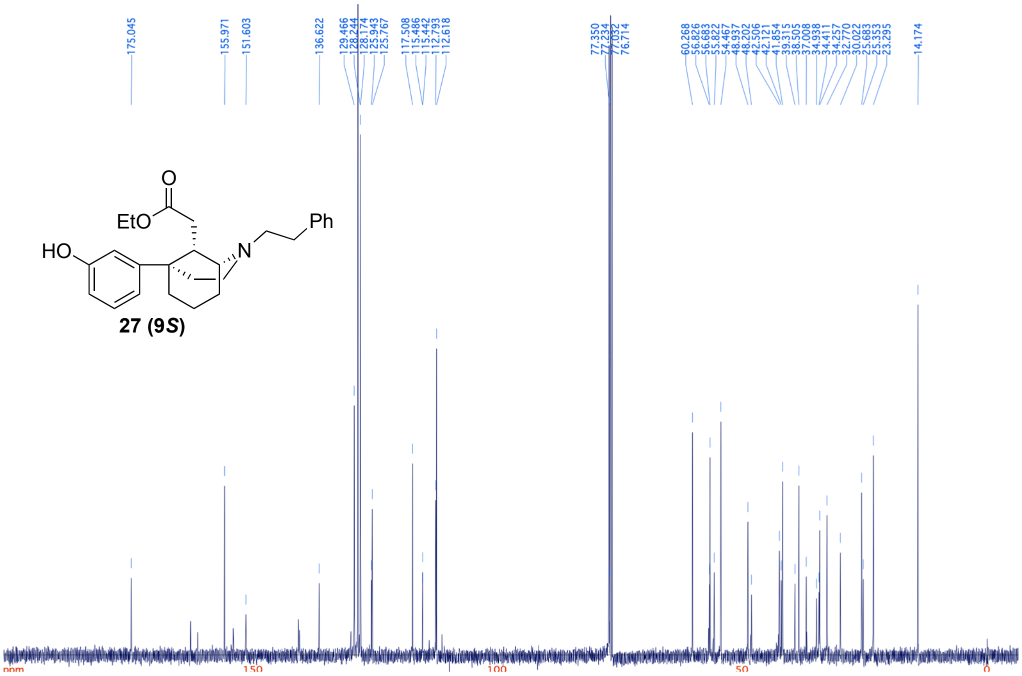

Figure S16: ^{1}H and ^{13}C-NMR spectra of compound **27**

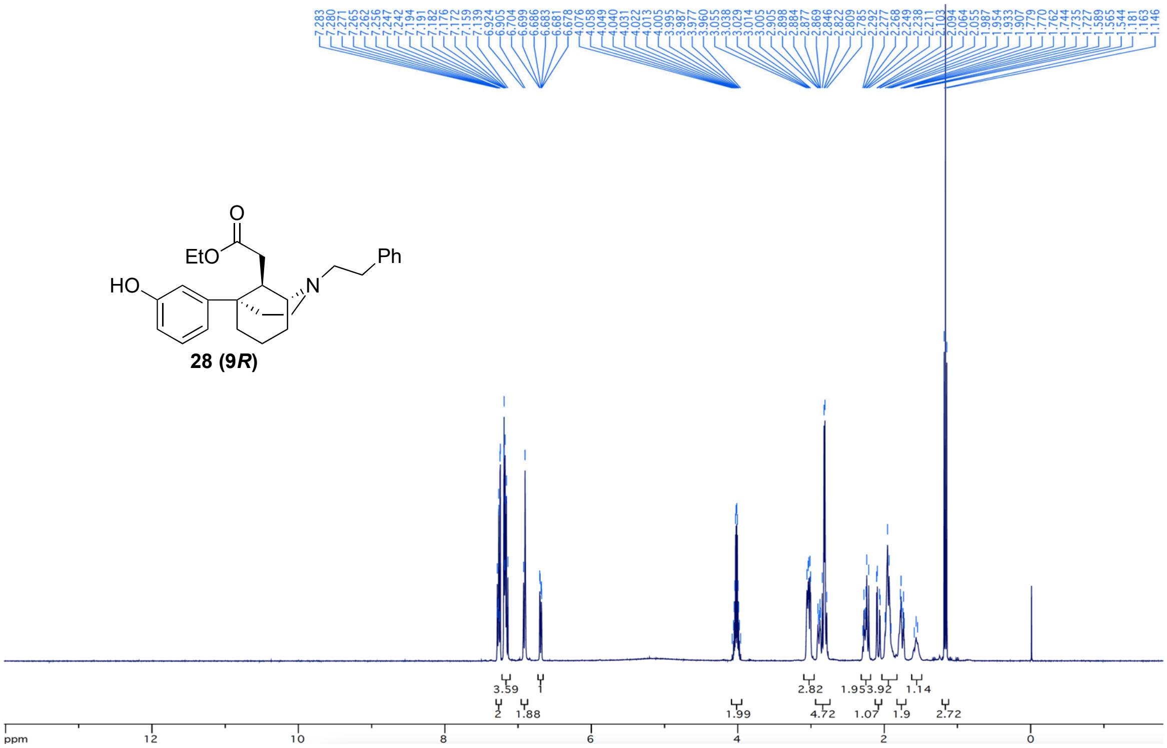

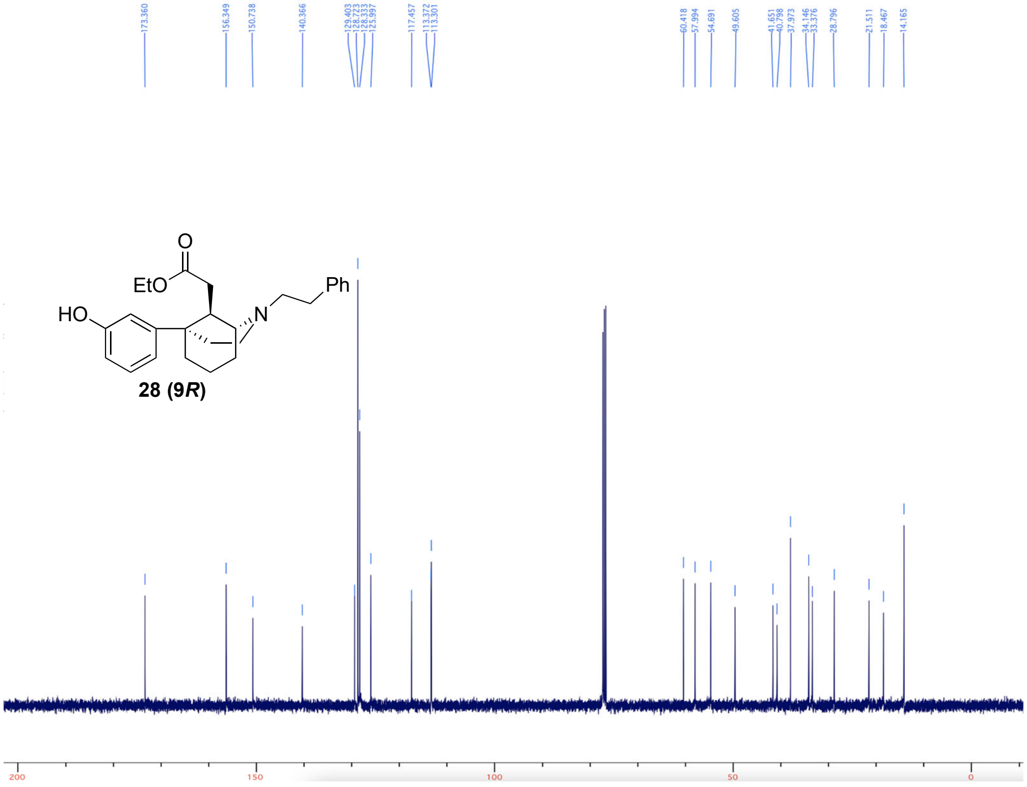

Figure S17: ^{1}H and ^{13}C-NMR spectra of compound **28**

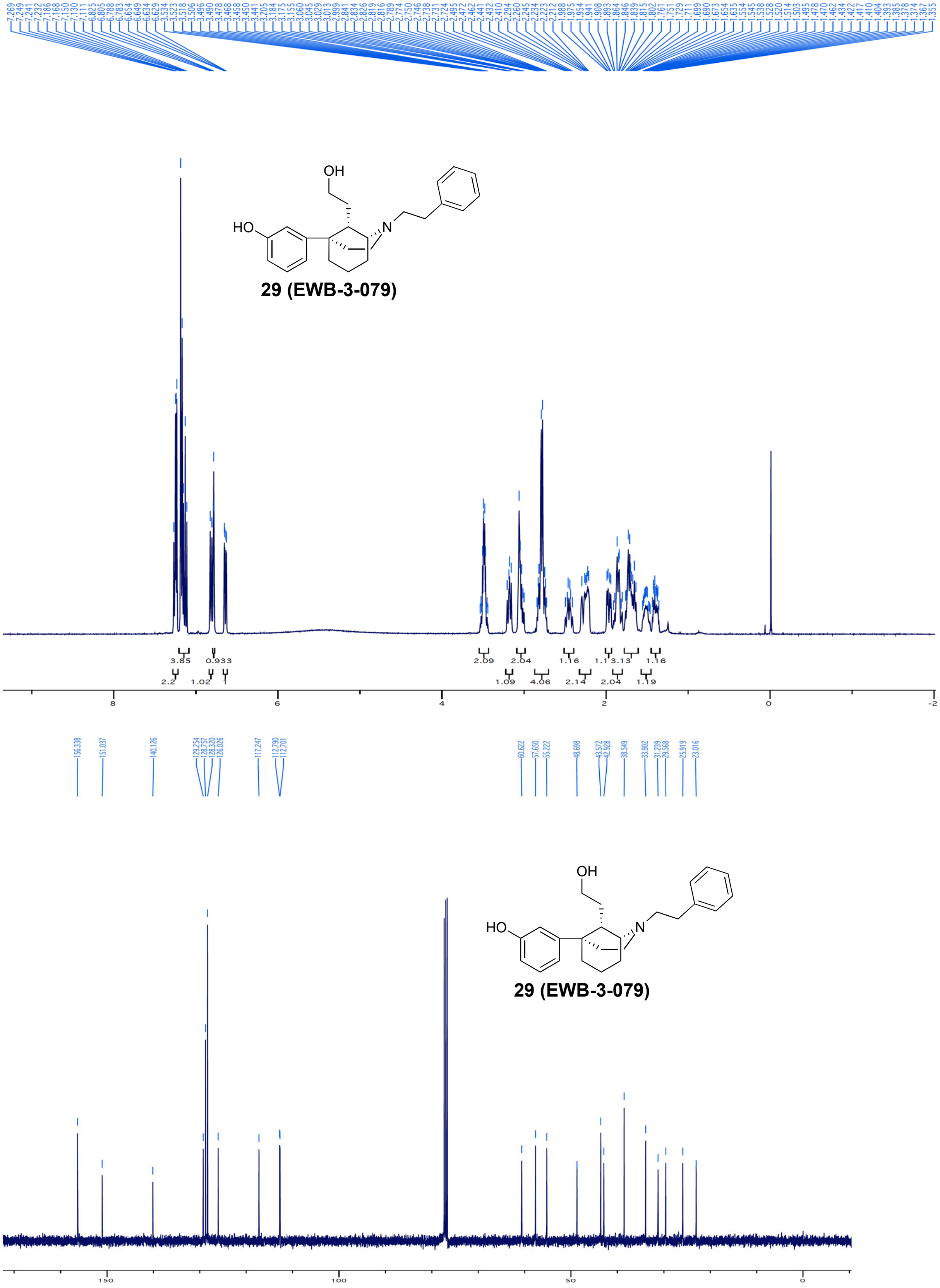

Figure S18: ^{1}H and ^{13}C-NMR spectra of compound **29**

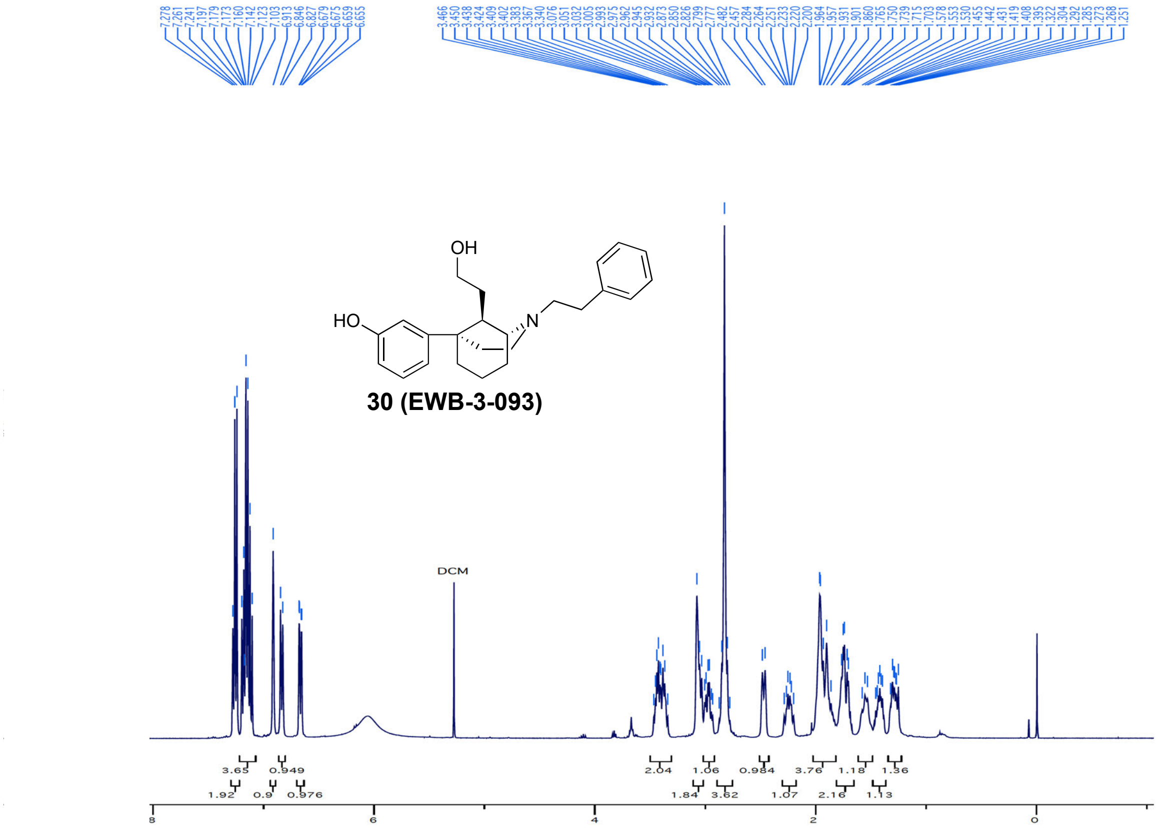

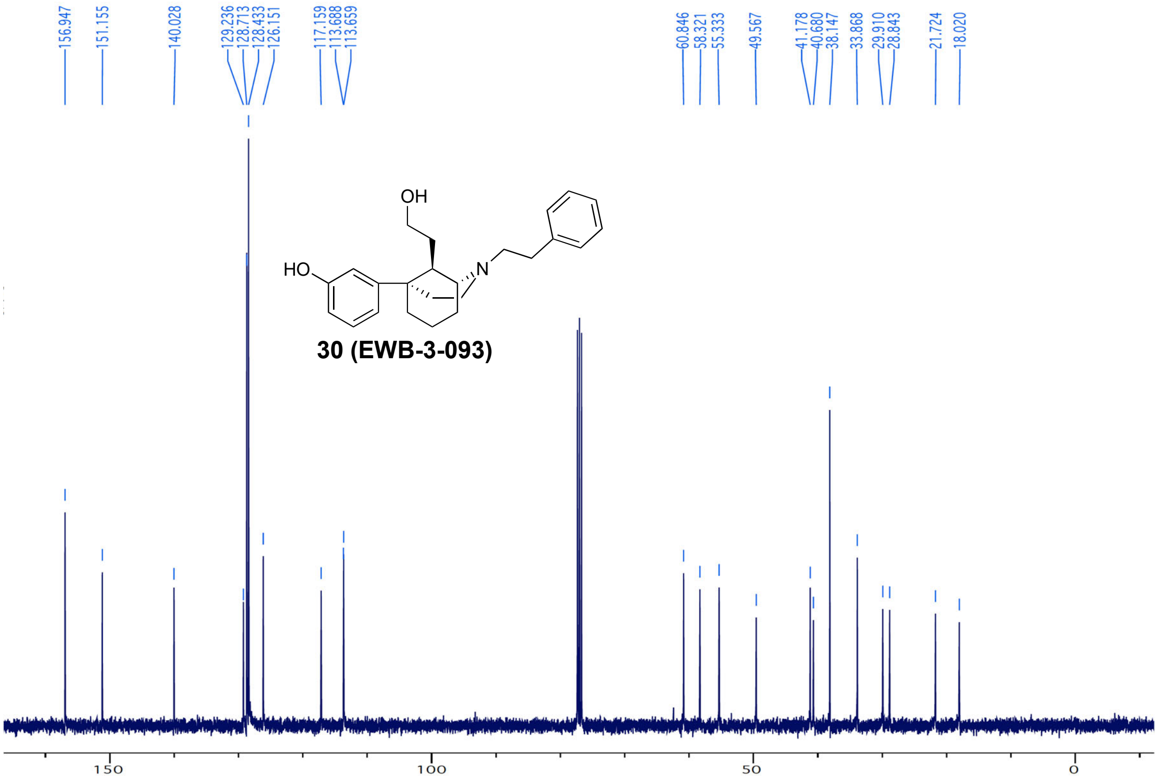

Figure S19: ^{1}H and ^{13}C-NMR spectra of compound **30**

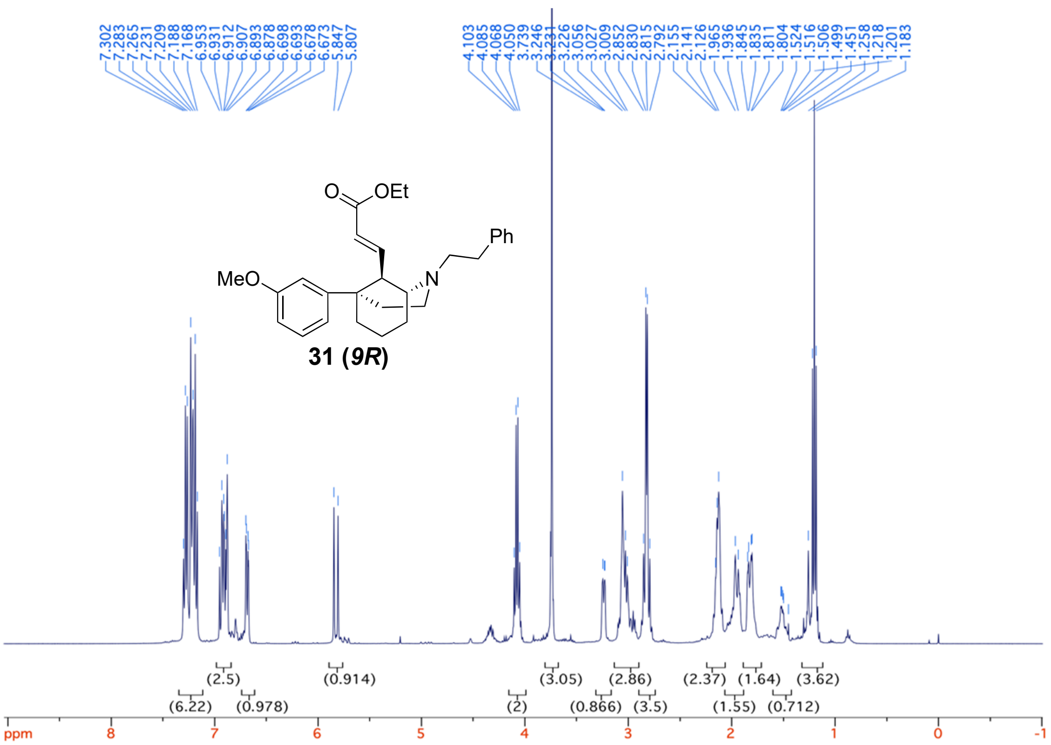

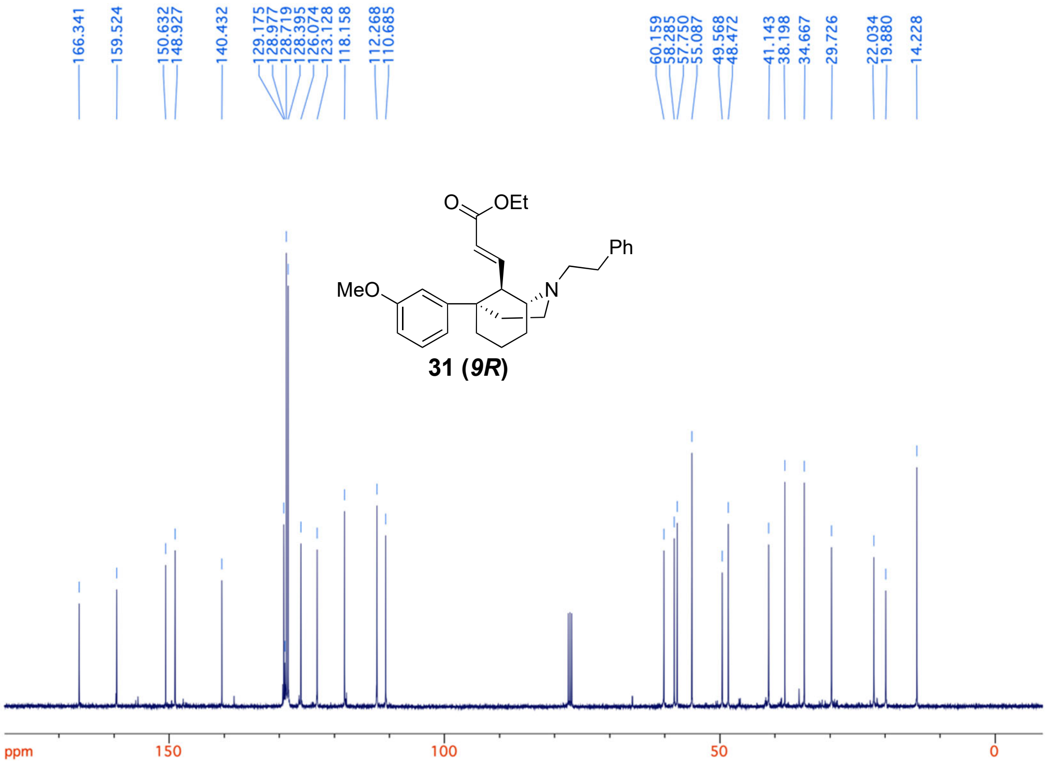

Figure S20: ^{1}H and ^{13}C-NMR spectra of compound **31**

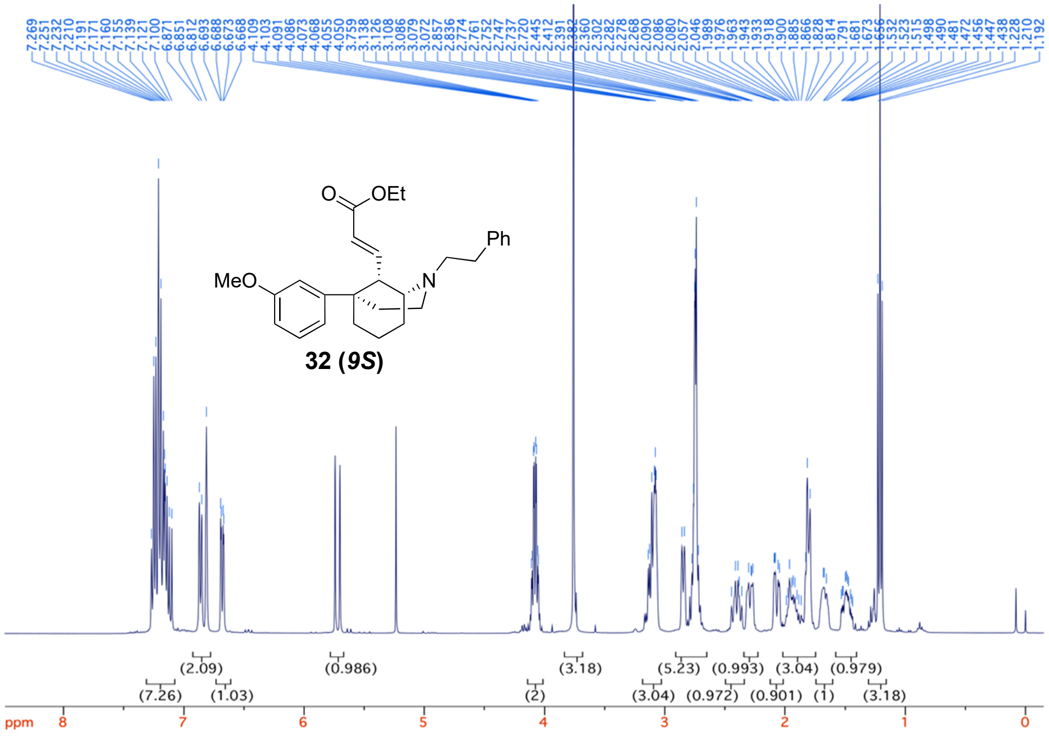

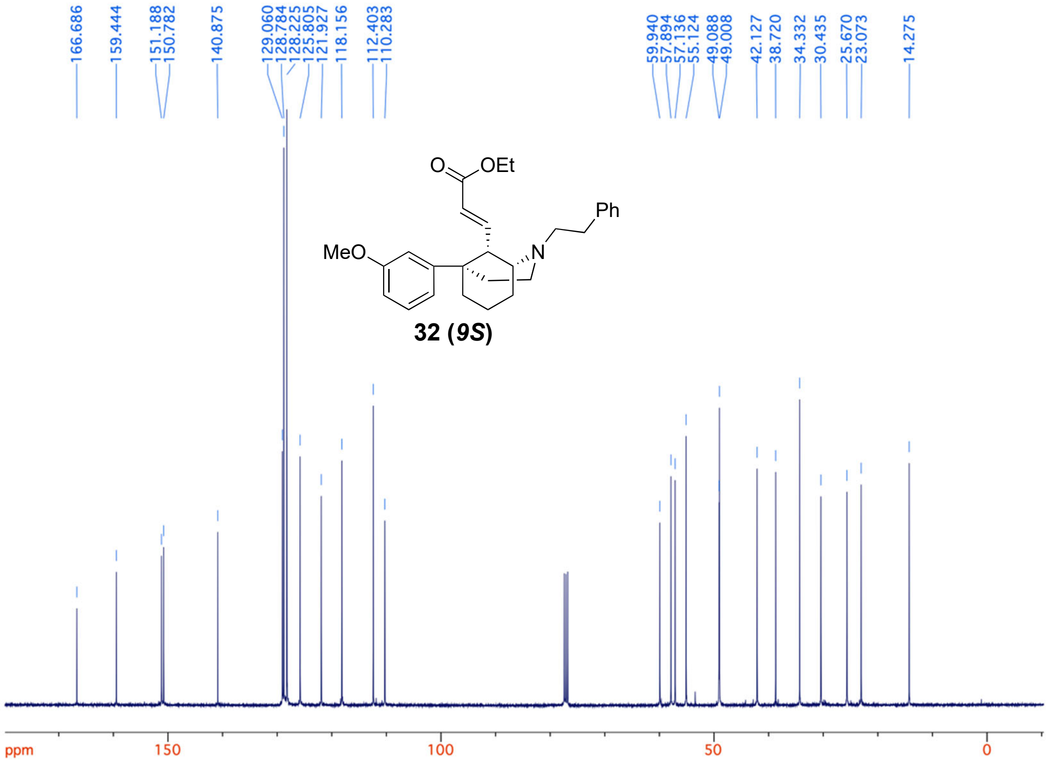

Figure S21: ^{1}H and ^{13}C-NMR spectra of compound **32**

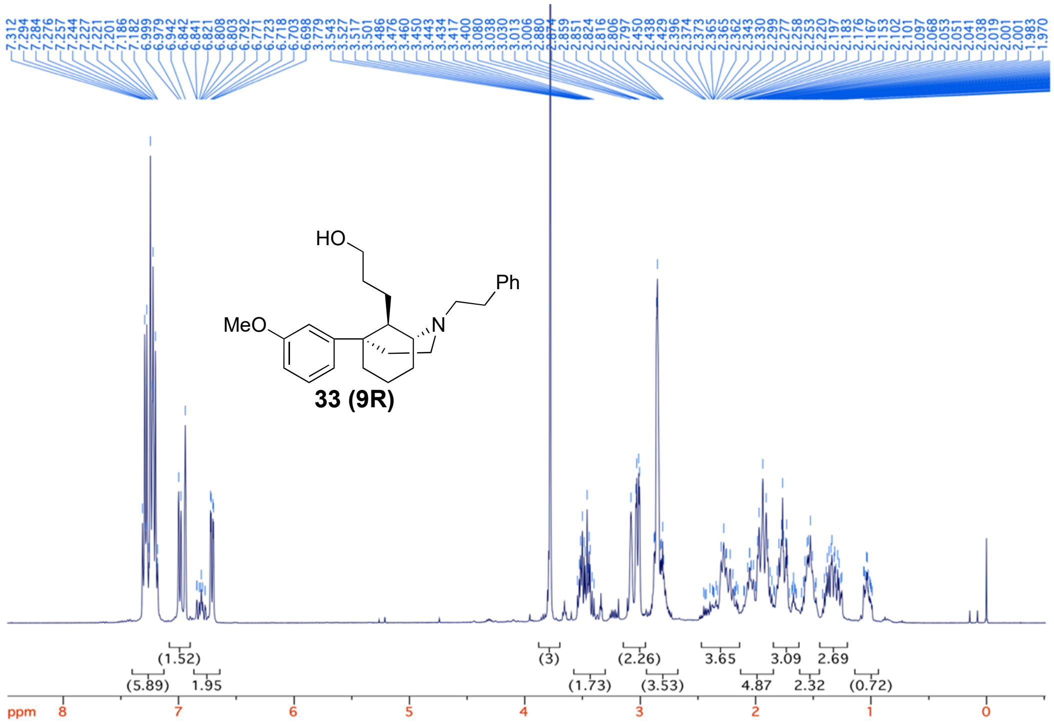

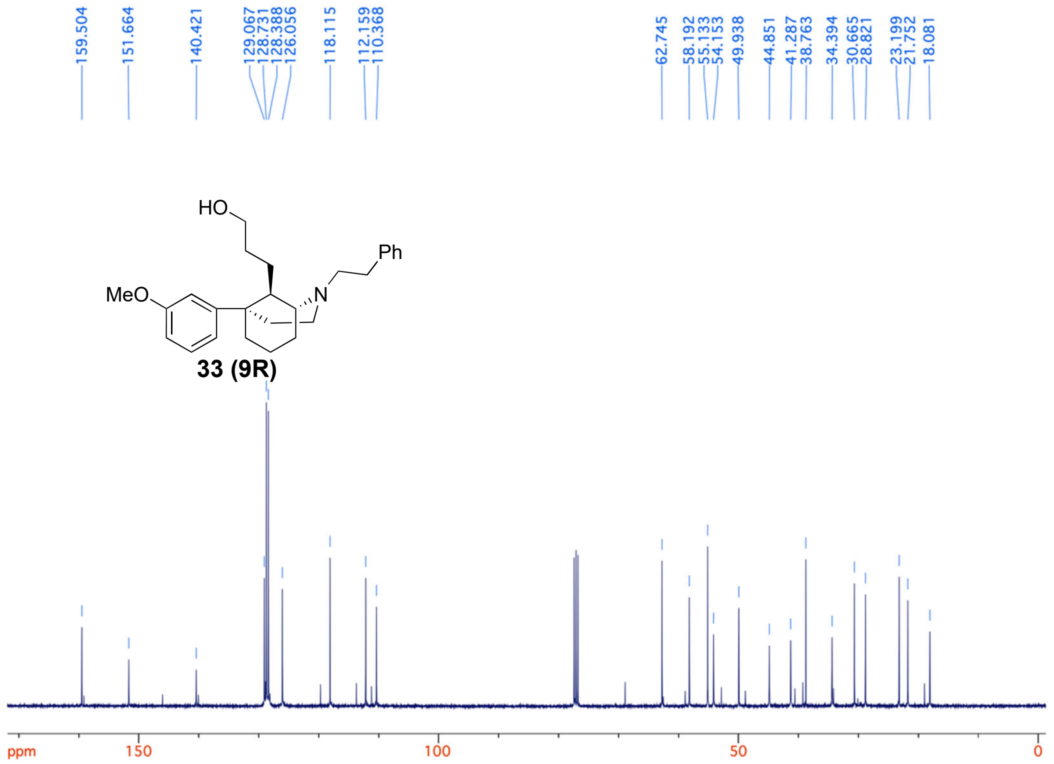

Figure S21: ^{1}H and ^{13}C-NMR spectra of compound **33**

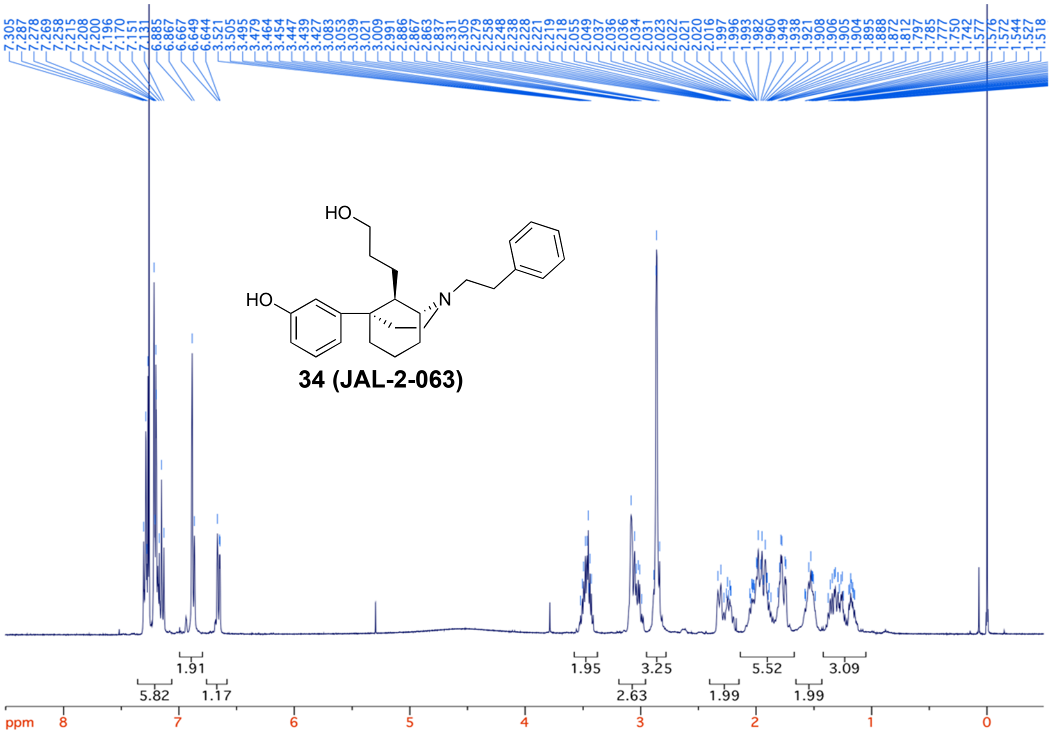

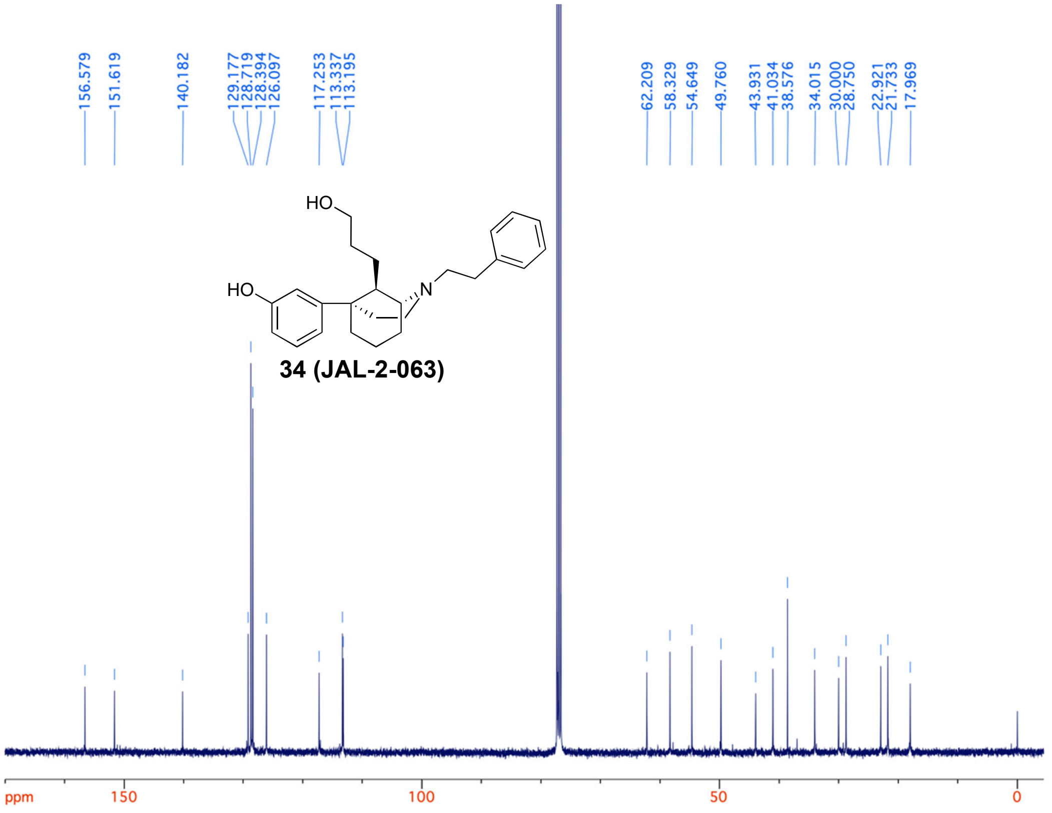

Figure S22: ^{1}H and ^{13}C-NMR spectra of compound **34**

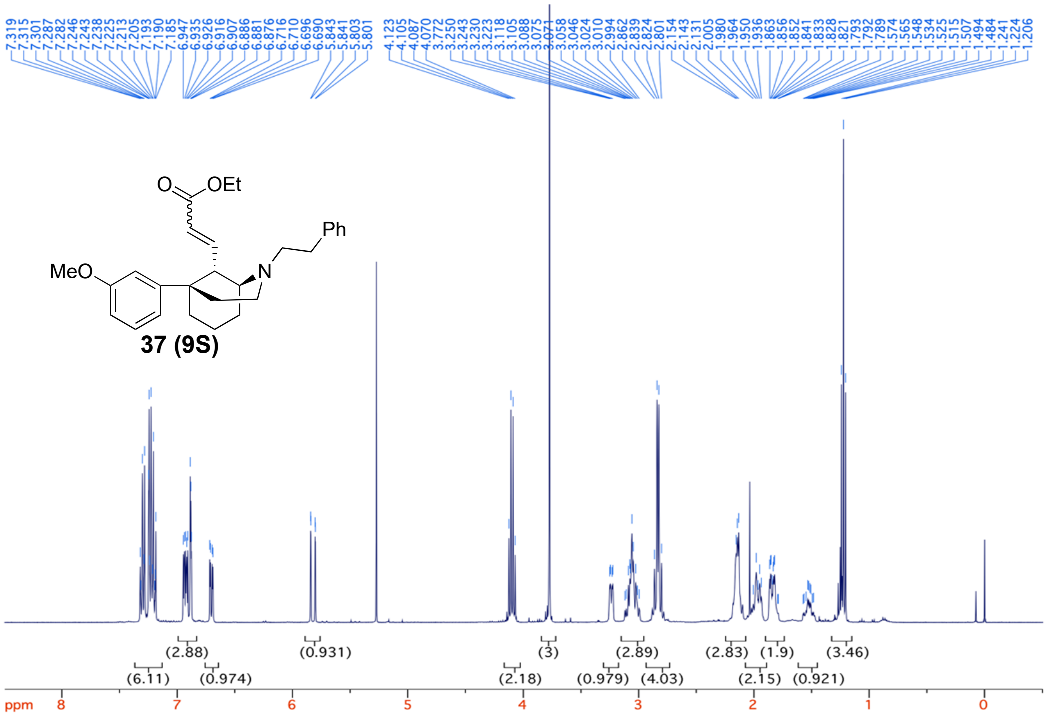

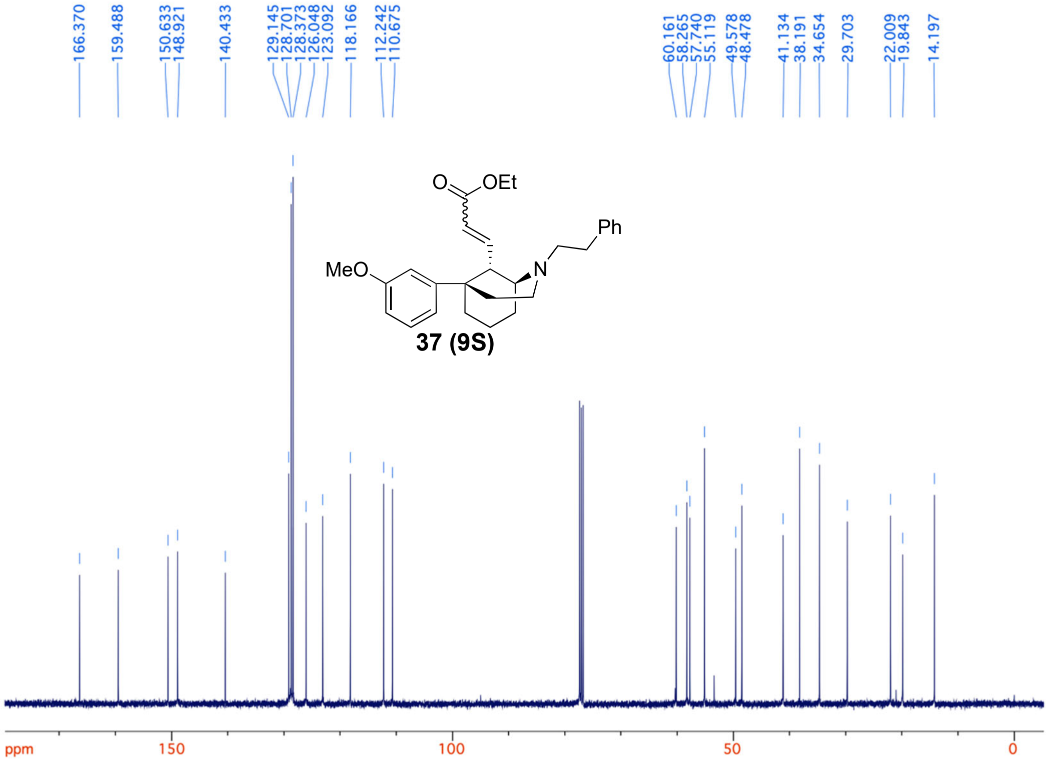

Figure S23: ^{1}H and ^{13}C-NMR spectra of compound **37**

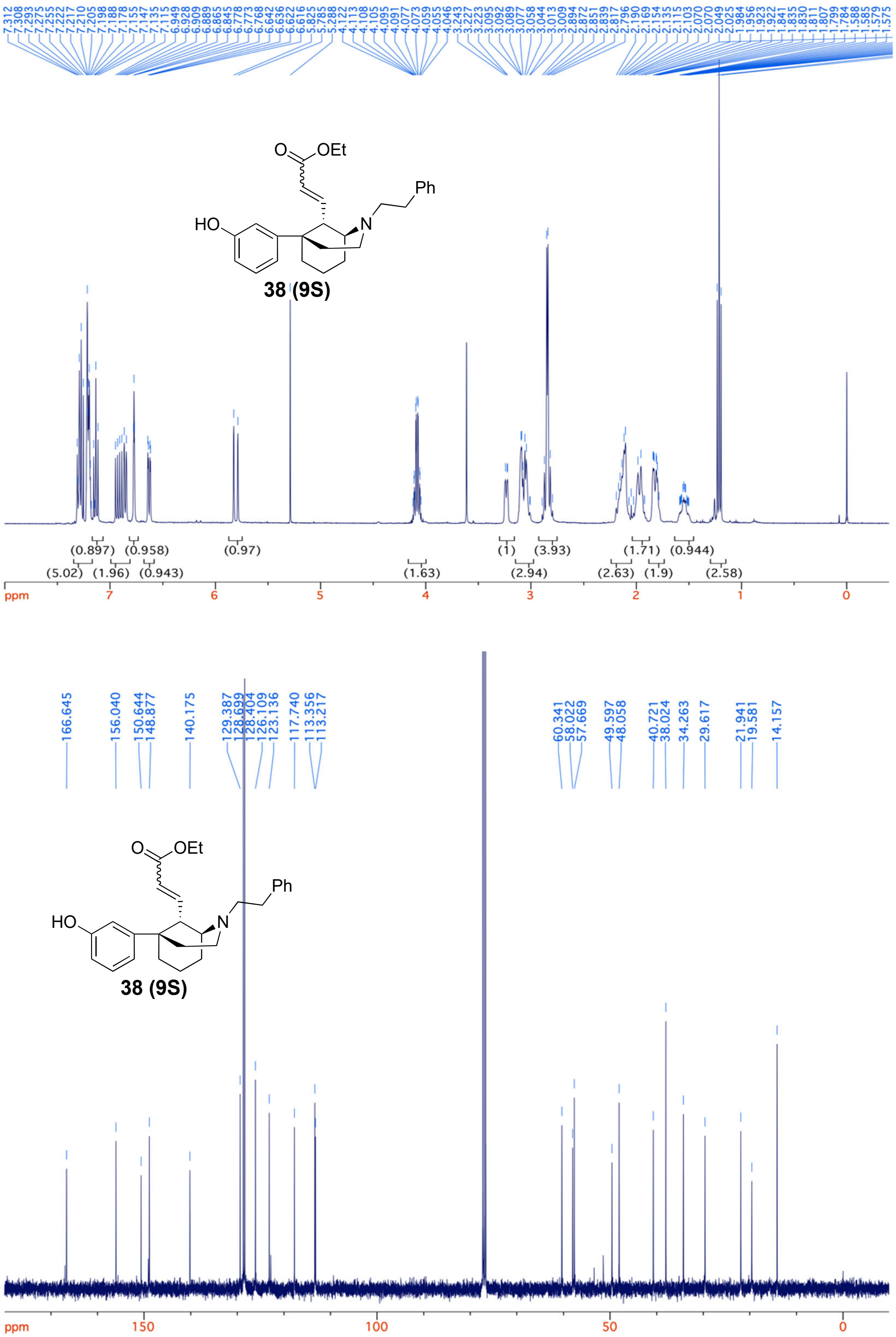

Figure S24: ^{1}H and ^{13}C-NMR spectra of compound **38**

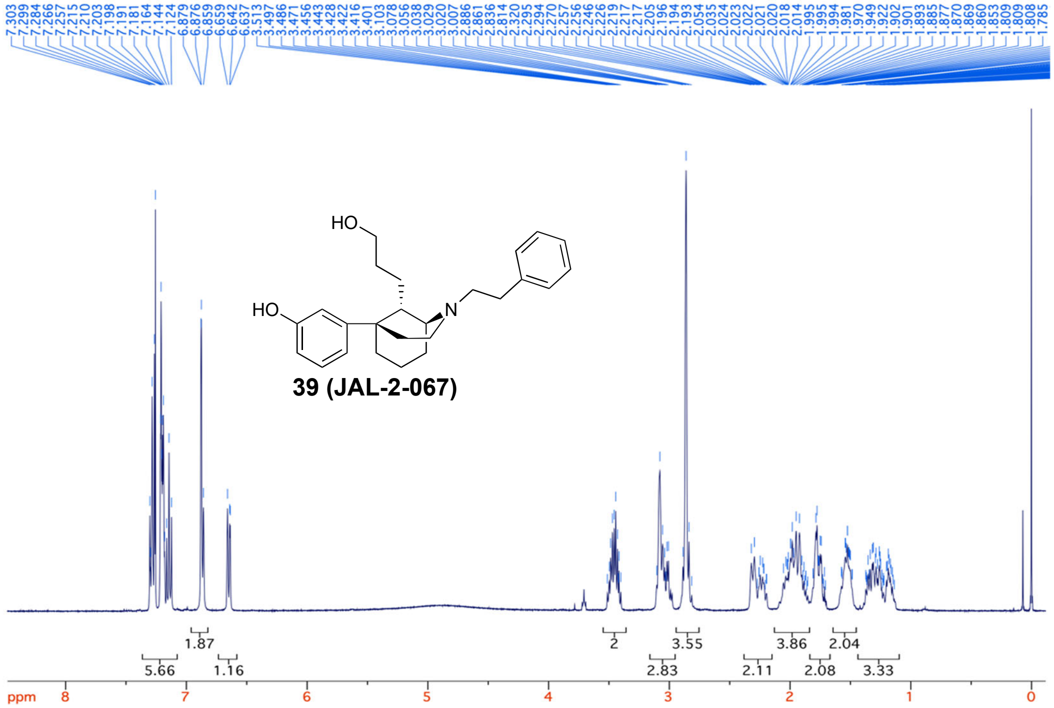

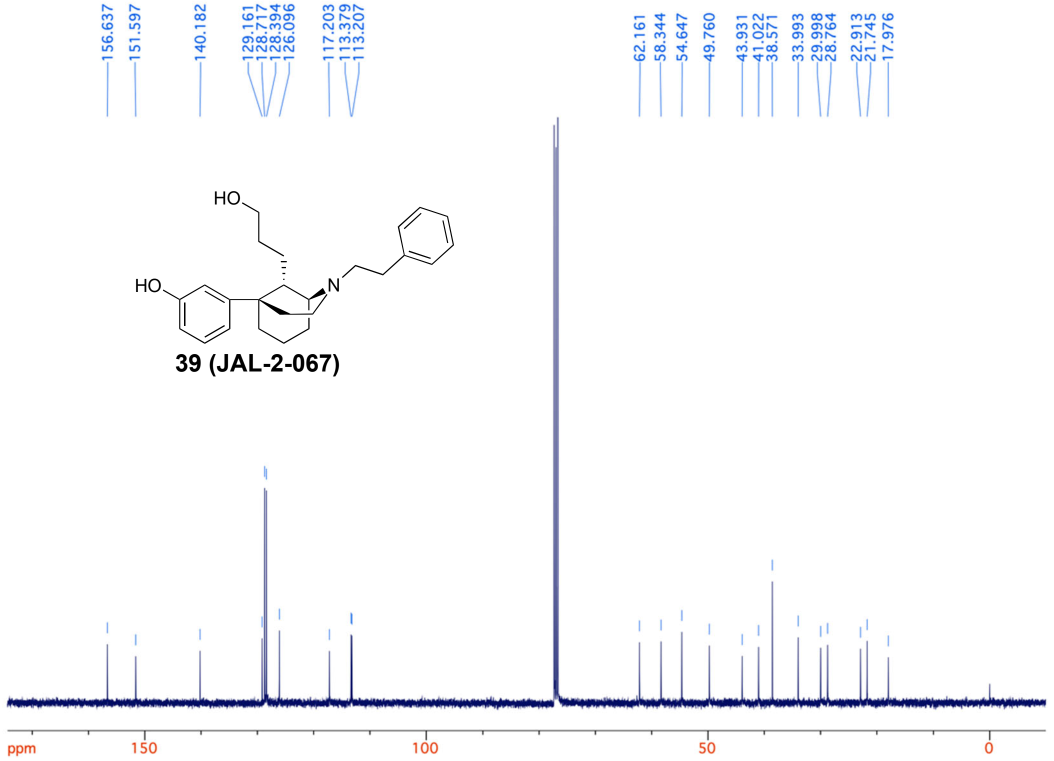

Figure S25: ^{1}H and ^{13}C-NMR spectra of compound **39**

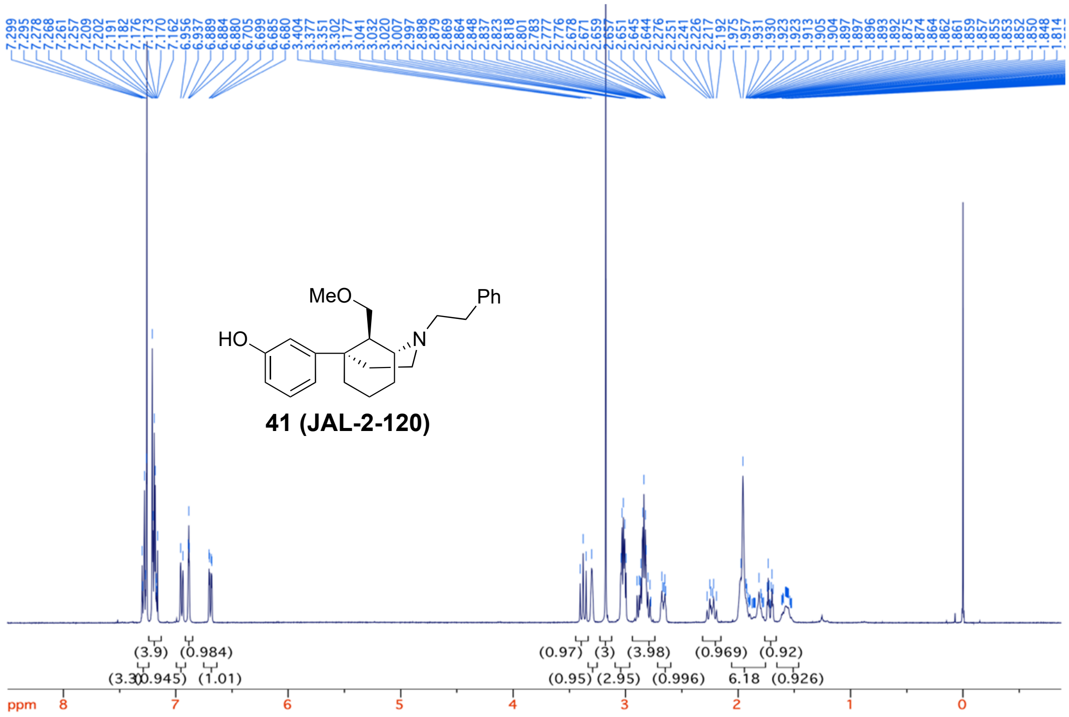

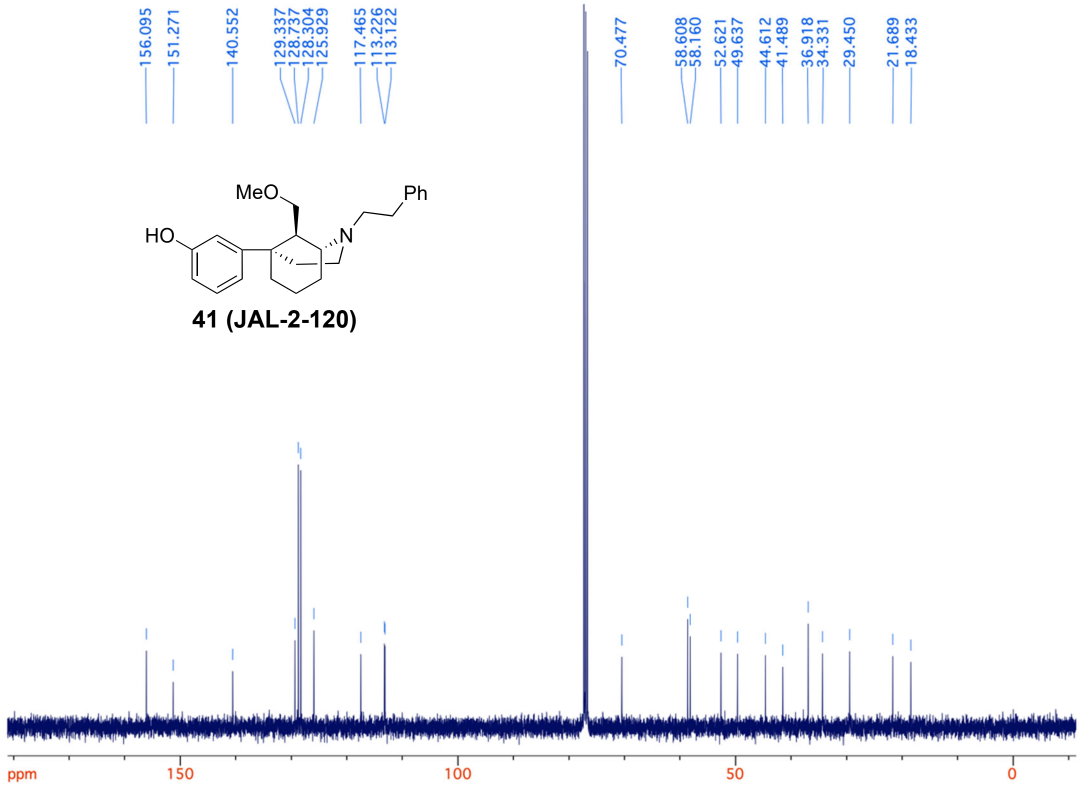

Figure S26: ^{1}H and ^{13}C-NMR spectra of compound **41**

Table S1. Crystal data and structure refinement for **15** (knih122).

Identification code	shelx
Empirical formula	C23 H30 Br N O2
Formula weight	432.39
Temperature	296(2) K
Wavelength	0.71073 Å
Crystal system	Orthorhombic
Space group	P 21 21 21
Unit cell dimensions	a = 7.24780(10) Å<br>a= 90°.<br>b = 14.8829(3) Å<br>b= 90°.<br>c = 19.7753(4) Å<br>g = 90°.
Volume	2133.13(7) $Å^3$
Z	4
Density (calculated)	1.346 Mg/m^3
Absorption coefficient	1.944 mm^{-1}
F(000)	904
Crystal size	0.409 x 0.293 x 0.219 mm^3
Theta range for data collection	1.712 to 29.163°.
Index ranges	-9<=h<=9, -20<=k<=20, -25<=l<=26
Reflections collected	24788
Independent reflections	5693 [R(int) = 0.0214]
Completeness to theta = 25.000°	100.00%
Absorption correction	Semi-empirical from equivalents
Max. and min. transmission	0.7458 and 0.6341
Refinement method	Full-matrix least-squares on F^2
Data / restraints / parameters	5693 / 0 / 244
Goodness-of-fit on F^2	1.039
Final R indices [I>2sigma(I)]	R1 = 0.0336, wR2 = 0.0906
R indices (all data)	R1 = 0.0411, wR2 = 0.0940
Absolute structure parameter	0.010(3)
Extinction coefficient	n/a
Largest diff. peak and hole	0.674 and -0.343 e.$Å^{-3}$

Table S2. Atomic coordinates (x 10^4) and equivalent isotropic displacement parameters ($Å^2$x 10^3) for **15** (knih122). U(eq) is defined as one third of the trace of the orthogonalized U^{ij} tensor.

	x	y	z	U(eq)
Br(1)	6328(1)	9789(1)	2545(1)	47(1)
C(1)	1639(3)	8042(2)	2700(1)	32(1)
N(2)	3719(3)	8021(2)	2792(1)	31(1)
C(3)	4331(4)	7747(2)	3487(2)	39(1)
C(4)	3251(4)	8202(2)	4051(1)	36(1)
C(5)	1153(4)	8253(2)	3948(1)	29(1)
C(6)	232(5)	7311(2)	3978(2)	39(1)
C(7)	709(5)	6664(2)	3402(2)	47(1)
C(8)	750(4)	7103(2)	2711(2)	42(1)
C(9)	793(4)	8651(2)	3238(1)	30(1)
C(9A)	1342(5)	9634(2)	3149(2)	40(1)
O(9)	973(4)	9924(2)	2485(1)	65(1)
C(10)	261(4)	8808(2)	4513(1)	32(1)
C(11)	1119(5)	8913(2)	5138(1)	41(1)
C(12)	254(5)	9373(2)	5666(2)	47(1)
O(12)	1194(6)	9419(2)	6251(1)	82(1)
C(13)	-1466(6)	9735(2)	5580(2)	53(1)
C(14)	-2339(5)	9622(2)	4966(2)	56(1)
C(15)	-1503(5)	9175(2)	4435(2)	46(1)
C(16)	4773(4)	7456(2)	2289(2)	39(1)
C(17)	4437(6)	7714(3)	1560(2)	48(1)
C(18)	5950(5)	7324(2)	1128(2)	48(1)
C(19)	5698(8)	6500(3)	817(2)	72(1)
C(20)	7075(11)	6123(4)	435(3)	100(2)
C(21)	8690(11)	6530(4)	365(3)	100(2)
C(22)	9000(8)	7351(5)	666(3)	94(2)
C(23)	7617(7)	7759(3)	1063(2)	69(1)

Table S3. Bond lengths [Å] and angles [°] for **15** (knih122).

C(1)-N(2)	1.519(3)
C(1)-C(9)	1.525(4)
C(1)-C(8)	1.538(4)
C(1)-H(1A)	0.98
N(2)-C(3)	1.499(4)
N(2)-C(16)	1.510(3)
N(2)-H(2A)	0.98
C(3)-C(4)	1.522(4)
C(3)-H(3A)	0.97
C(3)-H(3B)	0.97
C(4)-C(5)	1.536(4)
C(4)-H(4A)	0.97
C(4)-H(4B)	0.97
C(5)-C(10)	1.532(4)
C(5)-C(9)	1.547(3)
C(5)-C(6)	1.554(4)
C(6)-C(7)	1.531(4)
C(6)-H(6A)	0.97
C(6)-H(6B)	0.97
C(7)-C(8)	1.516(5)
C(7)-H(7A)	0.97
C(7)-H(7B)	0.97
C(8)-H(8A)	0.97
C(8)-H(8B)	0.97
C(9)-C(9A)	1.526(4)
C(9)-H(9A)	0.98
C(9A)-O(9)	1.408(4)
C(9A)-H(9AA)	0.97
C(9A)-H(9AB)	0.97
O(9)-H(9B)	0.82
C(10)-C(11)	1.392(4)
C(10)-C(15)	1.399(4)
C(11)-C(12)	1.397(4)
C(11)-H(11A)	0.93
C(12)-O(12)	1.344(5)
C(12)-C(13)	1.368(6)
O(12)-H(12A)	0.82
C(13)-C(14)	1.380(5)
C(13)-H(13A)	0.93
C(14)-C(15)	1.384(4)
C(14)-H(14A)	0.93
C(15)-H(15A)	0.93
C(16)-C(17)	1.511(4)
C(16)-H(16A)	0.97
C(16)-H(16B)	0.97
C(17)-C(18)	1.506(5)

C(17)-H(17A)	0.97
C(17)-H(17B)	0.97
C(18)-C(23)	1.376(6)
C(18)-C(19)	1.385(6)
C(19)-C(20)	1.371(7)
C(19)-H(19A)	0.93
C(20)-C(21)	1.325(10)
C(20)-H(20A)	0.93
C(21)-C(22)	1.378(9)
C(21)-H(21A)	0.93
C(22)-C(23)	1.410(7)
C(22)-H(22A)	0.93
C(23)-H(23A)	0.93
N(2)-C(1)-C(9)	109.1(2)
N(2)-C(1)-C(8)	113.2(2)
C(9)-C(1)-C(8)	111.2(2)
N(2)-C(1)-H(1A)	107.7
C(9)-C(1)-H(1A)	107.7
C(8)-C(1)-H(1A)	107.7
C(3)-N(2)-C(16)	107.7(2)
C(3)-N(2)-C(1)	114.1(2)
C(16)-N(2)-C(1)	115.8(2)
C(3)-N(2)-H(2A)	106.2
C(16)-N(2)-H(2A)	106.2
C(1)-N(2)-H(2A)	106.2
N(2)-C(3)-C(4)	113.5(2)
N(2)-C(3)-H(3A)	108.9
C(4)-C(3)-H(3A)	108.9
N(2)-C(3)-H(3B)	108.9
C(4)-C(3)-H(3B)	108.9
H(3A)-C(3)-H(3B)	107.7
C(3)-C(4)-C(5)	115.7(2)
C(3)-C(4)-H(4A)	108.4
C(5)-C(4)-H(4A)	108.4
C(3)-C(4)-H(4B)	108.4
C(5)-C(4)-H(4B)	108.4
H(4A)-C(4)-H(4B)	107.4
C(10)-C(5)-C(4)	110.3(2)
C(10)-C(5)-C(9)	112.6(2)
C(4)-C(5)-C(9)	107.8(2)
C(10)-C(5)-C(6)	106.1(2)
C(4)-C(5)-C(6)	112.1(2)
C(9)-C(5)-C(6)	107.9(2)
C(7)-C(6)-C(5)	116.2(2)
C(7)-C(6)-H(6A)	108.2
C(5)-C(6)-H(6A)	108.2
C(7)-C(6)-H(6B)	108.2

C(5)-C(6)-H(6B)	108.2
H(6A)-C(6)-H(6B)	107.4
C(8)-C(7)-C(6)	113.8(3)
C(8)-C(7)-H(7A)	108.8
C(6)-C(7)-H(7A)	108.8
C(8)-C(7)-H(7B)	108.8
C(6)-C(7)-H(7B)	108.8
H(7A)-C(7)-H(7B)	107.7
C(7)-C(8)-C(1)	114.4(2)
C(7)-C(8)-H(8A)	108.7
C(1)-C(8)-H(8A)	108.7
C(7)-C(8)-H(8B)	108.7
C(1)-C(8)-H(8B)	108.7
H(8A)-C(8)-H(8B)	107.6
C(1)-C(9)-C(9A)	112.7(2)
C(1)-C(9)-C(5)	109.7(2)
C(9A)-C(9)-C(5)	115.3(2)
C(1)-C(9)-H(9A)	106.2
C(9A)-C(9)-H(9A)	106.2
C(5)-C(9)-H(9A)	106.2
O(9)-C(9A)-C(9)	110.5(3)
O(9)-C(9A)-H(9AA)	109.5
C(9)-C(9A)-H(9AA)	109.5
O(9)-C(9A)-H(9AB)	109.5
C(9)-C(9A)-H(9AB)	109.5
H(9AA)-C(9A)-H(9AB)	108.1
C(9A)-O(9)-H(9B)	109.5
C(11)-C(10)-C(15)	117.6(3)
C(11)-C(10)-C(5)	121.3(3)
C(15)-C(10)-C(5)	121.0(3)
C(10)-C(11)-C(12)	121.2(3)
C(10)-C(11)-H(11A)	119.4
C(12)-C(11)-H(11A)	119.4
O(12)-C(12)-C(13)	123.2(3)
O(12)-C(12)-C(11)	116.2(3)
C(13)-C(12)-C(11)	120.6(3)
C(12)-O(12)-H(12A)	109.5
C(12)-C(13)-C(14)	118.6(3)
C(12)-C(13)-H(13A)	120.7
C(14)-C(13)-H(13A)	120.7
C(13)-C(14)-C(15)	121.7(3)
C(13)-C(14)-H(14A)	119.1
C(15)-C(14)-H(14A)	119.1
C(14)-C(15)-C(10)	120.3(3)
C(14)-C(15)-H(15A)	119.9
C(10)-C(15)-H(15A)	119.9
C(17)-C(16)-N(2)	114.0(2)

C(17)-C(16)-H(16A)	108.8
N(2)-C(16)-H(16A)	108.8
C(17)-C(16)-H(16B)	108.8
N(2)-C(16)-H(16B)	108.8
H(16A)-C(16)-H(16B)	107.7
C(18)-C(17)-C(16)	109.0(3)
C(18)-C(17)-H(17A)	109.9
C(16)-C(17)-H(17A)	109.9
C(18)-C(17)-H(17B)	109.9
C(16)-C(17)-H(17B)	109.9
H(17A)-C(17)-H(17B)	108.3
C(23)-C(18)-C(19)	119.3(4)
C(23)-C(18)-C(17)	120.7(4)
C(19)-C(18)-C(17)	119.9(4)
C(20)-C(19)-C(18)	120.8(5)
C(20)-C(19)-H(19A)	119.6
C(18)-C(19)-H(19A)	119.6
C(21)-C(20)-C(19)	120.9(6)
C(21)-C(20)-H(20A)	119.6
C(19)-C(20)-H(20A)	119.6
C(20)-C(21)-C(22)	120.3(5)
C(20)-C(21)-H(21A)	119.9
C(22)-C(21)-H(21A)	119.9
C(21)-C(22)-C(23)	120.4(6)
C(21)-C(22)-H(22A)	119.8
C(23)-C(22)-H(22A)	119.8
C(18)-C(23)-C(22)	118.3(5)
C(18)-C(23)-H(23A)	120.9
C(22)-C(23)-H(23A)	120.9

Symmetry transformations used to generate equivalent atoms:

Table S4. Anisotropic displacement parameters (Å^{2}x 10^3) for **15** (knih122). The anisotropic displacement factor exponent takes the form: -2p^2[$h^2a^{*2}U^{11}$ + ... + 2 h k a* b* U^{12}].

	U_{11}	U_{22}	U_{33}	U_{23}	U_{13}	U_{12}
Br(1)	41(1)	49(1)	52(1)	13(1)	-6(1)	-7(1)
C(1)	29(1)	41(1)	27(1)	3(1)	-2(1)	6(1)
N(2)	28(1)	34(1)	30(1)	0(1)	3(1)	2(1)
C(3)	30(2)	52(2)	34(1)	1(1)	-2(1)	11(1)
C(4)	32(2)	49(2)	29(1)	-1(1)	-4(1)	7(1)
C(5)	30(1)	30(1)	26(1)	3(1)	2(1)	4(1)
C(6)	47(2)	31(1)	38(2)	4(1)	8(1)	-1(1)
C(7)	58(2)	34(1)	50(2)	-5(1)	9(2)	-8(1)
C(8)	36(2)	49(2)	41(2)	-12(1)	1(1)	-7(1)
C(9)	26(1)	34(1)	29(1)	6(1)	1(1)	7(1)
C(9A)	43(2)	34(1)	44(1)	10(1)	11(1)	8(1)
O(9)	79(2)	59(1)	57(2)	34(1)	10(2)	12(1)
C(10)	37(1)	30(1)	31(1)	3(1)	8(1)	1(1)
C(11)	54(2)	39(1)	29(1)	2(1)	5(1)	8(1)
C(12)	70(2)	39(2)	31(2)	0(1)	12(2)	-1(2)
O(12)	109(3)	95(2)	41(1)	-22(1)	2(2)	9(2)
C(13)	76(2)	38(2)	45(2)	-1(1)	28(2)	7(2)
C(14)	46(2)	54(2)	66(2)	0(2)	20(2)	13(2)
C(15)	41(2)	52(2)	44(2)	-3(1)	6(1)	7(1)
C(16)	36(1)	42(1)	38(2)	-2(1)	6(1)	11(1)
C(17)	50(2)	58(2)	34(2)	-4(1)	5(1)	15(2)
C(18)	60(2)	51(2)	34(2)	4(1)	11(2)	14(2)
C(19)	95(4)	61(2)	60(2)	-13(2)	19(2)	12(2)
C(20)	140(6)	76(3)	83(4)	-9(3)	41(4)	38(4)
C(21)	121(5)	110(4)	70(3)	9(3)	38(4)	69(4)
C(22)	70(3)	125(5)	86(3)	36(3)	32(3)	15(3)
C(23)	66(3)	74(3)	68(3)	11(2)	20(2)	8(2)

Table S5. Hydrogen coordinates (x 10^4) and isotropic displacement parameters ($Å^2$x 10^3) for **15** (knih122).

	x	y	z	U(eq)
H(1A)	1383	8311	2258	39
H(2A)	4147	8640	2729	37
H(3A)	5629	7890	3538	46
H(3B)	4198	7101	3531	46
H(4A)	3720	8808	4105	44
H(4B)	3492	7882	4469	44
H(6A)	576	7029	4402	46
H(6B)	-1096	7393	3984	46
H(7A)	1908	6399	3492	57
H(7B)	-190	6181	3396	57
H(8A)	-504	7152	2544	50
H(8B)	1423	6717	2403	50
H(9A)	-545	8626	3169	36
H(9AA)	660	10003	3467	48
H(9AB)	2647	9704	3245	48
H(9B)	1275	10452	2445	97
H(11A)	2289	8672	5205	49
H(12A)	586	9698	6530	123
H(13A)	-2034	10050	5929	63
H(14A)	-3522	9852	4908	67
H(15A)	-2116	9118	4024	55
H(16A)	6081	7509	2384	46
H(16B)	4431	6832	2350	46
H(17A)	3250	7486	1413	57
H(17B)	4424	8363	1516	57
H(19A)	4582	6199	867	86
H(20A)	6874	5573	223	120
H(21A)	9618	6260	111	121
H(22A)	10128	7639	607	113
H(23A)	7827	8306	1276	83

Table S6. Torsion angles [°] for **15** (knih122).

C(9)-C(1)-N(2)-C(3)	55.2(3)
C(8)-C(1)-N(2)-C(3)	-69.3(3)
C(9)-C(1)-N(2)-C(16)	-178.9(2)
C(8)-C(1)-N(2)-C(16)	56.6(3)
C(16)-N(2)-C(3)-C(4)	-174.7(3)
C(1)-N(2)-C(3)-C(4)	-44.7(3)
N(2)-C(3)-C(4)-C(5)	43.4(4)
C(3)-C(4)-C(5)-C(10)	-174.2(2)
C(3)-C(4)-C(5)-C(9)	-50.9(3)
C(3)-C(4)-C(5)-C(6)	67.8(3)
C(10)-C(5)-C(6)-C(7)	172.3(3)
C(4)-C(5)-C(6)-C(7)	-67.2(3)
C(9)-C(5)-C(6)-C(7)	51.4(3)
C(5)-C(6)-C(7)-C(8)	-41.9(4)
C(6)-C(7)-C(8)-C(1)	40.6(4)
N(2)-C(1)-C(8)-C(7)	71.9(3)
C(9)-C(1)-C(8)-C(7)	-51.5(3)
N(2)-C(1)-C(9)-C(9A)	66.2(3)
C(8)-C(1)-C(9)-C(9A)	-168.1(2)
N(2)-C(1)-C(9)-C(5)	-63.7(3)
C(8)-C(1)-C(9)-C(5)	62.0(3)
C(10)-C(5)-C(9)-C(1)	-177.3(2)
C(4)-C(5)-C(9)-C(1)	60.8(3)
C(6)-C(5)-C(9)-C(1)	-60.5(3)
C(10)-C(5)-C(9)-C(9A)	54.3(3)
C(4)-C(5)-C(9)-C(9A)	-67.7(3)
C(6)-C(5)-C(9)-C(9A)	171.0(2)
C(1)-C(9)-C(9A)-O(9)	52.5(3)
C(5)-C(9)-C(9A)-O(9)	179.4(2)
C(4)-C(5)-C(10)-C(11)	-23.5(3)
C(9)-C(5)-C(10)-C(11)	-144.0(3)
C(6)-C(5)-C(10)-C(11)	98.1(3)
C(4)-C(5)-C(10)-C(15)	160.9(3)
C(9)-C(5)-C(10)-C(15)	40.4(3)
C(6)-C(5)-C(10)-C(15)	-77.4(3)
C(15)-C(10)-C(11)-C(12)	-0.4(4)
C(5)-C(10)-C(11)-C(12)	-176.1(3)
C(10)-C(11)-C(12)-O(12)	178.7(3)
C(10)-C(11)-C(12)-C(13)	-0.1(5)

O(12)-C(12)-C(13)-C(14)	-177.6(3)
C(11)-C(12)-C(13)-C(14)	1.1(5)
C(12)-C(13)-C(14)-C(15)	-1.7(5)
C(13)-C(14)-C(15)-C(10)	1.2(5)
C(11)-C(10)-C(15)-C(14)	-0.1(5)
C(5)-C(10)-C(15)-C(14)	175.6(3)
C(3)-N(2)-C(16)-C(17)	-174.6(3)
C(1)-N(2)-C(16)-C(17)	56.4(4)
N(2)-C(16)-C(17)-C(18)	162.5(3)
C(16)-C(17)-C(18)-C(23)	-82.9(4)
C(16)-C(17)-C(18)-C(19)	94.0(4)
C(23)-C(18)-C(19)-C(20)	-1.3(7)
C(17)-C(18)-C(19)-C(20)	-178.3(4)
C(18)-C(19)-C(20)-C(21)	1.1(9)
C(19)-C(20)-C(21)-C(22)	-1.1(9)
C(20)-C(21)-C(22)-C(23)	1.3(9)
C(19)-C(18)-C(23)-C(22)	1.4(6)
C(17)-C(18)-C(23)-C(22)	178.4(4)
C(21)-C(22)-C(23)-C(18)	-1.4(7)

Symmetry transformations used to generate equivalent atoms:

Table S7. Hydrogen bonds for **15** (knih122). [Å and °].

D-H...A	d(D-H)	d(H...A)	d(D...A)	<(DHA)
N(2)-H(2A)...Br(1)	0.98	2.36	3.278(2)	156
C(9)-H(9A)...Br(1)#1	0.98	3.11	3.900(3)	139.1
C(9A)-H(9AB)...Br(1)	0.97	3.01	3.813(3)	141.2
O(12)-H(12A)...Br(1)#2	0.82	2.56	3.359(3)	166.4
C(16)-H(16B)...Br(1)#3	0.97	3.1	4.062(3)	174.3

Symmetry transformations used to generate equivalent atoms:

#1 x-1,y,z #2 -x+1/2,-y+2,z+1/2 #3 -x+1,y-1/2,-z+1/2
